# Effects of the Metal Ion on the Mechanism of Phosphodiester Hydrolysis Catalyzed by Metal-Cyclen Complexes

**DOI:** 10.3389/fchem.2019.00195

**Published:** 2019-04-05

**Authors:** Qiaoyu Hu, Vindi M. Jayasinghe-Arachchige, Joshua Zuchniarz, Rajeev Prabhakar

**Affiliations:** Department of Chemistry, University of Miami, Coral Gables, FL, United States

**Keywords:** phosphodiester hydrolysis, metal-cyclen complexes, di- and tetravalent metal ions, reaction mechanisms, density functional theory (DFT)

## Abstract

In this study, mechanisms of phosphodiester hydrolysis catalyzed by six di- and tetravalent metal-cyclen (**M-C**) complexes (**Zn-C, Cu-C, Co-C, Ce-C, Zr-C** and **Ti-C**) have been investigated using DFT calculations. The activities of these complexes were studied using three distinct mechanisms: (1) direct attack (***DA***), (2) catalyst-assisted (***CA***), and (3) water-assisted (***WA***). All divalent metal complexes (**Zn-C, Cu-C** and **Co-C**) coordinated to the BNPP substrate in a monodentate fashion and activated its scissile phosphoester bond. However, all tetravalent metal complexes (**Ce-C, Zr-C**, and **Ti-C**) interacted with BNPP in a bidentate manner and strengthened this bond. The ***DA***mechanism was energetically the most feasible for all divalent **M-C** complexes, while the ***WA***mechanism was favored by the tetravalent complexes, except **Ce-C**. The divalent complexes were found to be more reactive than their tetravalent counterparts. **Zn-C** catalyzed the hydrolysis with the lowest barrier among all **M-C** complexes, while **Ti-C** was the most reactive tetravalent complex. The activities of **Ce-C** and **Zr-C**, except **Ti-C**, were improved with an increase in the coordination number of the metal ion. The structural and mechanistic information provided in this study will be very helpful in the development of more efficient metal complexes for this critical reaction.

## Introduction

**Figure 1:**
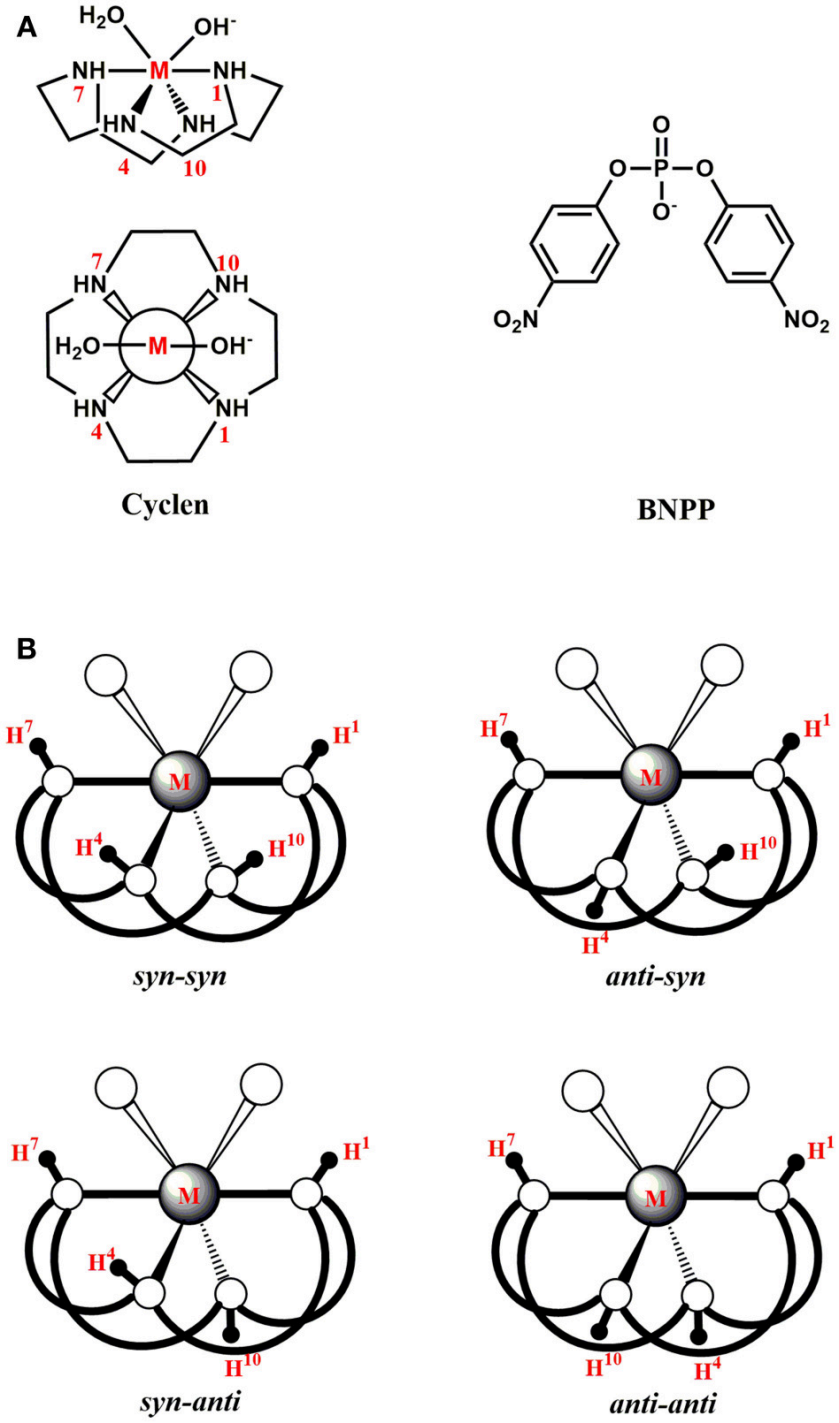
**(A)** Structures of a metal-cyclen (**M-C**) complex (side-view and top-view) and the BNPP substrate. **(B)** Different conformations of a **M-C** complex.

The phosphoester bond [(O=)(RO)(RO)(P-O-R)] is ubiquitous in a wide range of biomolecules such as proteins, nucleic acids, and lipids (Oivanen et al., 1998; Cleland and Hengge, 2006; Neidle, 2008; Kamerlin and Warshel, 2009). For instance, this bond constitutes the backbones of DNA and RNA by connecting the adjacent nucleotides (Sharp, 1985; Robinson et al., 1995; Mikkola et al., 2001; Chandra et al., 2009). It is also present in organophosphorus compounds (OPs) that have been utilized as pesticides and chemical nerve agents (Dubois, 1971; Jeyaratnam, 1990; The, 1998). Thus, the selective hydrolysis of this bond is required in numerous biological and biotechnological applications. In biology, this process has been implicated in DNA repair, post-translational modification of proteins and energy metabolism (Eichler and Lehman, 1977; Sancar and Sancar, 1988; Kia-Ki and Martinage, 1992; Mol et al., 2000). In biotechnology, it is involved in gene sequencing, therapeutics, and bioremediation of pesticides and nerve agents (Gewirtz et al., 1998; Eid et al., 2009; Corda et al., 2014). In nature, three types of phosphoester bonds exist: mono-, di-, and triester (Hadler et al., 2008; Kirby and Nome, 2015). Among them, the phosphodiester bond [(O=)(O^−^)(RO)(P-O-R)] is exceptionally stable with a half-life of approximately 3 × 10^7^ years at room temperature and a neutral pH (Williams and Wyman, 2001). To hydrolyze this bond at biologically relevant rates, ~10^16^ times rate-acceleration, nature has devised highly specialized metalloenzymes known as nucleases and phosphoesterases (Wilcox, 1996; Cowan, 1998; Weston, 2005; Fang et al., 2007). Although these enzymes exhibit remarkable activities, they suffer from several limitations such as undesirable selectivity, difficulties in extraction or synthesis, high cost and narrow functional temperature, and pH range (Kövári and Krämer, 1996; Cowan, 2001; Mancin et al., 2012). Therefore, in the last couple of decades, intensive efforts have been made to design small metal complexes as synthetic analogs of natural enzymes for phosphoester hydrolysis (Burstyn and Deal, 1993; Hegg and Burstyn, 1998; Komiyama and Sumaoka, 1998; Blaskó and Bruice, 1999; Williams et al., 1999; Sreedhara and Cowan, 2001; Deck et al., 2002; Mitić et al., 2006; Niittymaki and Lonnberg, 2006; Bonomi et al., 2008; Krauser et al., 2010; Mancin et al., 2012; Daver et al., 2016; Sullivan et al., 2018). These analogs can offer multiple advantages over natural enzymes in terms of cost, size, and functionality (Weston, 2005; Yoji et al., 2006). To advance this goal, among others, several chemically distinct polyazamacrocyclic ligands were synthesized through the Stetter-Richman-Atkins method (Richman and Atkins, 1974; Weisman and Reed, 1996). In particular, 1,4,7,10-tetraazacyclododecane (cyclen, **C**) and its derivatives containing mononuclear metal complexes have been utilized for phosphodiester and peptide hydrolysis (Figure 1A) (Koike et al., 1994; Shionoya et al., 1994; Hettich and Schneider, 1997; Chae et al., 2005; Fang et al., 2007; Junghun et al., 2007; Subat et al., 2008; Zhang et al., 2014, 2016b). In most studies of phosphodiester hydrolysis, the bis(4-nitrophenyl) phosphate (BNPP) molecule has been used as a model of DNA (Figure 1A). Koike and Kimura (1991) investigated BNPP hydrolysis by the Zn(II)-cyclen (**Zn-C**) complex and reported the pseudo-first order rate constant of 2.8 × 10^−9^ s^−1^ at 35°C and pH 7. This complex provided a 46-fold rate acceleration compared to the background reaction. The Co(III)-cyclen based complexes (cyclen attached to polystyrene or methyl benzoate) also hydrolyzed the phosphodiester bond of DNA and RNA efficiently (Jeung et al., 2001; Delehanty et al., 2005). The polystyrene complex decreased the half-life of supercoiled DNA to 40 min at 4°C, while the one with methyl benzoate promoted the hydrolysis of ~96% of mRNA population within 24 h at 25°C. Furthermore, a Cu(II)-cyclen analog with two pyridine subunits was shown to degrade supercoiled DNA with k_cat_ = 2.31 × 10^−3^ min^−1^ under physiological conditions (Li et al., 2007).

Additionally, several tri- and tetravalent lanthanides (Eu, La, Zr, and Ce) containing complexes have been reported to hydrolyze the phosphoester bond (Rammo et al., 1996; Baykal et al., 1999; Franklin, 2001; Gómez-Tagle and Yatsimirsky, 2001; Luedtke and Schepartz, 2005). Fanning et al. (2006) synthesized several cyclen based trivalent Eu(III) and La(III) complexes that hydrolyzed 2-hydroxypropyl 4-nitrophenyl phosphate (HPNP, an RNA model compound) within the physiological pH range. Furthermore, tetravalent Ce(IV) in an aqueous solution provided 10^11^ times rate-acceleration over the background reaction and 20–1000 times over the trivalent lanthanides for DNA hydrolysis (Komiyama et al., 1999). However, the exact nature of the active complexes in this reaction was not known. Nonetheless, based on the X-ray absorption fine structure data, remarkable activity of the Ce complex was proposed to be promoted by a weak covalent interaction between Ce(IV) and the phosphate group of the substrate (Hidemi et al., 1999).

In the proposed mechanism of phosphoester hydrolysis, the metal ion has been suggested to play the following key roles: (1) Lewis acid activation of the substrate, (2) creation of a nucleophile, and (3) generation of a good leaving group of the substrate (Chin, 1991; Bashkin and Jenkins, 1994; Fothergill et al., 1995; Williams et al., 1999; Das et al., 2013). Additionally, it stabilizes the transition states and intermediates by neutralizing their negative charges. To accomplish these functions, a metal ion should possess high Lewis acidity, strong nucleophilicity, redox stability, borderline hardness and low ligand field stabilization energy (Wilkinson et al., 1987; Hegg and Burstyn, 1998). However, an increase in its Lewis acidity causes a decrease in the nucleophilicity and these two effects require the right balance for the optimum reactivity (Koike and Kimura, 1991; Kimura et al., 1995; Bonfá et al., 2003; Coleman et al., 2010).

The metal-cyclen (**M-C**) complex can exist in equilibrium between several diastereoisomers (*sys-syn, anti-syn, syn-anti*, and *anti-anti*) associated with the orientation of protons (H^4^ or H^10^) on the nitrogen atoms of the cyclen macrocycle (Figure 1B; Hay and Norman, 1997). The H^4^ or H^10^ atoms face the substrate in the *syn* conformation, while they are located on the opposite side of the substrate in the *anti* conformation. The NMR data showed that *syn-anti* conformation of [Co(cyclen)Cl_2_]^+^ was more stable than other conformations (Sosa and Tobe, 1985). The X-ray structure of [Co(cyclen)(NO_2_)_2_]^+^ exhibited that this complex also existed in the *syn-anti* conformation (Iitaka et al., 1974). Additionally, the X-ray structures of both [Co(cyclen)(NH_3_)_2_]^3+^ and [Co(cyclen)(diamine)]^3+^ (diamine = H_2_N(CH_2_)_2_NH_2_, H_2_N(CH_2_)_3_NH_2_) complexes were crystallized in the *syn-anti* conformation (Clarkson et al., 2000). However, the exact conformation of a **M-C** complex has been proposed to depend on the nature of the metal ion (Zhang et al., 2014).

**Figure 2:**
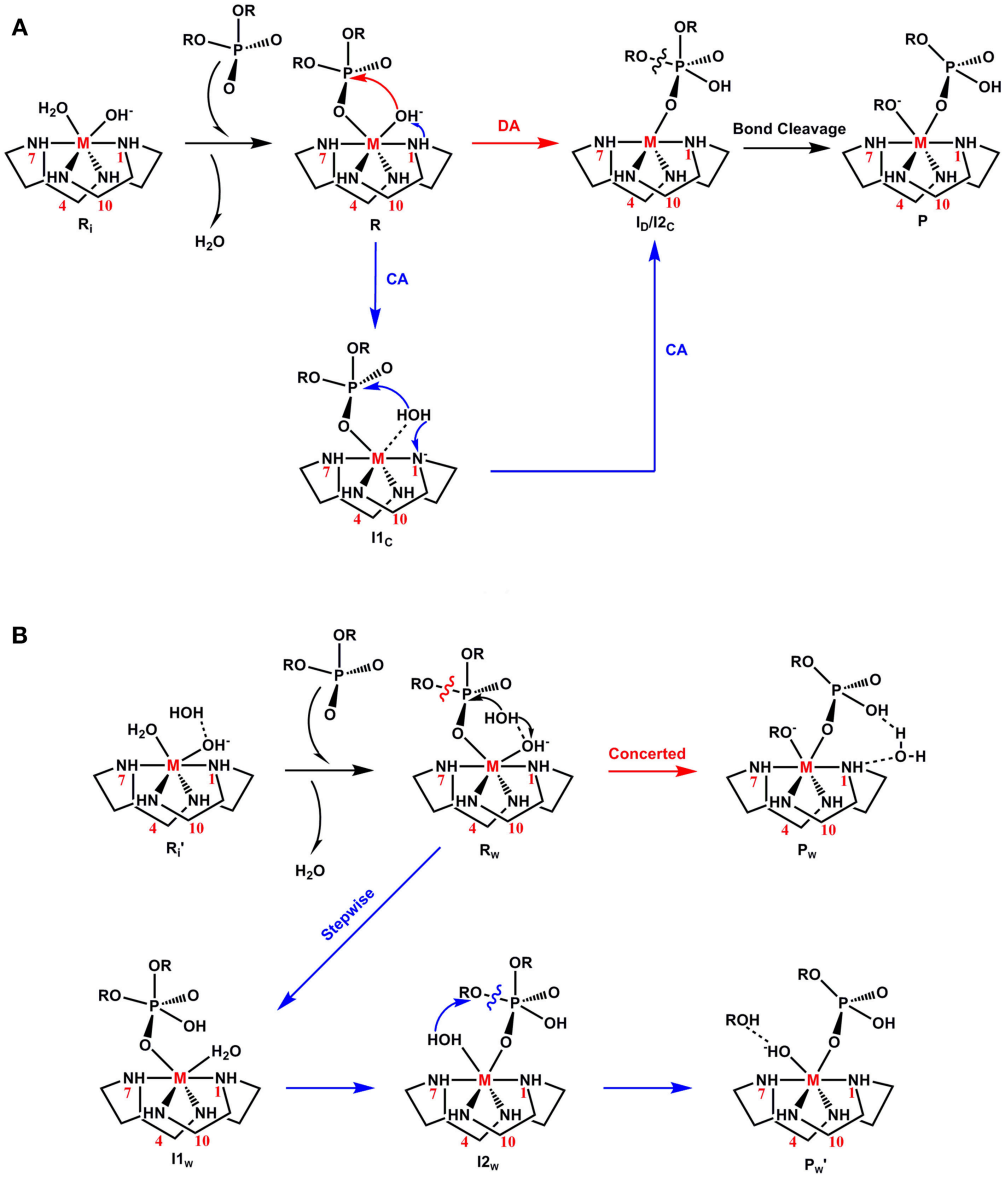
Proposed mechanisms for phosphoester hydrolysis: **(A)** Direct-attack (***DA***) and Catalyst-assisted (***CA***) and **(B)** Water-assisted (**WA**).

The experimentally proposed mechanism, termed direct attack (***DA***), utilized by metal complexes for the phosphodiester hydrolysis is shown in Figure 2A (Hendry and Sargeson, 1989; Komiyama et al., 1999; Mancin and Tecilla, 2007). In the initial form of the **M-C** complex (**R**_**i**_), the metal ion is coordinated to the cyclen macrocycle, a hydroxyl ion and a water molecule (Kim et al., 2009). The protonation states of the hydroxyl ion and water molecule were based on the measured p*K*_a_ values of the **Co-C** complex (p*K*_a1_ = 5.66 and p*K*_a2_ = 8.14) (Kim et al., 2009). According to this mechanism, from **R**_**i**_, substitution of the metal-bound water molecule by the substrate creates an active complex (**R**). In the next step, a nucleophilic attack by the metal-bound hydroxyl group on the phosphorus center generates a five-membered phosphorane intermediate (**I**_**D**_). In the final step, the P-OR bond *trans* to the nucleophile is cleaved to form the final product (**P**).

Recently, based on DFT calculations, another mechanism called catalyst-assisted (***CA***) was proposed for the **Cu-C** complex (Figure 2A; Zhang et al., 2016b). According to this mechanism, the metal-bound hydroxide functions as a base and abstracts a proton from the nitrogen atom (N^1^) of the cyclen to generate a water molecule (**I1**_**C**_). In the next step, the N^1^ atom acts as a base and accepts the previously donated proton. The hydroxide nucleophile created in this process attacks the electrophilic phosphorus atom of BNPP to form an intermediate (**I2**_**C**_). From **I2**_**C**_, the cleavage of the P-O bond can occur spontaneously and the charged leaving group (RO^−^) coordinates to the metal ion in the product (**P**).

Additionally, BNPP hydrolysis could occur through a third mechanism, termed a water assisted (***WA***) mechanism (Figure 2B; Dal Peraro et al., 2004; Jayasinghe-Arachchige et al., 2019). According to this mechanism, an external water molecule is employed for the nucleophilic attack and/or leaving group departure. After the formation of the reactant (**R**_**w**_), the metal-bound hydroxide functions as a base and abstracts a proton from a solvent water molecule to generate a free nucleophile (-OH). This hydroxyl nucleophile concomitantly attacks the BNPP substrate. Depending on the nature of the metal ion (di- or tetravalent), this mechanism could also occur in a stepwise manner after this step. In this pathway, the metal-bound water molecule assists the cleavage of the phosphoester bond and creates a neutral leaving group (ROH).

Quite clearly, the metal-bound hydroxyl group play different roles in these mechanisms: (1) nucleophile only (***DA***mechanism), (2) both base and nucleophile (***CA***mechanism), and (3) base only (***WA***mechanism). The rate of this reaction is likely to depend on the stability of the rate-limiting transition state, which is connected with the Lewis acidity of the metal ion and the geometry of the metal-BNPP complex.

Despite the availability of a wealth of experimental and theoretical information, several unresolved issues concerning the exact mechanism, structures and roles of the metal ion still remain. For example, the conformation of the substrate bound **M-C** complexes (*sys-syn, anti-syn, syn-anti*, and *anti-anti*) for different metals (di- and tetravalent) is not known experimentally. The structures (transition states and short-lived intermediates) and energetics of the reaction mechanism for a specific metal ion are also not available. We have addressed all these important issues for a variety of **M-C** complexes using two sets of metal ions, divalent [Zn(II), Cu(II), and Co(II)] and tetravalent [Ce(IV), Zr(IV), and Ti(IV)], for BNPP hydrolysis through all three (***DA*,** ***CA*, **and ***WA***) mechanisms. The available experimental and theoretical information has been fully integrated in these calculations. These results will provide intricate details of the metal assisted phosphodiester hydrolysis and pave the way for the design of the next generation of synthetic metallohydrolases to catalyze this critical reaction.

## Computational Details

### Method

All Density Functional Theory (DFT) calculations were performed using the Gaussian 09 program package (Frisch et al., 2009). The geometry optimizations of reactants, transition states, intermediates and products were conducted using the B3LYP functional (Becke, 1988, 1993) without any constraints. Mixed basis sets were utilized for the structure optimization and frequency analysis. In particular, the Stuttgart relativistic effective core potential (ECP) basis set (RSC97) (Lee et al., 1988; Dolg et al., 1989) was applied for the metal ions. This is a double zeta basis set that uses 28 core electrons ([Ar]+3d) for the second-row transition metals and the lanthanides and 10 core electrons ([Ne]) for the first row transition metals. The 6-311G(d,p) basis set was used for the O, N and P atoms, while 6-31G was used for C and H atoms (Ditchfield et al., 1971). The final energies of the optimized structures were further improved by performing single point calculations using a bigger triple zeta quality 6-311+G(d,p) basis set for P, O, N, C and H atoms and RSC97 for metal ions. Hessians were calculated at the same level of theory as the optimizations to confirm the nature of the stationary points along the reaction coordinates. The transition states were confirmed to have only one negative eigenvalue corresponding to the reaction coordinates. The intrinsic reaction coordinate (IRC) approach (Ischtwan and Collins, 1988) that connects a transition state to the corresponding minima was utilized. The natural atomic charge for each atom was calculated by natural bond orbital (NBO) analysis using the NBO version 3 (Foster and Weinhold, 1980; Reed and Weinhold, 1983). Solvent effects for water (dielectric constant = 78.39) were calculated utilizing the polarizable continuum model (PCM) using the integral equation formalism variant (IEFPCM), which is a default self-consistent reaction field (SCRF) method (Cancès et al., 1997). The B3LYP energies were compared with the energies calculated using the M06L (Zhao and Truhlar, 2006), MPW1PW91 (Adamo and Barone, 1998) and PBE1PBE (Perdew et al., 1996) functionals. All energy barriers using these functionals were within 2.7–3.6 kcal/mol and provided similar potential energy surfaces (PES). The final energies computed at the B3LYP/6-311+G(d,p) level including zero-point vibrational (unscaled), thermal (298.15 K, 1 atm), entropy corrections (298.15 K) and solvent effects were used to discuss the activities of all **M-C** complexes. The measured *k*_cat_ values were converted into activation energy using the Arrhenius equation (k = Ae^−Ea/RT^, where A is the pre-exponential factor, Ea is the activation energy, R is the gas constant and T is the temperature). It is noteworthy that calculations were performed at room temperature (25°C), while some *k*_cat_ values were measured at a higher temperature. Due to the temperature dependence of the pre-exponential constant in the Arrhenius equation, it was not possible to accurately estimate the measured barrier at 25°C.

### Models

In the calculations, the metal ion was coordinated to the 1,4,7,10-tetraazacyclododecane (cyclen) ligand, a hydroxyl ion and water molecules (Chin et al., 1989; Kim et al., 2009). The number of water molecules was determined by the chemical nature of the metal ion and the underlying mechanism. The overall charge for the **Zn-C**, **Cu-C**, and **Co-C** complexes was 0, while for **Ce-C**, **Zr-C**, and **Ti-C** the charge was +2. **Cu-C** and **Co-C** existed in the doublet spin state, while all the other **M-C** complexes existed in the singlet spin state. BNPP was used as the model of DNA because it contains two nitrophenyl groups which are similar to the deoxyribose rings of DNA.

## Results and Discussion

The activities of all six **M-C** complexes (**Zn-C**, **Cu-C**, **Co-C**, **Ce-C**, **Zr-C**, and **Ti-C**) were investigated using three different mechanisms: (1) direct attack (***DA***), (2) catalyst-assisted (***CA***), and (3) water-assisted (***WA***), Figure 2. Their energetics were compared using the metal-ligand, metal-nucleophile and P-O bond lengths, strain of the cyclen ring, atomic charges and coordination number of metal ions as parameters. The Lewis acidity and nucleophilicity of the metal ions can be qualitatively characterized by the metal-substrate and metal-nucleophile bond lengths (Bertini et al., 1990; Coleman et al., 2010). In this section, the ***DA, CA***and ***WA***mechanisms were first discussed for the divalent metal complexes [**Zn-C**, **Cu-C**, and **Co-C**] followed by for the tetravalent complexes [**Ce-C, Zr-C** and **Ti-C**].

The starting point of all these mechanisms was the BNPP substrate bound structure of the **M-C** complexes. The *syn-syn* conformation was found to be the energetically most stable for all six metals (Figure 1B). The other conformations were 1.8–27.9 kcal/mol higher in energy. The relative stability of the *syn-syn* structure could be due to its lower strain computed as the sum of the N^1^-M-N^7^ and N^4^-M-N^10^ angles (Figure 1). This conformation possessed the largest angle (204.22–255.99°), and least strain, in comparison to the other three conformers. Additionally, the H^4^ and H^10^ atoms of the cyclen formed hydrogen bonds with the phosphate group of BNPP to provide extra stabilization to this conformation.

### Phosphodiester Hydrolysis by Divalent Metal-Cyclen (M-C) Complexes

The divalent Zn, Cu, and Co ions are known to form stable complexes with a common coordination number of six for the phosphodiester and peptide hydrolysis (Holm et al., 1996; Berreau, 2006; Jang and Suh, 2008; Chei et al., 2011). Therefore, the BNPP substrate could only be singly coordinated to these metal ions (P-O-M mode), and the remaining coordination sites were occupied by four nitrogen atoms (N^1^, N^4^, N^7^, and N^10^) of the cyclen ligand and one hydroxyl group. In this section, for the sake of clarity, all three mechanisms for **Zn-C** were discussed in detail followed by comparisons with the **Cu-C** and **Co-C** complexes.

#### Direct Attack (DA) Mechanism

**Figure 3:**
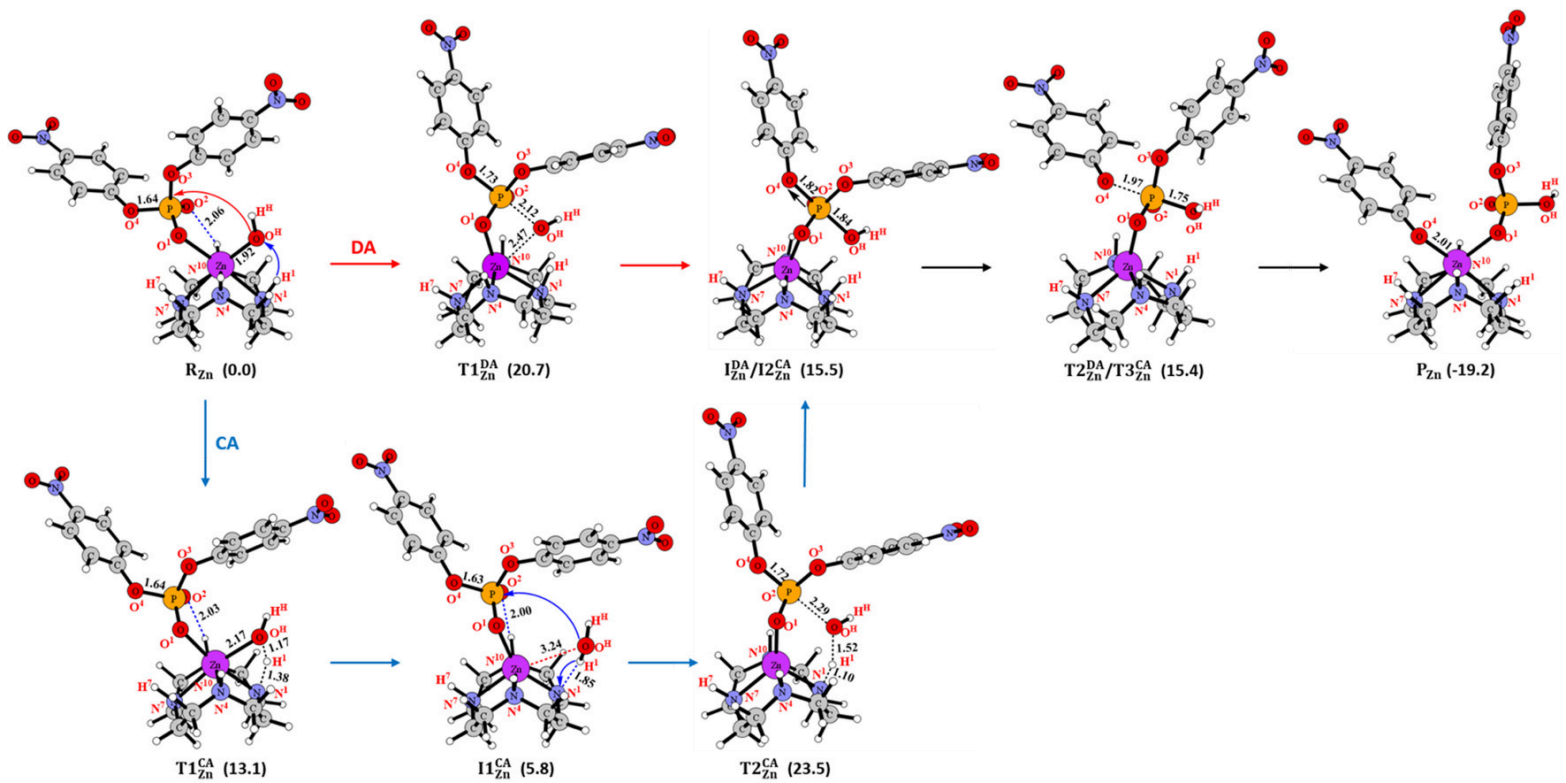
Structures (in Å) and energies (in kcal/mol) in the ***DA***and ***CA***mechanisms for **Zn-C**.

In the reactant (**R**_**Zn**_ in Figure 3) of **Zn-C**, one phosphoryl oxygen (O^1^) atom of BNPP was bound to the Zn(II) ion, while the other one (O^2^) interacted with the -N^10^H group of the cyclen through a hydrogen bond. This metal-substrate coordination elongated the scissile P-O^4^ bond of BNPP by 0.04 Å in comparison to this bond in its free form (P-O^4^ = 1.64 Å in Figure 3). In **R**_**Zn**_, the Lewis acidity of the Zn ion played a key role in the activation of the P-O^4^ bond. The interaction between the *p* orbital of the oxygen atom with the *d* orbital of the Zn atom promoted this activation (Figure S1). In the first step, the Zn-bound -O^H^H^H^ nucleophile directly attacked the electrophilic P atom of the substrate to generate a five-membered trigonal bipyramidal phosphorane intermediate (${\text{I}}_{Zn}^{DA}$). This process occurred with a barrier of 20.7 kcal/mol and in the optimized transition state (${\text{T}1}_{Zn}^{DA}$) the Zn-O^H^H^H^ and P-O^4^ bonds, *trans* to the nucleophile, became substantially longer by 0.55 and 0.09 Å, respectively (Figure 3 and Table S1). ${\text{I}}_{Zn}^{DA}$ was endergonic by 15.5 kcal/mol from **R**_**Zn**_ and the P-O^4^ bond was significantly activated but not completely broken in this intermediate (P-O^4^ = 1.82 Å). However, this bond was cleaved in the next step and the nitrophenolate (-OC_6_H_4_NO_2_) group was released. In the transition state (${\text{T}2}_{Zn}^{DA}$) for this process, the P-O^4^ bond was significantly elongated to 1.97 Å (Figure 3). The negatively charged nitrophenolate group generated in this process was coordinated to the Zn ion in the product (**P**_**Zn**_). **P**_**Zn**_ was computed to be exergonic by 19.2 kcal/mol from **R**_**Zn**_. The overall barrier (20.7 kcal/mol) for this mechanism was somewhat underestimated in comparison to the measured barrier of 29.3 kcal/mol (computed from the *k*_cat_ value using the Arrhenius equation) for BNPP hydrolysis by **Zn-C** (Koike and Kimura, 1991).

The overall energetics of this mechanism for **Cu-C** and **Co-C** were quite comparable to that of **Zn-C** (Figures S2, S3). However, the barrier (22.0 and 23.4 kcal/mol) and endergonicity (17.7 and 17.2 kcal/mol) of the rate-limiting first step were slightly higher for **Cu-C** and **Co-C**, respectively, in comparison to **Zn-C**. This difference could be due to the greater strength of the Zn-bound nucleophile in **R**_**Zn**_ i.e., longer Zn-O^H^ distance and higher charge on the O^H^ atom (Tables S1, S2).

These results suggested that both Lewis acidity and nucleophilicity of the metal center controlled the energetics of this mechanism. **Zn-C** was found to be slightly more reactive than the **Cu-C** and **Co-C** complexes.

#### Catalyst-Assisted (CA) Mechanism

The catalyst-assisted (***CA***) mechanism (Figure 3; Zhang et al., 2016b) started with the same reactant (**R**_**Zn**_) as the ***DA***mechanism. In the first step, the Zn-bound hydroxyl group of **R**_**Zn**_ functioned as a base and abstracted the H^1^ proton from the cyclen ring to form a water molecule (O^H^H^H^H^1^). This process occurred with a barrier of 13.1 kcal/mol and the Zn-O^H^ bond was extended by 0.25 Å in the optimized transition state (${\text{T}1}_{Zn}^{CA}$) in comparison to the corresponding distance in **R**_**Zn**_. The intermediate (${\text{I}1}_{Zn}^{CA}$) formed in this process was 5.8 kcal/mol endergonic from **R**_**Zn**_ (Figure 3). In ${\text{I}1}_{Zn}^{CA}$, the water molecule was not coordinated to the Zn ion and associated with the N^1^ atom of the cyclen ligand through a hydrogen bond. The strain of the cyclen ring and the acidity of the -N^1^H^1^ group controlled the energetics of this step. The sum of the N^1^-M-N^7^ and N^4^-M-N^10^ angles (238.6° in **R**_**Zn**_) was increased by 15.3° in ${\text{T}1}_{Zn}^{CA}$ (253.9°) i.e., less strain in ${\text{T}1}_{Zn}^{CA}$. However, a high charge (−0.77*e*) on the N^1^ atom of the cyclen ligand also lowered the acidity of the H^1^ atom. From ${\text{I}1}_{Zn}^{CA}$, the cyclen ligand directly participated in the mechanism by creating a nucleophile and regenerating the ligand through activation of the previously generated water molecule (O^H^H^H^H^1^ in Figure 3). In this step, the N^1^ atom of the ligand abstracted the H^1^ proton concomitantly with the attack of the -O^H^H^H^ nucleophile to the electrophilic P atom of BNPP. This concerted process, through transition state (${\text{T}2}_{Zn}^{CA}$), led to the creation of the phosphorane intermediate (${\text{I}2}_{Zn}^{CA}$) that was endergonic by 15.5 kcal/mol (Figure 3). From **R**_**Zn**_, this step occurred with a barrier of 23.5 kcal/mol and found to be the rate-limiting step of the ***CA***mechanism. At ${\text{I}2}_{Zn}^{CA}$, both ***DA***and ***CA***mechanisms merged and led to the generation of the common product (**P**_**Zn**_ in Figure 3).

The structures of the reactants for **Cu-C** and **Co-C** (**R**_**Cu**_ and **R**_**Co**_, respectively) were quite similar to **R**_**Zn**_ (Figures S2, S3). However, the barrier of the first step for **Cu-C** and **Co-C** was lowered by 1.4 and 4.4 kcal/mol, respectively, in comparison to the barrier for **Zn-C**. Additionally, the phosphorane intermediate for these systems was found to be more stable by 4.8 and 4.3 kcal/mol, respectively (Figures S2, S3). These energy differences were likely to be caused by lower strain of the cyclen ring i.e., 238.6°, 245.0°, and 256.0° For **R**_**Zn**_, **R**_**Cu**_, and **R**_**Co**_, respectively. Additionally, a lower charge on the N^1^ atom increased the acidity of the -N^1^H^1^ group of **Cu-C** and **Co-C** and made this process energetically more favorable (Table S2). The computed barrier of the next rate-determining step for **Cu-C** and **Co-C** (24.3 and 24.6 kcal/mol, respectively) was slightly higher than the barrier for **Zn-C** (23.5 kcal/mol). Here, due to the higher basicity of the N^1^ atom in **Zn-C**, the proton transfer occurred with a lower barrier.

These results suggested that the ***CA***mechanism was energetically less favorable than the ***DA***mechanism for all divalent metals. Due to the direct involvement of the cyclen ligand, the strain of the cyclen ring and acidity of the N^1^H^1^ group determined the energetics of the ***CA***mechanism. Similar to the ***DA***mechanism, **Zn-C** was more reactive than **Cu-C** and **Co-C**.

#### Water-Assisted (WA) Mechanism

**Figure 4:**
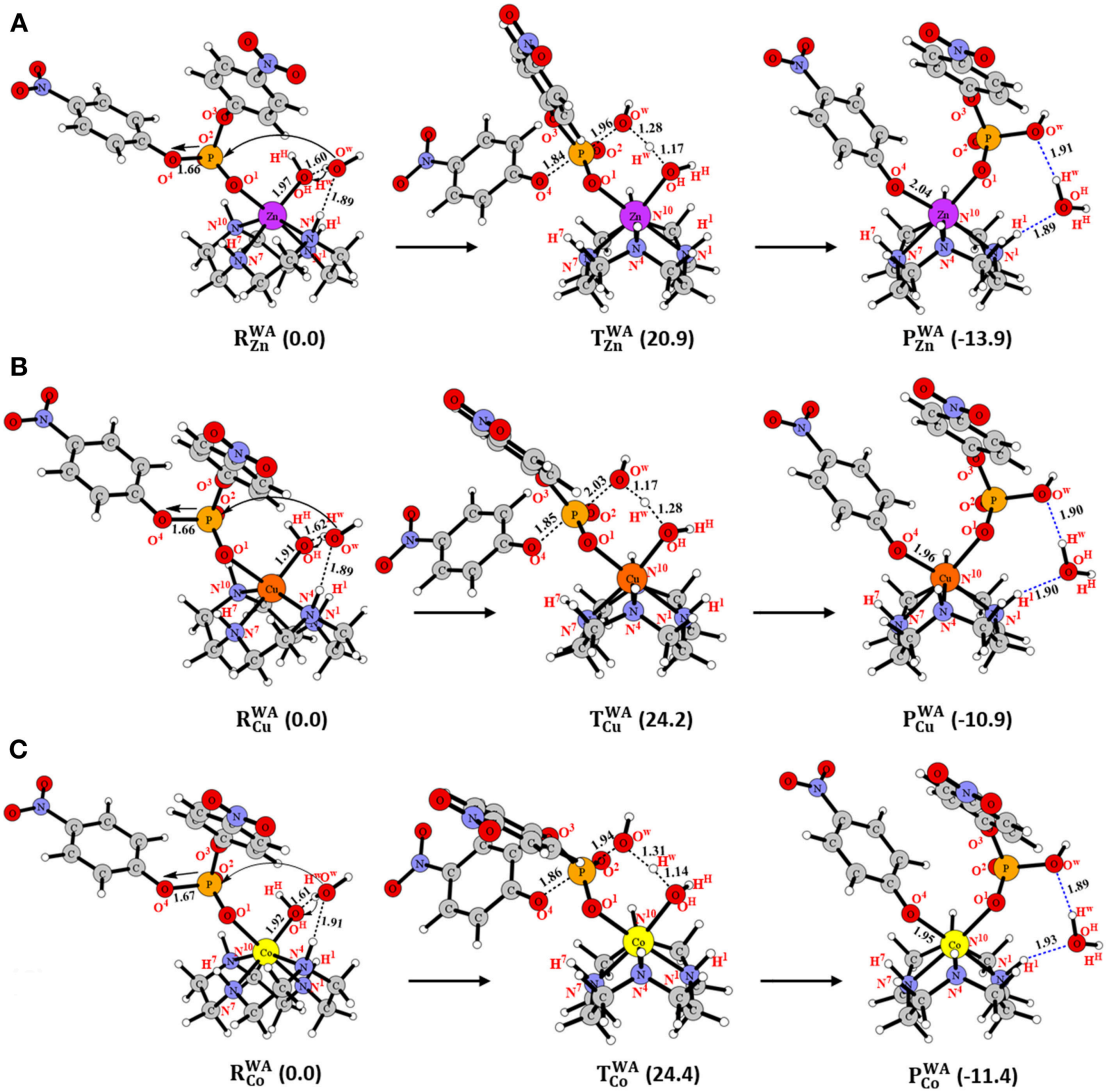
Structures (in Å) and energies (in kcal/mol) in the ***WA***mechanism for **(A) Zn-C**, **(B) Cu-C** and **(C) Co-C**.

The major difference between the ***DA***and ***WA***mechanisms is that in the latter, the metal-bound hydroxide played the role of a base and created a nucleophile through activation of an external water molecule (Figure 2B). In the reactant (${\text{R}}_{Zn}^{WA}$) of **Zn-C**, the Zn-bound hydroxyl group (-O^H^H^H^) interacted with an external water molecule (HO^w^H^w^) through a hydrogen bond (Figure 4A). This interaction elongated the Zn-O^H^H^H^ bond by 0.05 Å in comparison to **R**_**Zn**_. From ${\text{R}}_{Zn}^{WA}$, the Zn-bound hydroxyl (-O^H^H^H^) abstracted the H^w^ proton of the external water molecule and generated the free -O^w^H nucleophile that concomitantly attacked the BNPP substrate and cleaved the P-O^4^ bond. This concerted process occurred through transition state (${\text{T}}_{Zn}^{WA}$) with a barrier of 20.9 kcal/mol (Figure 4A and Table 1). In the product (${\text{P}}_{Zn}^{WA}$), the negatively charged -OC_6_H_4_NO_2_ group created by the nucleophilic attack coordinated to the Zn ion with the release of the water molecule (H^w^O^H^H^H^). ${\text{P}}_{Zn}^{WA}$ was 13.9 kcal/mol exergonic from ${\text{R}}_{Zn}^{WA}$. The strength of the hydroxyl nucleophile generated from an external water was weaker than that of a metal-bound nucleophile. However, quite surprisingly, the barrier for the ***WA***mechanism (20.9 kcal/mol) was quite comparable to the one computed for the ***DA***mechanism (20.7 kcal/mol). That could be due to the extra stability of the five-membered transition state (${\text{T}}_{Zn}^{WA}$) formed in the former, in comparison to the four-membered transition state (${\text{T}1}_{Zn}^{DA}$) created in the latter. For **Cu-C** and **Co-C** the barrier for the ***WA***mechanism was also slightly higher by 2.2 and 1.0 kcal/mol, respectively, than for the ***DA***mechanism (Figures 4B,**C**). However, the barrier for the ***WA***mechanism for **Cu-C** and **Co-C** was 3.3 and 3.5 kcal/mol, respectively higher than the barrier for **Zn-C** i.e., 24.2 and 24.4 kcal/mol, respectively (Figure 4). This difference was likely to be due to the stronger basicity of the Zn-O^H^H^H^ group among all three complexes. It was caused by the longer M-O^H^ distance and higher charge on the O^H^ atom in **Zn-C** (Tables S1, S2). Additionally, in contrast to the singlet spin state of Zn in **Zn-C**, both Cu and Co existed in the doublet spin state in **Cu-C** and **Co-C**. That might also be the reason for the similar energetics of **Cu-C** and **Co-C**.

According to these results, the basicity of the M-O^H^ group influenced the energetics of the ***WA***mechanism. Additionally, energetics of all three mechanisms (***DA***, ***CA*, **and ***WA***) were quite comparable for all divalent metal complexes. Furthermore, **Zn-C** was found to be more reactive than **Cu-C** and **Co-C** for all three mechanisms.

### Phosphodiester Hydrolysis by Tetravalent Metal-Cyclen (M-C) Complexes

The tetravalent metals (Ce, Zr, and Ti) form complexes with higher coordination numbers 6–12, than the divalent metals (Zn, Cu, and Co) with coordination numbers 5–6. Among tetravalent metals, Ce can form complexes with coordination numbers 7–12, while Zr and Ti with 6–8 (Komiyama et al., 1999; Bonomi et al., 2010). Here, due to the difference in their coordination number, all three ***DA***, ***CA*, **and ***WA***mechanisms are first discussed for **Ce-C**, followed by for **Zr-C** and **Ti-C**.

#### Direct Attack (DA) Mechanism for the Ce-C Complex

The activity of **Ce-C** was studied using three different coordination numbers (7–9). Due to the steric hindrance, complexes with higher coordination numbers (10–12) could not be optimized. In the reactant (**R**_**Ce**_) with coordination number 7, BNPP was coordinated to the Ce ion through the O^1^ and O^2^ atoms in the bidentate form. In contrast, BNPP binding to the divalent metals occurred in the monodentate fashion. As a result, the scissile P-O^4^ bond became stronger by 0.03 Å in **Ce-C** (Figure S5). In **R**_**Ce**_, all metal-ligand distances (Table S3) were significantly longer than those in the reactant of **Zn-C** (Table S1). The excellent hydrolytic activity of the Ce ion in aqueous solution was reported to be due to the hybridization of the 4*f* orbitals of Ce with the 2*p* orbitals of the coordinated oxygen atoms of the substrate (Komiyama, 2016). However, Ce can form complexes with different coordination numbers in the solution, and the actual active complex in the previous study was not known. Here, substrate-bound mononuclear **Ce-C** complex was not found to activate the P-O^4^ bond (Figure S4). The direct nucleophilic attack of the metal-bound hydroxide (-O^H^H^H^) to BNPP occurred with a barrier of 39.1 kcal/mol (Figure S5), which was almost twice the barrier computed for **Zn-C**. The reason for this significantly higher barrier was the change of **Ce-C** from a hepta-coordinated (coordination number 7) **R**_**Ce**_ to an unfavorable hexa-coordinated (coordination number 6) ${\text{T}1}_{Ce}^{DA}$. From ${\text{I}}_{Ce}^{DA}$, the P-O^4^ bond was completely broken with a small barrier of 2.1 kcal/mol and the separated nitrophenolate (-OC_6_H_4_NO_2_) and phosphate [-(O)_2_P(OC_6_H_4_NO_2_)OH] groups were coordinated to the Ce(IV) ion in the product (**P**_**Ce**_ in Figure S5).

This mechanism was further studied by including extra water as a ligand (coordination number of Ce = 8). It was also suggested in our previous study (Zhang et al., 2017) that an increased coordination number of the metal ion enhanced the peptidase activity of the Zr(IV) azacrown ether complex [Zr-(NO_2_)(OH^H^)(H_2_O)_n_]. In the reactant (**R'**_**Ce**_) with coordination number 8 (Figure S6), the additional Ce-bound water formed a hydrogen bond with BNPP. From **R'**_**Ce**_, the barrier for the nucleophilic attack in the rate-determining first step was lowered slightly by 1.4 kcal/mol i.e., 37.7 kcal/mol from **R'**_**Ce**_. The inclusion of the second water molecule (coordination number of Ce = 9) further lowered the barrier for this step by 1.5 kcal/mol i.e., 36.2 kcal/mol from the reactant (**R”**_**Ce**_ in Figure S7). This slight reduction of barrier upon increasing the coordination number of Ce (7–9) could be caused by a slight increase in the metal-nucleophile (Ce-O^H^) distance and a decrease in the charge on the Ce atom (Tables S3, S4). This indicates the provision of a stronger nucleophile in the complexes with a higher coordination number.

These results suggested that, in comparison to the divalent metals, the lower activity of all **Ce-C** complexes with different coordination numbers (7–9) was caused by the strengthening of the scissile phosphoester bond and weaker nucleophilicity of the hydroxyl nucleophile. However, the reactivity of the **Ce-C** complex was slightly enhanced with an increase in the coordination number (7–9) of the Ce ion.

#### Catalyst-Assisted (CA) Mechanism for the Ce-C Complex

In this mechanism, from **R**_**Ce**_, an abstraction of the H^1^ proton of the cyclen ring by the Ce-bound hydroxyl (-O^H^H^H^) took place with a barrier of 26.9 kcal/mol (Figure S5). Similar to the ***DA***mechanism, the barrier for this process was 13.8 kcal/mol higher than the barrier for **Zn-C** (Figure 3). The metal-nucleophile (Ce-O^H^) distance was significantly elongated from 2.04 Å to 2.33 and 2.50 Å in the transition state ($\text{T}1_{Ce}^{CA}$) and the intermediate ($\text{I}1_{Ce}^{CA}$), respectively, in comparison to **R**_**Ce**_. In the next step, the reverse transfer of the H^1^ proton from the Ce-bound water molecule (H^H^O^H^H^1^) to the N^1^ atom of the cyclen and simultaneous nucleophilic attack of the hydroxide (-O^H^H^H^) to BNPP generated a phosphorane intermediate ($\text{I}2_{Ce}^{CA}$). This synchronous process occurred in the rate-limiting step with a barrier of 46.8 kcal/mol and ${\text{I}2}_{Ce}^{CA}$ was endergonic by 35.5 kcal/mol from **R**_**Ce**_. After its formation, the product (**P**_**Ce**_) was generated through the cleavage of the P-O^4^ bond (Figure S5).

The inclusion of an additional water molecule to this complex increased the coordination number of Ce to 8 and lowered the barrier for the rate-limiting step by 2.3 kcal/mol i.e., 44.5 kcal/mol from the corresponding reactant (**R'**_**Ce**_ in Figure S6). The inclusion of the second water molecule (coordination number of Ce = 9) further lowered the barrier only by 0.5 kcal/mol i.e., 44.0 kcal/mol from the reactant (**R”**_**Ce**_ in Figure S7). This lowering of the barrier could be attributed to an increase in the Ce-O^H^H^H^ (nucleophile) distance (Table S3) and a reduction in charge of the Ce atom (Table S4).

Similar to divalent metal-complexes, the ***CA***mechanism was found to be energetically less favorable than the ***DA***mechanism and the energetics of this mechanism improved slightly with an increase in the coordination number of Ce.

#### Water-Assisted (WA) Mechanism for the Ce-C Complex

The reactant (${\text{R}}_{Ce}^{WA}$) of the ***WA*** mechanism was similar to **R**_**Ce**_, except for an external water molecule that was hydrogen bonded to the Ce-bound hydroxyl (-O^H^H^H^) and BNPP (Figure S8). In the first step the nucleophile (-O^1w^H), generated through the abstraction of a proton (H^1w^) by the Ce-O^H^H^H^ group simultaneously attacked BNPP. This process took place with a barrier of 43.5 kcal/mol and led to the creation of the phosphorane intermediate (${\text{I}}_{Ce}^{WA}$). In ${\text{I}}_{Ce}^{WA}$, the scissile P-O^4^ bond was substantially activated to 1.81 Å but not completely broken. In the next step, this bond was cleaved with a small barrier of 2.2 kcal/mol i.e., 44.3 kcal/mol from ${\text{R}}_{Ce}^{WA}$. The nitrophenolate group bound product (${\text{P}}_{Ce}^{WA}$) was computed to be exergonic by 4.7 kcal/mol from ${\text{R}}_{Ce}^{WA}$.

As observed previously, the inclusion of an additional water molecule (coordination number of Ce = 8) lowered the barrier for the rate-limiting second step by 2.8 kcal/mol i.e., 41.5 kcal/mol from the corresponding reactant (${R^{\prime }}_{Ce}^{WA}$, Figure S9). The addition of the second water molecule (coordination number of Ce = 9) further lowered this barrier by 4.5 kcal/mol i.e., 37.0 kcal/mol from the reactant (${R^{\prime \prime }}_{Ce}^{WA}$ in Figure S10). This lowering in the barrier (44.3 > 41.5 > 37.0 kcal/mol) with an increase in the coordination number of Ce was due to the following factors. ${R^{\prime \prime }}_{Ce}^{WA}$ possessed the longest Ce-O^H^ distance (2.05 Å) followed by ${R^{\prime }}_{Ce}^{WA}$ (1.99 Å) and ${\text{R}}_{Ce}^{WA}$ (1.98 Å), Table S3. Additionally, the charge on the Ce atom followed the order ${\text{R}}_{Ce}^{WA}$ > ${R^{\prime }}_{Ce}^{WA}$ > ${R^{\prime \prime }}_{Ce}^{WA}$ (Table S4). These differences indicated that the basicity of the metal-bound hydroxide in ${R^{\prime \prime }}_{Ce}^{WA}$ was greater than its basicity in ${R^{\prime }}_{Ce}^{WA}\;$and ${\text{R}}_{Ce}^{WA}$.

The reactivity of **Ce-C** was enhanced with an increase in the coordination number (7-9) of the Ce atom. The ***CA***mechanism was substantially less favorable, and the ***WA***and ***DA*** mechanisms were quiet comparable for **Ce-C**. Based on these results, the ***CA***mechanism was not explored for **Zr-C** and **Ti-C** in the next section.

#### Direct Attack (DA) Mechanism for the Zr-C and Ti-C Complexes

Zr and Ti have been reported to prefer different coordination numbers i.e., 8 and 7, respectively (Luong et al., 2016; Zhang et al., 2016a; Assi et al., 2017). In the reactant (**R**_**Zr**_) with coordination number 8, an external water molecule was directly coordinated to the Zr ion (Figure S11). In comparison to **R'**_**Ce**_ (the reactant of **Ce-C** with the same coordination number), all metal-ligand distances, except P-O^4^, were substantially shorter in **R**_**Zr**_ (Tables S3, S5). From **R**_**Zr**_, the Zr bound -O^H^H^H^ nucleophile attacked BNPP with a barrier of 40.3 kcal/mol, which was 2.6 kcal/mol higher than the one (37.7 kcal/mol) computed for **Ce-C**. A weaker nucleophile (shorter Zr-O^H^ distance by 0.05 Å) and increase in the charge of Zr (by 0.15*e*) raised the barrier for this step. The intermediate (${\text{I}}_{Zr}^{DA}$) formed in this step was endergonic by 28.3 kcal/mol from **R**_**Zr**_. However, the P-O^4^ bond in ${\text{I}}_{Zr}^{DA}$ was substantially stronger (by 0.12 Å) than in the **Ce-C** case (Figures S11, S6). Due to the extra stability of this bond, unlike the mechanism for **Ce-C**, a complete cleavage of this bond required the assistance of metal-bound water in the next step. From ${\text{I}}_{Zr}^{DA}$, the Zr-bound water donated a proton to the O^4^ atom and cleaved the P-O^4^ bond. The splitting of this bond occurred with a barrier of 8.8 kcal/mol from ${\text{I}}_{Zr}^{DA}$ i.e., 37.1 kcal/mol from **R**_**Zr**_ (Figure S11). In the product (${\text{P}}_{Zr}^{DA}$), the neutral nitrophenol group was hydrogen bonded to the Zr-hydroxyl moiety and it was exergonic by 9.9 kcal/mol. The removal of a water ligand from this complex (coordination number of Zr = 7) raised the barrier for the rate-limiting first step by 2.4 kcal/mol (Figure S12).

Since Ti prefers coordination number 7, the geometry of the reactant (**R**_**Ti**_) of **Ti-C** was different from the reactant of **Zr-C**. In **R**_**Ti**_ (Figure S13), an external water molecule, instead of directly coordinating to the metal ion, was bridged through hydrogen bonding between the cyclen ring and BNPP. All metal-ligand distances in **R**_**Ti**_ were substantially shorter than the corresponding distances in **R**_**Zr**_ (Table S5), while the P-O^4^ bond distance (P-O^4^ = 1.57 Å) remained unchanged. From **R**_**Ti**_, the nucleophilic attack took place with a barrier of 39.0 kcal/mol. This barrier was slightly (1.3 kcal/mol) lower than the one computed for **Zr-C**. The intermediate (${\text{I}}_{Ti}^{DA}$) formed in this step was endergonic by 24.9 kcal/mol from **R**_**Ti**_. The cleavage of the P-O^4^ bond using the Ti-bound water molecule took place with a barrier of 11.5 kcal/mol from ${\text{I}}_{Ti}^{DA}$ i.e., 36.4 kcal/mol from **R**_**Ti**_ (Figure S13). The product (${\text{P}}_{Ti}^{DA}$) in which the neutral nitrophenol group was associated with the metal-bound hydroxyl through hydrogen bonding was exergonic by 10.7 kcal/mol. The inclusion of a water ligand to this complex (coordination number of Ti = 8) raised the barrier for the rate-limiting step by 9.0 kcal/mol i.e., 48.0 kcal/mol from the corresponding reactant (Figure S14).

**Zr-C** and **Ti-C** showed higher activity with different coordination numbers i.e., 8 and 7 for Zr and Ti, respectively. They also required assistance of an external water, unlike **Ce-C**, for the complete cleavage of the P-O bond. However, both **Zr-C** (coordination number = 8) and **Ti-C** (coordination number = 7) were found to be less active than **Ce-C** (with coordination number 9) for the ***DA*** mechanism (Table 1).

**Table 1:** Computed energy barrier in the rate-limiting step for all **M-C** complexes.

**Complex**	**Coordination number**	**Mechanism**	**Barrier (kcal/mol)**
Zn-C	6	DA	20.7
		CA	23.5
		WA	20.9
Cu-C	6	DA	22.0
		CA	24.3
		WA	24.2
Co-C	6	DA	23.4
		CA	24.6
		WA	24.4
Ce-C	7	DA	39.1
Ce-C (+1w)	8	DA	37.7
Ce-C (+2w)	9	DA	36.2
Ce-C	7	CA	46.8
Ce-C (+1w)	8	CA	44.5
Ce-C (+2w)	9	CA	44.0
Ce-C	7	WA	44.3
Ce-C (+1w)	8	WA	41.5
Ce-C (+2w)	9	WA	37.0
Zr-C	7	DA	42.7
Zr-C (+1w)	8	DA	40.3
Zr-C	7	WA	37.0
Zr-C (+1w)	8	WA	36.2
Ti-C	7	DA	39.0
Ti-C (+1w)	8	DA	48.0
Ti-C	7	WA	32.7
Ti-C (+1w)	8	WA	34.1

#### Water-Assisted (WA) Mechanism for the Zr-C and Ti-C Complexes

In the reactant (${\text{R}}_{Zr}^{WA}$) of **Zr-C**, an external water molecule was hydrogen bonded between the Zr-O^H^H^H^ and BNPP (Figure S15). In this mechanism, the Zr-bound hydroxyl functioned as a base and created a hydroxyl (-O^1w^H^1w^) nucleophile from the external water molecule that concomitantly attacked the electrophilic P atom of BNPP. This synchronous step took place with a barrier of 28.8 kcal/mol (Figure S15). The barrier for this step was significantly (6.0 kcal/mol) lower than the barrier for **Ce-C**. The intermediate (${\text{I}1}_{Zr}^{WA}$) formed in this step was 22.3 kcal/mol endergonic from ${\text{R}}_{Zr}^{WA}$. As observed for the previous ***DA***mechanism, the scissile P-O^4^ bond in ${\text{I}1}_{Zr}^{WA}$ was activated but still quite strong (1.62 Å) i.e., 0.13 Å stronger than for **Ce-C**. The complete cleavage of this bond also needed the assistance of a metal-bound water molecule in the next step. The ${\text{I}1}_{Zr}^{WA}$ intermediate reoriented itself and created another 3.0 kcal/mol endergonic intermediate (${\text{I}2}_{Zr}^{WA}$) in which the water molecule was located in a position to protonate the O^4^ atom of BNPP. From ${\text{I}2}_{Zr}^{WA}$, this water molecule donated its proton and cleaved the P-O^4^ bond with a barrier of 10.9 kcal/mol. The final product (${\text{P}}_{Zr}^{WA}$) was 33.6 kcal/mol exergonic from ${\text{R}}_{Zr}^{WA}$(Figure S15). The removal of a water molecule in this complex (coordination number of Zr = 7) slightly raised the rate-limiting barrier by 0.8 kcal/mol (Figure S16).

The reactant (${\text{R}}_{Ti}^{WA}$) of **Ti-C** (for coordination number of Ti = 7) was structurally similar to ${\text{R}}_{Zr}^{WA}$ (Figure S17). However, all metal-ligand distances in the former were shorter than the corresponding distances in the latter (Table S5). From ${\text{R}}_{Ti}^{WA}$, proton abstraction from the external water molecule occurred with a barrier of 32.7 kcal/mol (Figure S17). The barrier in this nucleophilic attack step was 3.9 kcal/mol higher than the one computed for **Zr-C**. As discussed previously, this increase was due to a shorter M-O^H^ bond distance (by 0.19 Å) and lower charge on the O^H^ atom (by 0.22*e*) in ${\text{R}}_{Ti}^{WA}$ (Tables S5, S6). However, the phosphorane intermediate (${\text{I}1}_{Ti}^{WA}$) in this step was 7.2 kcal/mol more favorable than in the **Zr-C** case. i.e., 15.1 kcal/mol endergonic from ${\text{R}}_{Ti}^{WA}$. The presence of a stronger hydrogen bond provided extra stability to this complex. Similar to **Zr-C**, the newly formed Ti-bound water molecule in ${\text{I}1}_{Ti}^{WA}$ reoriented itself between the cyclen ring and BNPP to create another intermediate ${\text{I}2}_{Ti}^{WA}$. This intermediate was 5.7 kcal/mol higher in energy than ${\text{I}1}_{Ti}^{WA}$. From ${\text{I}2}_{Ti}^{WA}$, the transfer of the H^1w^ proton to the O^4^ atom led to the splitting of the P-O^4^ bond. This process took place with a barrier of 11.5 kcal/mol from ${\text{I}2}_{Ti}^{WA}$. In the product (${\text{P}}_{Ti}^{WA}$), the released nitrophenol group was hydrogen bonded to the metal-bound hydroxyl and it was 14.8 kcal/mol exergonic from ${\text{R}}_{Ti}^{WA}$. The addition of a water molecule in this complex (coordination number of Ti = 8) slightly increased the barrier in the rate-limiting step by 1.4 kcal/mol (Figure S18).

These results suggest that the ***WA***mechanism was energetically more favorable than the ***DA***mechanism for both **Zr-C** and **Ti-C**. Among these two complexes, **Ti-C** was found to be more reactive than **Zr-C**.

## Conclusions

**Figure 5:**
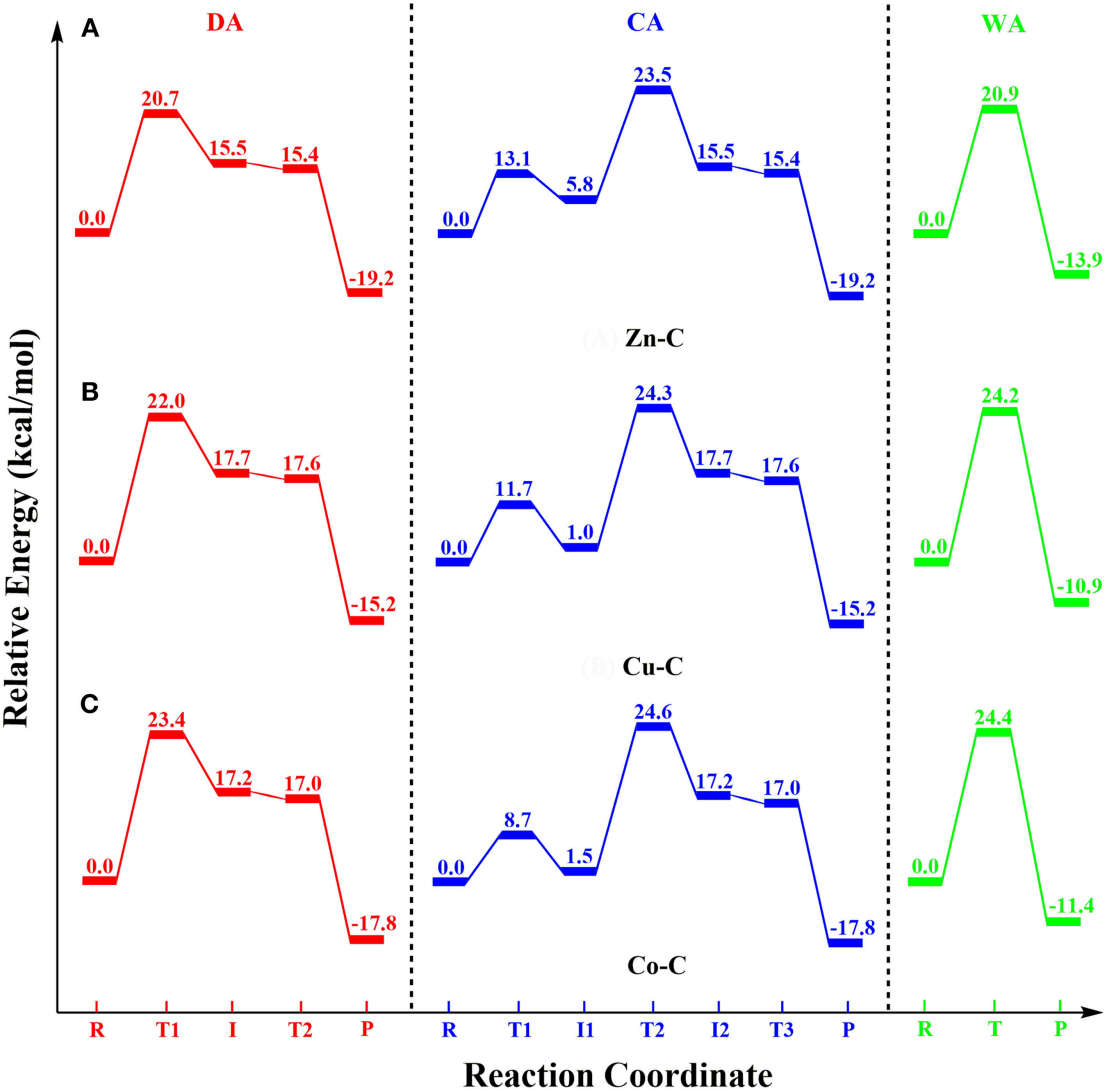
Potential energy surface diagrams for the divalent **M-C** complexes: **(A) Zn-C**, **(B) Cu-C** and **(C) Co-C**.

**Figure 6:**
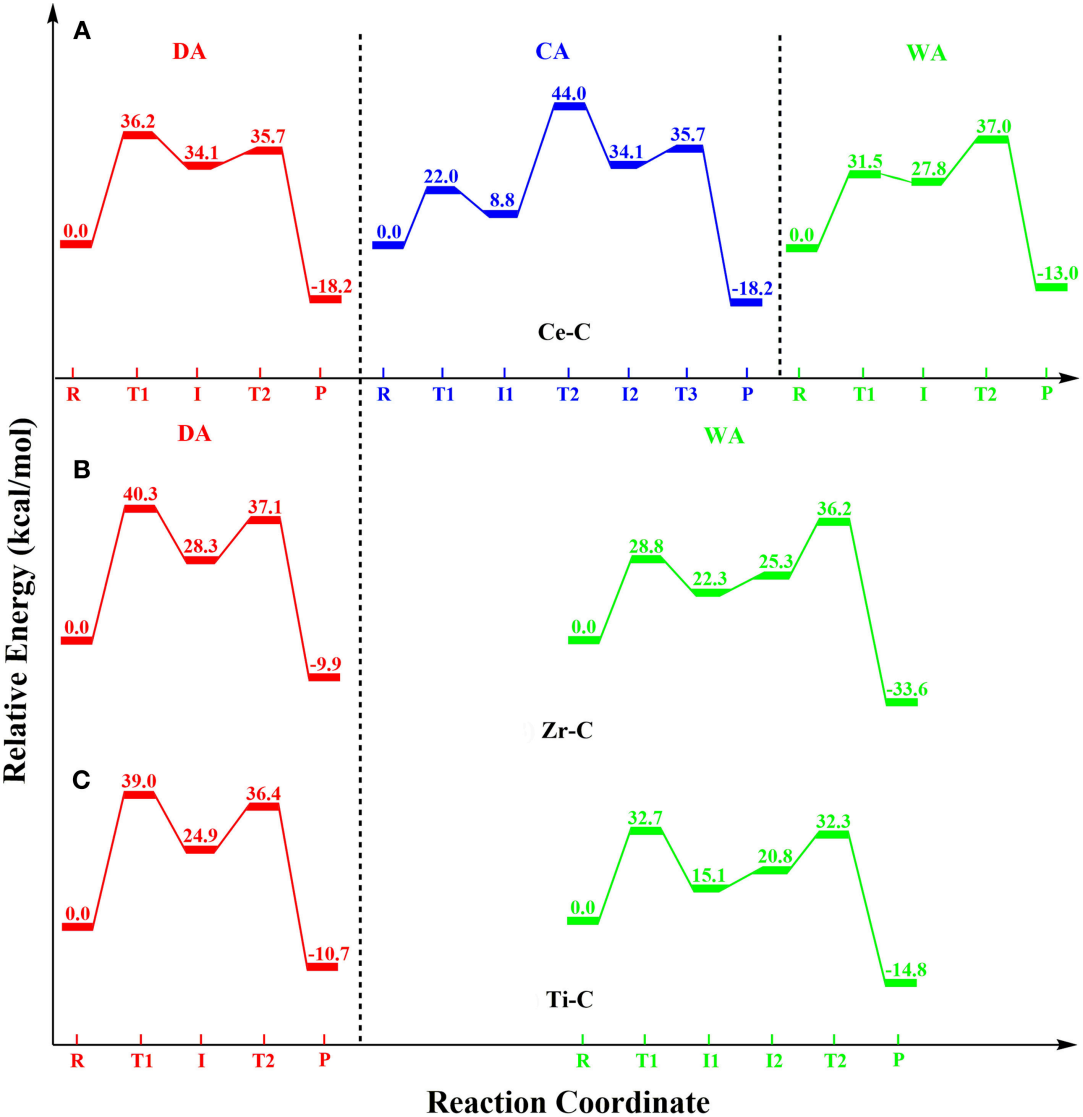
Potential energy surface diagrams for the tetravalent **M-C** complexes: **(A) Ce-C** (coordination number = 9), **(B) Zr-C** (coordination number = 8), and **(C) Ti-C** (coordination number = 7).

In this DFT study, phosphodiester hydrolysis by metal-cyclen (**M-C**) complexes using both divalent [Zn(II), Cu(II) and Co(II)] and tetravalent [Ce(IV), Zr(IV), and Ti(IV)] metals were investigated. The reactivities of all six **M-C** complexes (**Zn-C, Cu-C, Co-C, Ce-C, Zr-C** and **Ti-C**) for BNPP hydrolysis were studied using three different mechanisms: (1) direct attack (***DA***), (2) catalyst-assisted (***CA***), and (3) water-assisted (***WA***). Their energetics were compared using the metal-ligand, metal-nucleophile and P-O bond lengths, strain of the cyclen ring, atomic charges and coordination number of metal ions as parameters. The potential energy surface diagrams (PES) of all these mechanisms for the divalent and tetravalent complexes are shown in Figures 5, 6, respectively.

For all divalent metal complexes (**Zn-C, Cu-C**, and **Co-C**), the binding of the BNPP substrate in the monodentate fashion activated its scissile phosphoester bond (P-O^4^) by ~0.04 Å. Their energetics were controlled by distinct chemical factors: nucleophilicity of the metal center in the ***DA***mechanism; basicity of the N^1^ atom of the cyclen ring in the ***CA***mechanism; and basicity of the metal bound hydroxyl group in the ***WA***mechanism. The ***DA***mechanism was found to be energetically most favorable for all these complexes. Among the divalent complexes, **Zn-C** was more reactive than **Cu-C** and **Co-C** for all three mechanisms (Figure 5, Table 1).

On the other hand, the binding of BNPP to the tetravalent metal complexes (**Ce-C**, **Zr-C**, and **Ti-C**) in the bidentate manner strengthened its P-O^4^ bond by ~0.03 Å. The computed barriers for these complexes were substantially higher than the barriers for their divalent counterparts for all three mechanisms (Table 1). Unlike the ***DA*** mechanism for divalent **M-C** complexes, the ***WA***mechanism was energetically most favorable for **Zr-C** and **Ti-C**. On the other hand, energetics of both ***DA***and ***WA*** mechanisms were comparable for **Ce-C**. The activities of **Ce-C** and **Zr-C** improved with an increase in the coordination number (7-9) of the metal ion for all three mechanisms, while **Ti-C** exhibited the opposite trend (Table 1). In comparison to **Ce-C**, both **Zr-C** and **Ti-C** required additional assistance for the complete cleavage of the P-O^4^ bond. **Ce-C** exhibited the highest activity with a coordination number of Ce = 9, **Zr-C** with a coordination number of Zr = 8 and **Ti-C** with a coordination number of Ti = 7. However, among all tetravalent complexes, **Ti-C** was found to be the most reactive (barrier = 32.7 kcal/mol using the ***WA***mechanism) followed by **Ce-C** and **Zr-C** (Figure 6, Table 1).

These results have provided detailed structural, mechanistic and kinetic information regarding the activities of a wide range of **M-C** complexes. They will pave the way for the design of efficient synthetic metallohydrolases for applications in biology, biotechnology and medicine.

## Author Contributions

QH performed most of the DFT calculations and analyzed them. He also wrote the first draft of the manuscript and made figures and tables. VJ-A performed some DFT calculations and analyzed them. She also helped with the writing of the draft. JZ started the project and performed initial DFT calculations. RP designed and supervised the project. He also analyzed the results and edited the manuscript.

### Conflict of Interest Statement

The authors declare that the research was conducted in the absence of any commercial or financial relationships that could be construed as a potential conflict of interest.
