# Supplementary material for: Effects of the Metal Ion on the Mechanism of Phosphodiester Hydrolysis Catalyzed by Metal-Cyclen Complexes
Source: Front Chem. 2019 Apr 5;7:195. doi: 10.3389/fchem.2019.00195 (PMC6460053; doi:10.3389/fchem.2019.00195)
Supplement: Supplementary file 1 [file Data_Sheet_1.pdf]

Effects of the Metal Ion on the Mechanism of Phosphodiester Hydrolysis Catalyzed by Metal-Cyclen Complexes

Qiaoyu Hu, Vindi M. Jayasinghe-Arachchige, Joshua Zuchniarz and Rajeev Prabhakar*

Department of Chemistry, University of Miami, Coral Gables, FL 33146

* To whom correspondence should be addressed; rpr@miami.edu; Tel: 305-284-9372;

Fax: 305-284-4571.

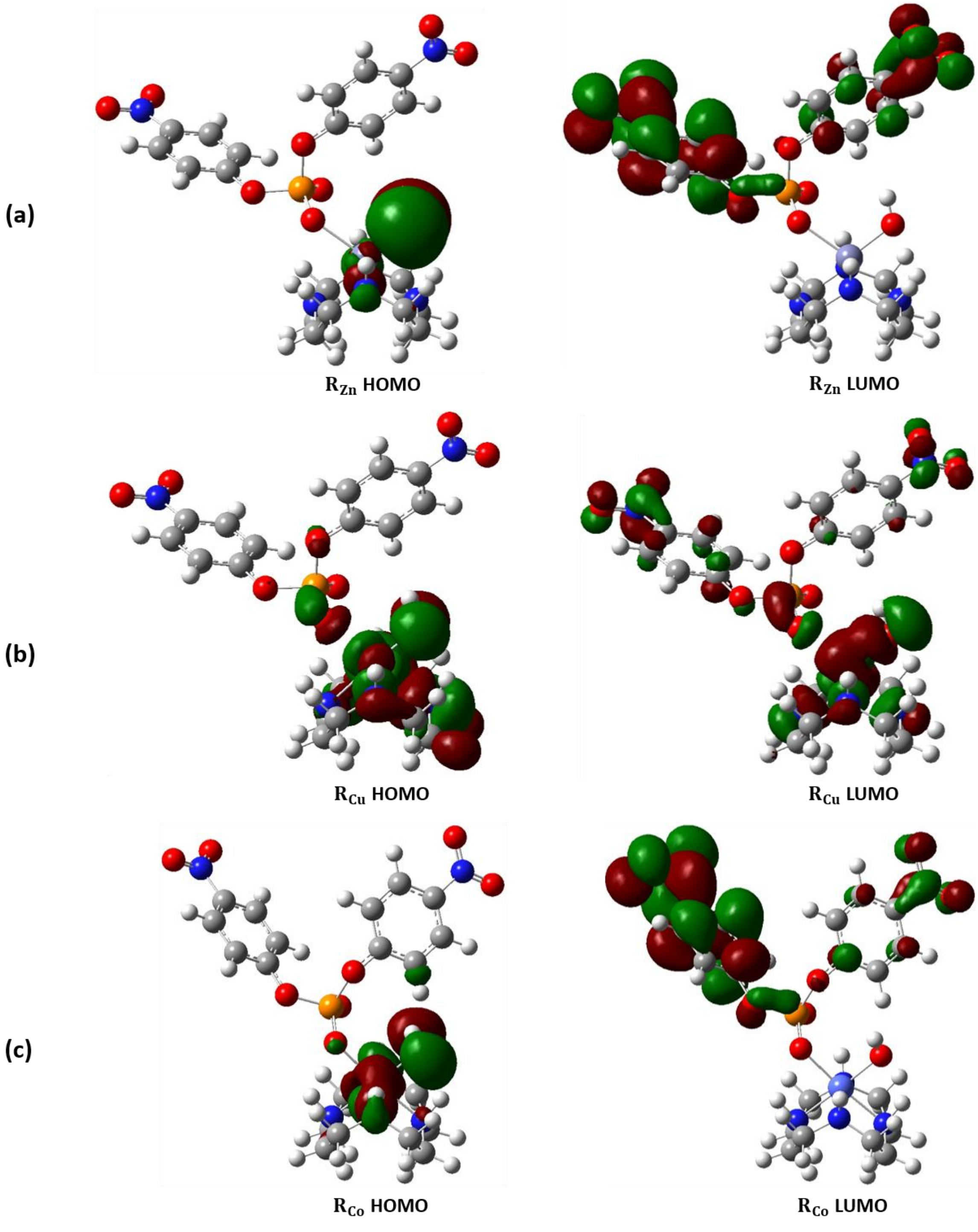

Figure S1. Frontier molecular orbitals (HOMO, LUMO) for (**a**) $\mathbf{R_{Zn}}$, (**b**) $\mathbf{R_{Cu}}$, and (**c**) $\mathbf{R_{Co}}$.

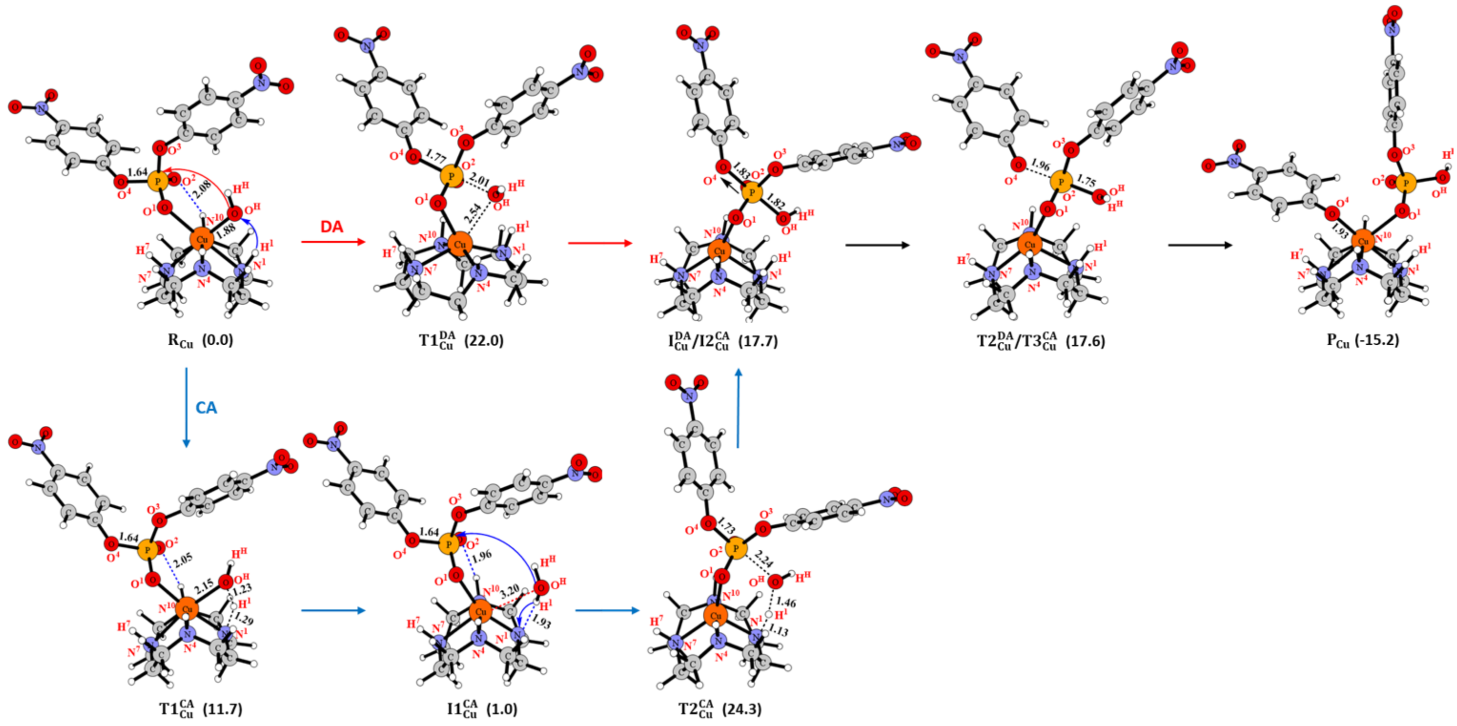

Figure S2. Structures (in Å) and energies (in kcal/mol) in the ***DA*** and ***CA*** mechanisms for **Cu-C**.

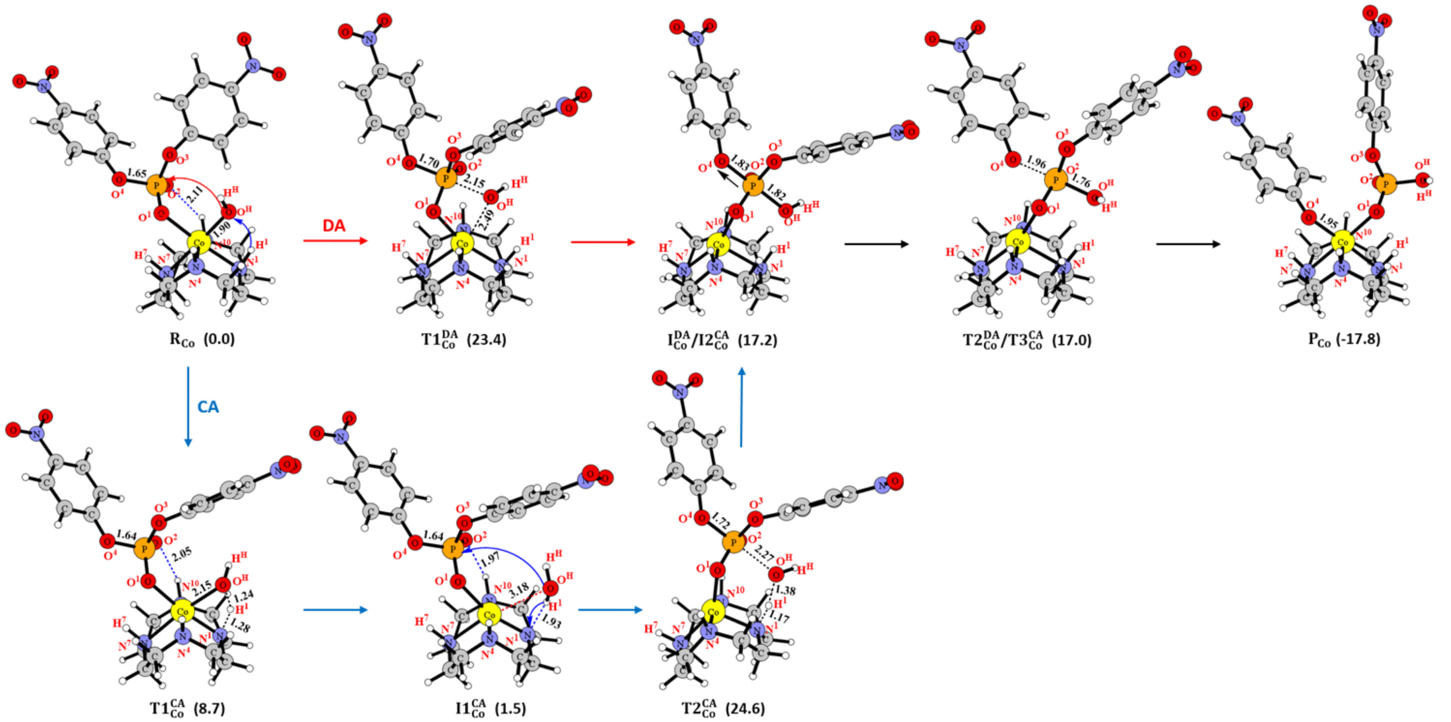

Figure S3. Structures (in Å) and energies (in kcal/mol) in the ***DA*** and ***CA*** mechanisms for **Co-C**.

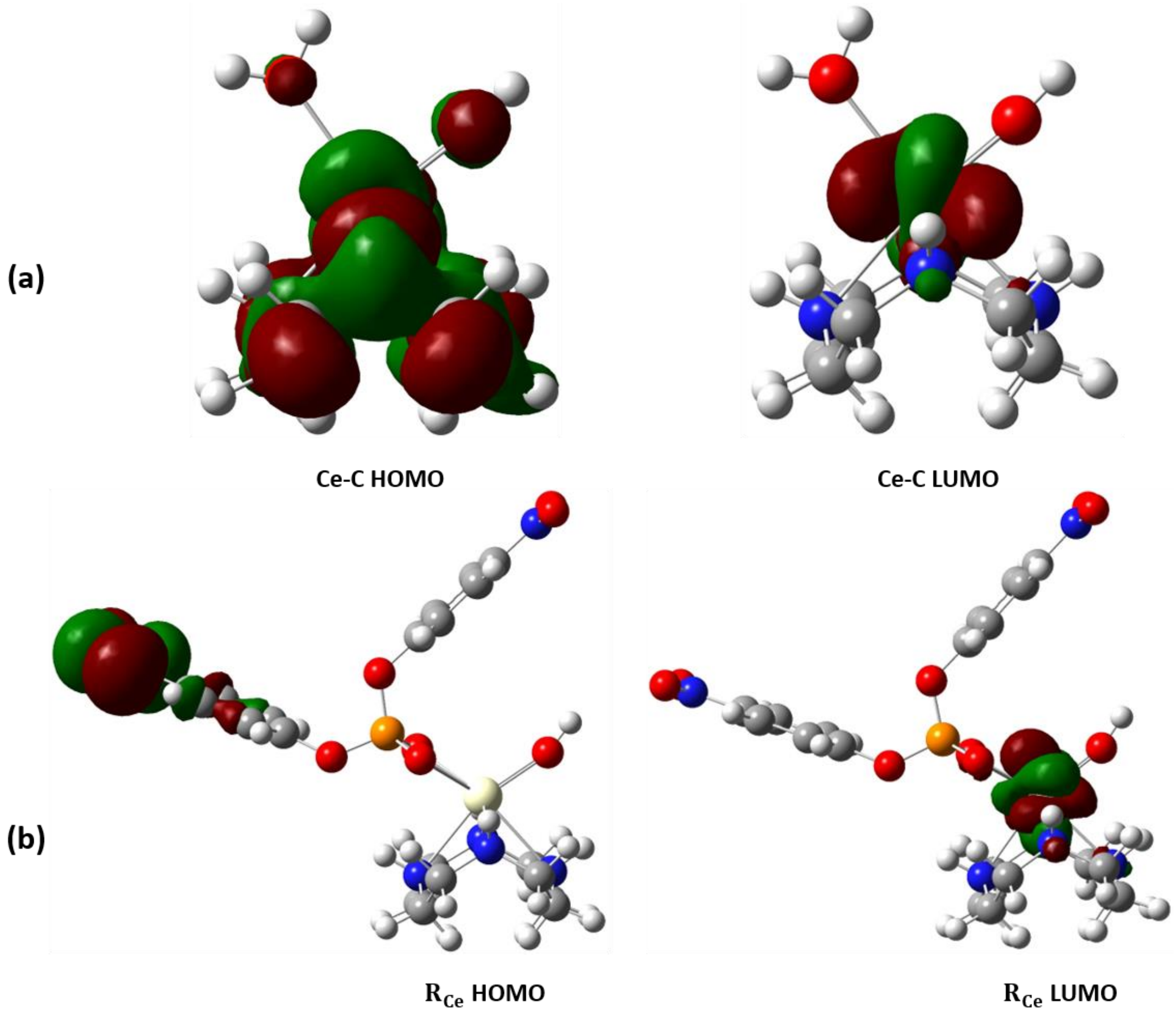

Figure S4. Frontier molecular orbitals for (**a**) **Ce-C** without BNPP and (**b**) **Ce-C** with BNPP ($\mathbf{R_{Ce}}$).

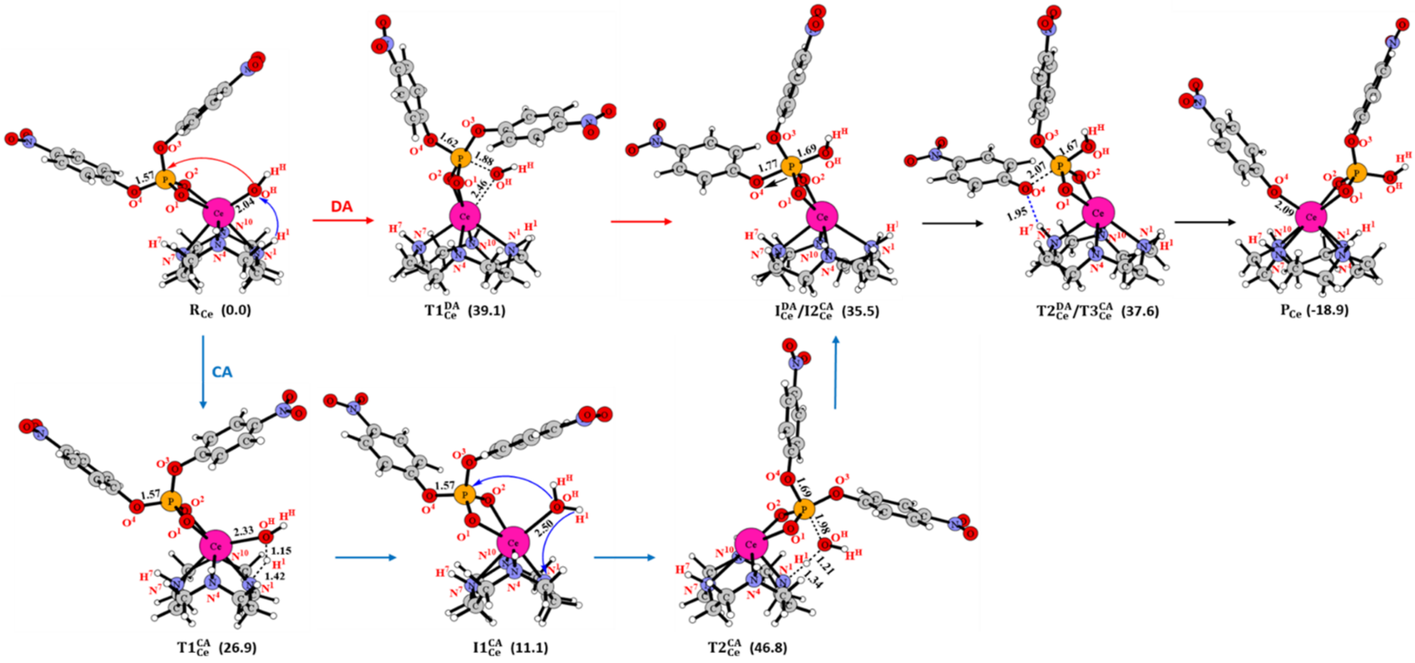

Figure S5. Structures (in Å) and energies (in kcal/mol) in the ***DA*** and ***CA*** mechanism for **Ce-C** (coordination number = 7).

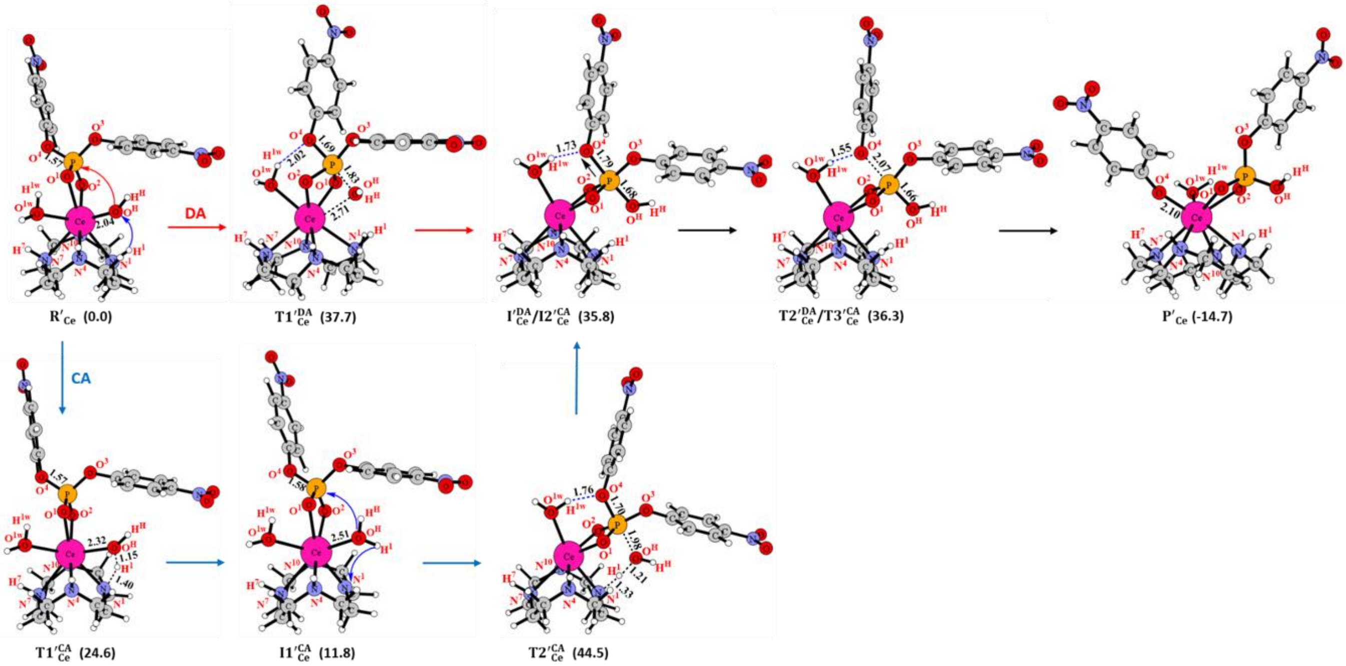

Figure S6. Structures (in Å) and energies (in kcal/mol) in the ***DA*** and ***CA*** mechanisms for **Ce-C** (coordination number = 8).

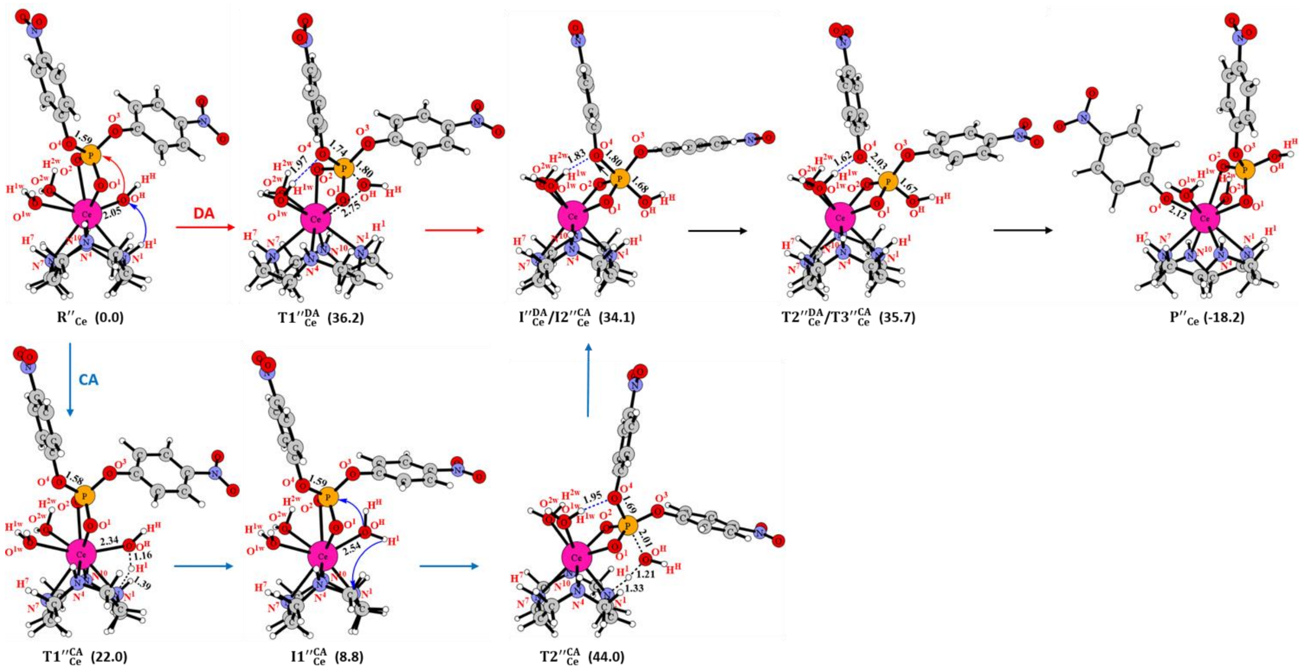

Figure S7. Structures (in Å) and energies (in kcal/mol) in the ***DA*** and ***CA*** mechanisms for **Ce-C** (coordination number = 9).

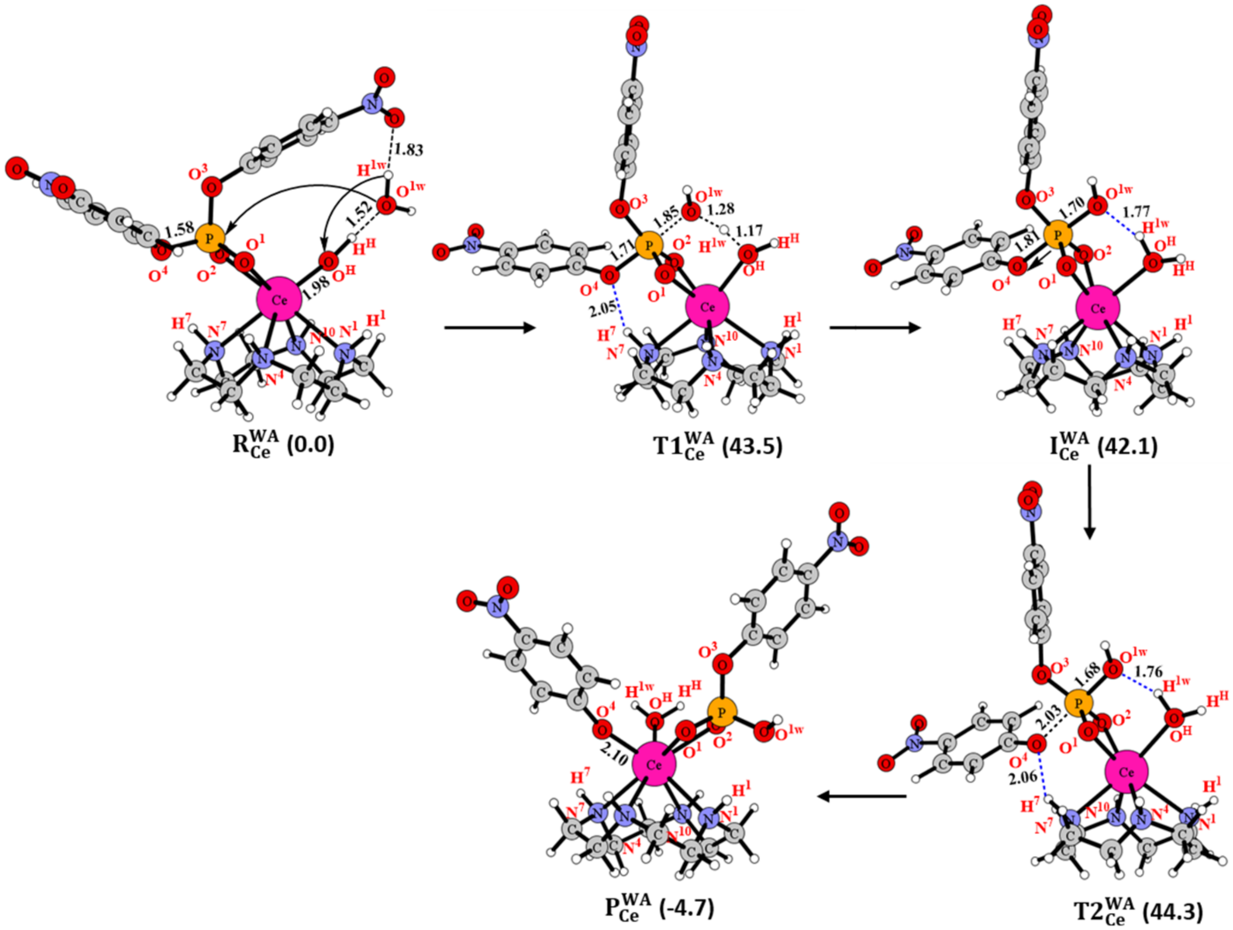

Figure S8. Structures (in Å) and energies (in kcal/mol) in the ***WA*** mechanism for **Ce-C** (coordination number = 7).

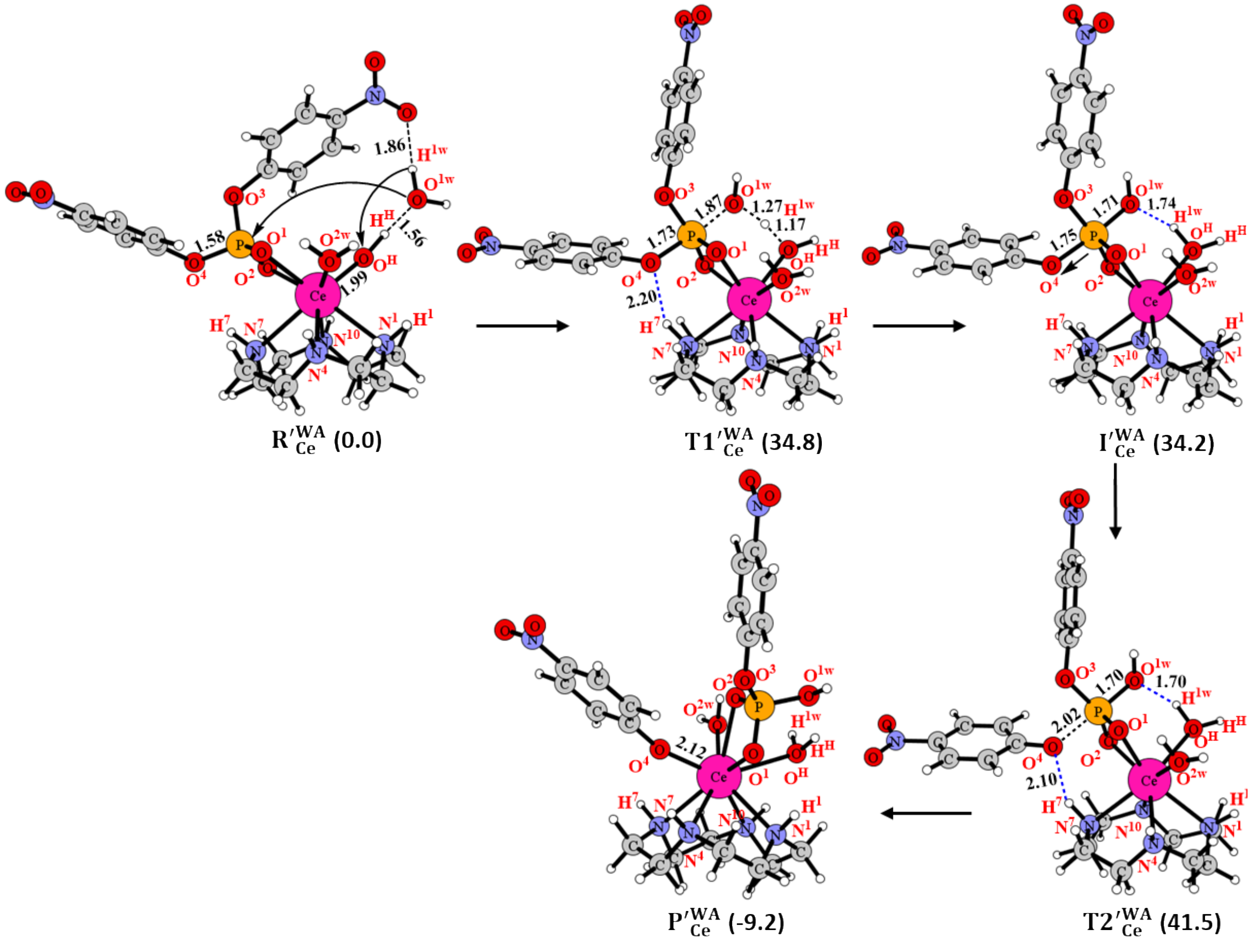

Figure S9. Structures (in Å) and energies (in kcal/mol) in the ***WA*** mechanism for **Ce-C** (coordination number = 8).

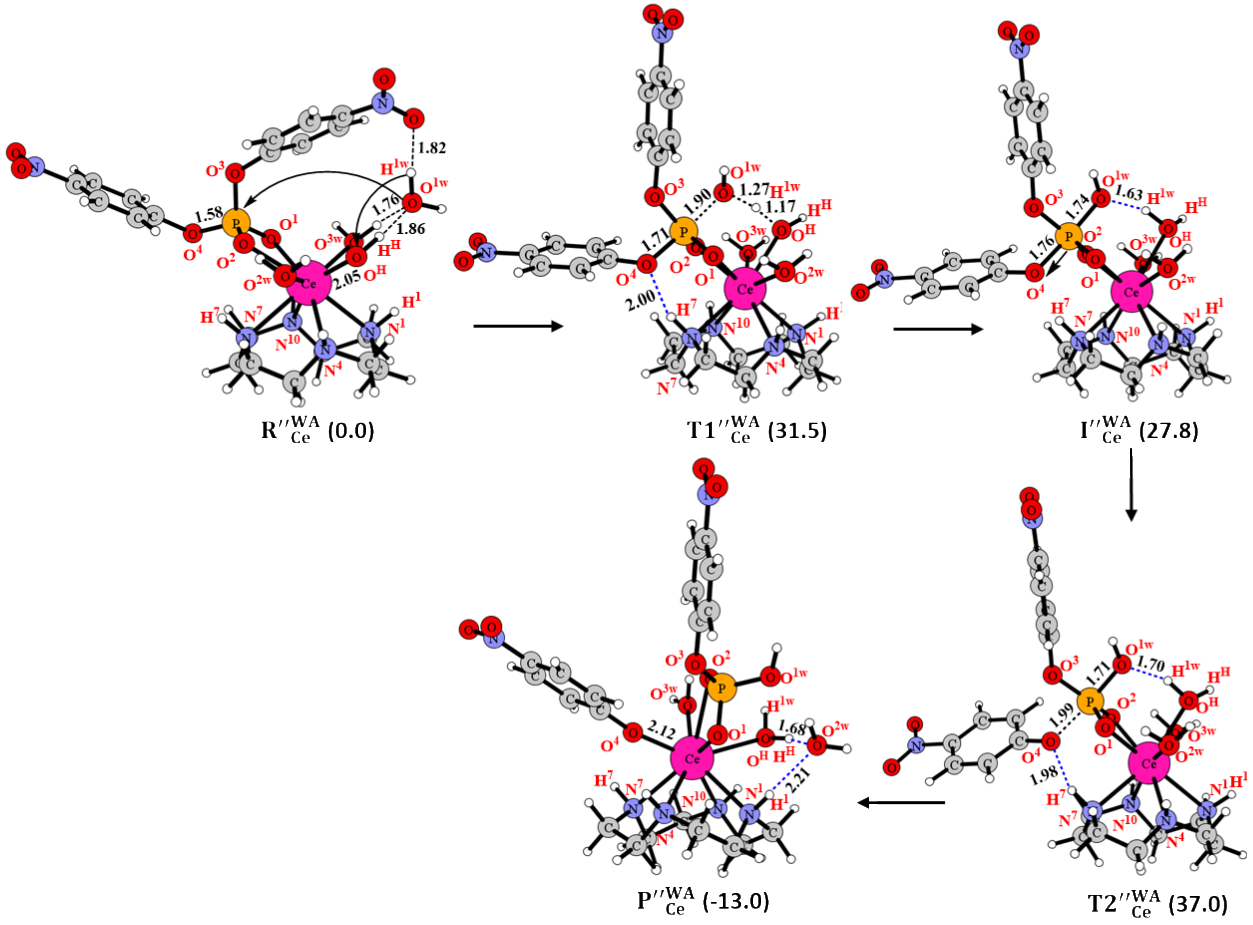

Figure S10. Structures (in Å) and energies (in kcal/mol) in the ***WA*** mechanism for **Ce-C** (coordination number = 9).

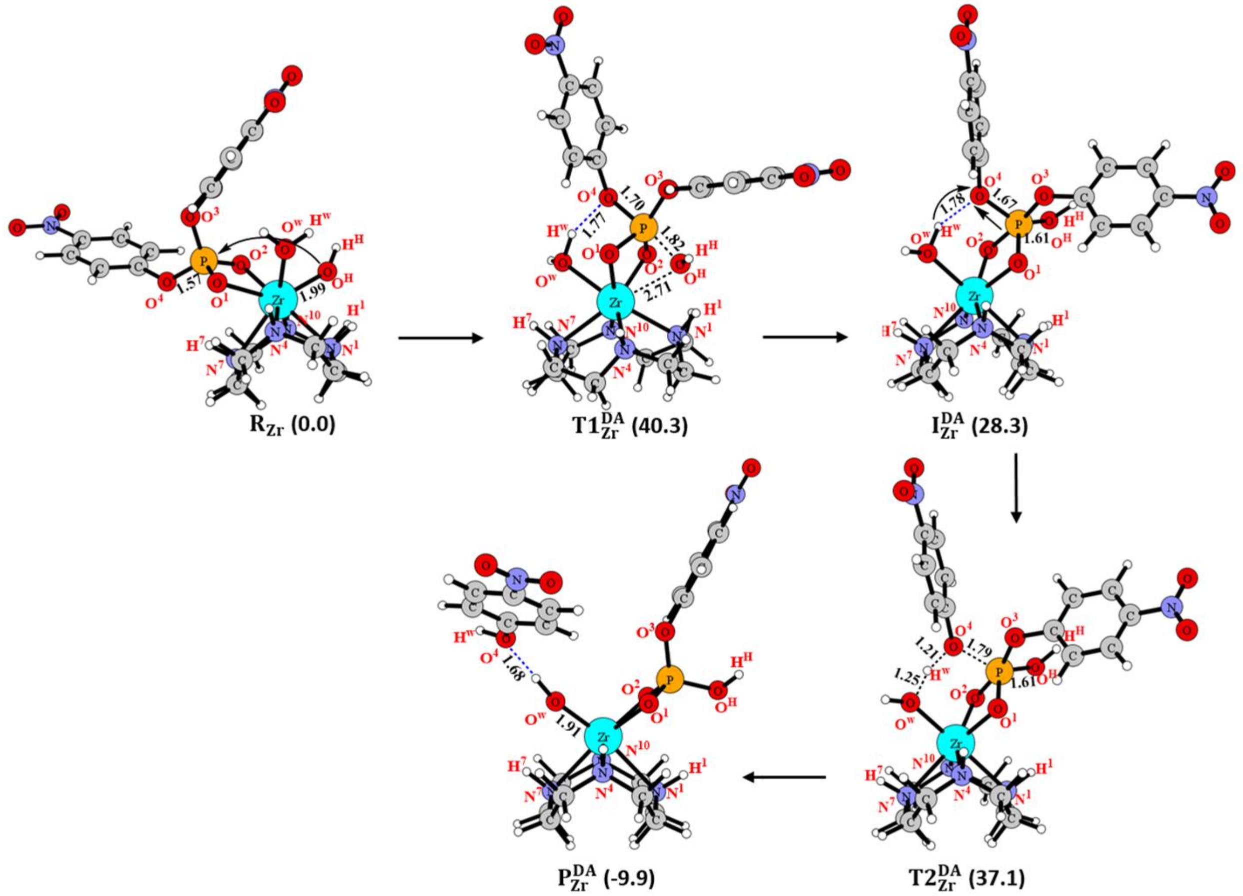

Figure S11. Structures (in Å) and energies (in kcal/mol) in the ***DA*** mechanism for **Zr-C** (coordination number = 8).

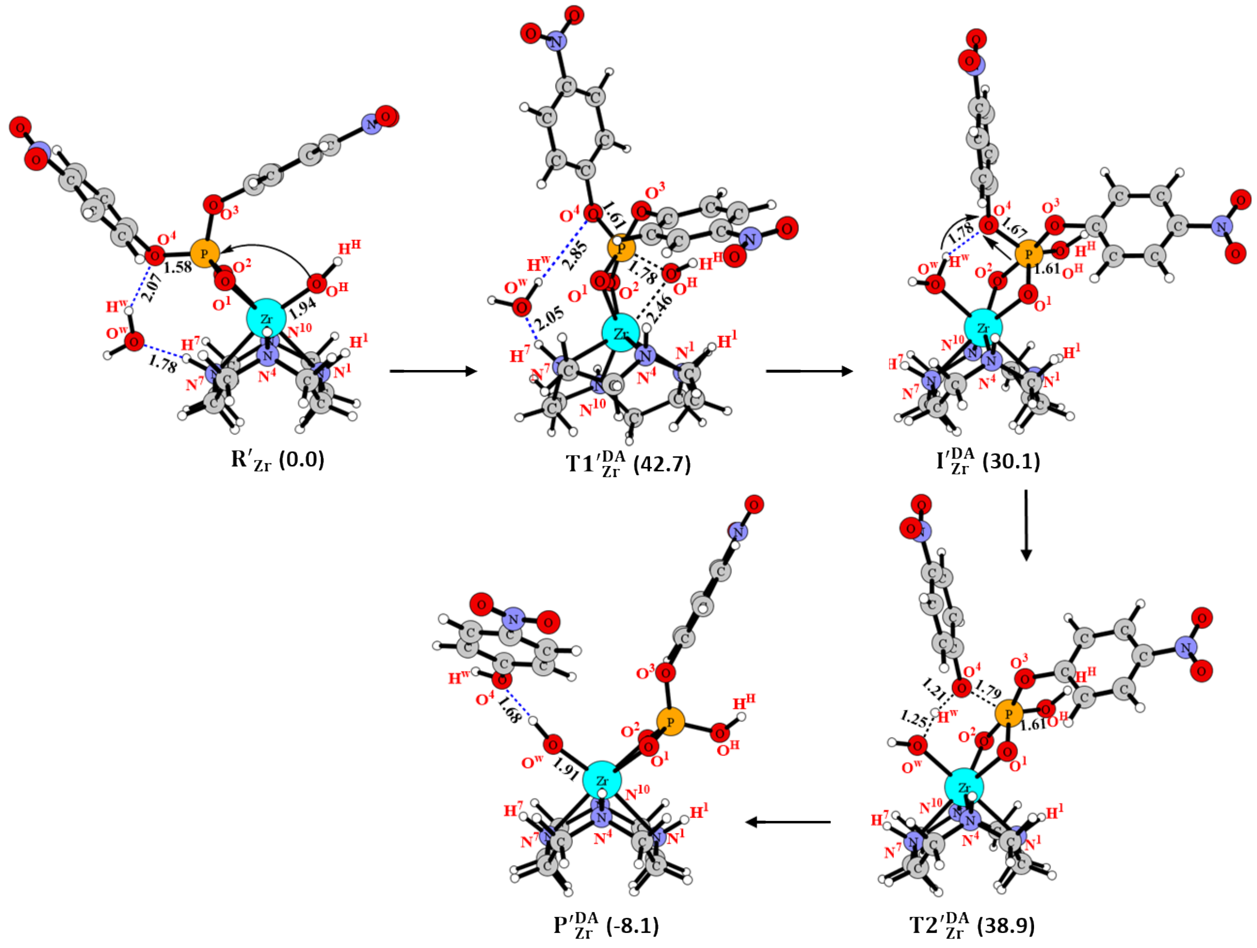

Figure S12. Structures (in Å) and energies (in kcal/mol) in the ***DA*** mechanism for **Zr-C** (coordination number = 7).

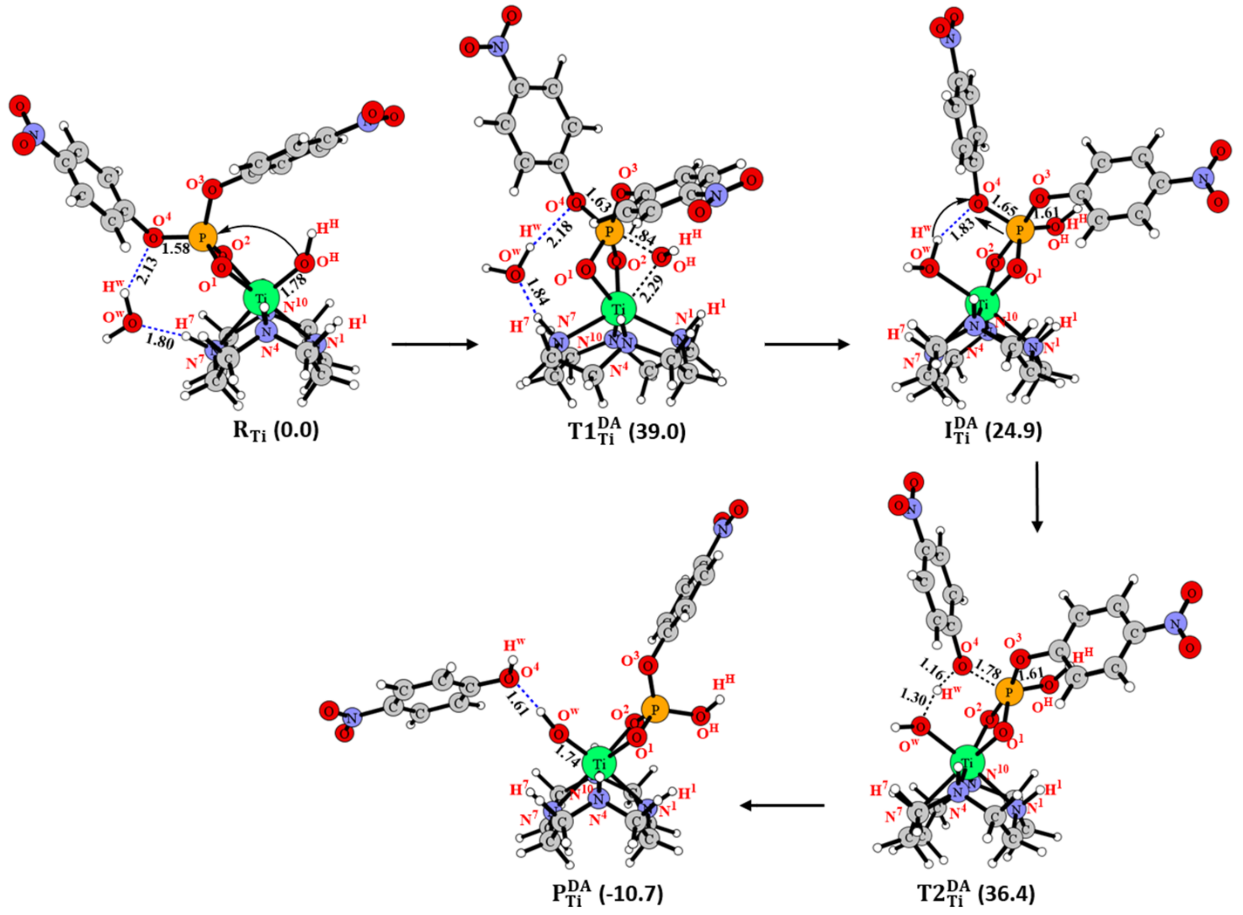

Figure S13. Structures (in Å) and energies (in kcal/mol) in the ***DA*** mechanism for **Ti-C** (coordination number = 7).

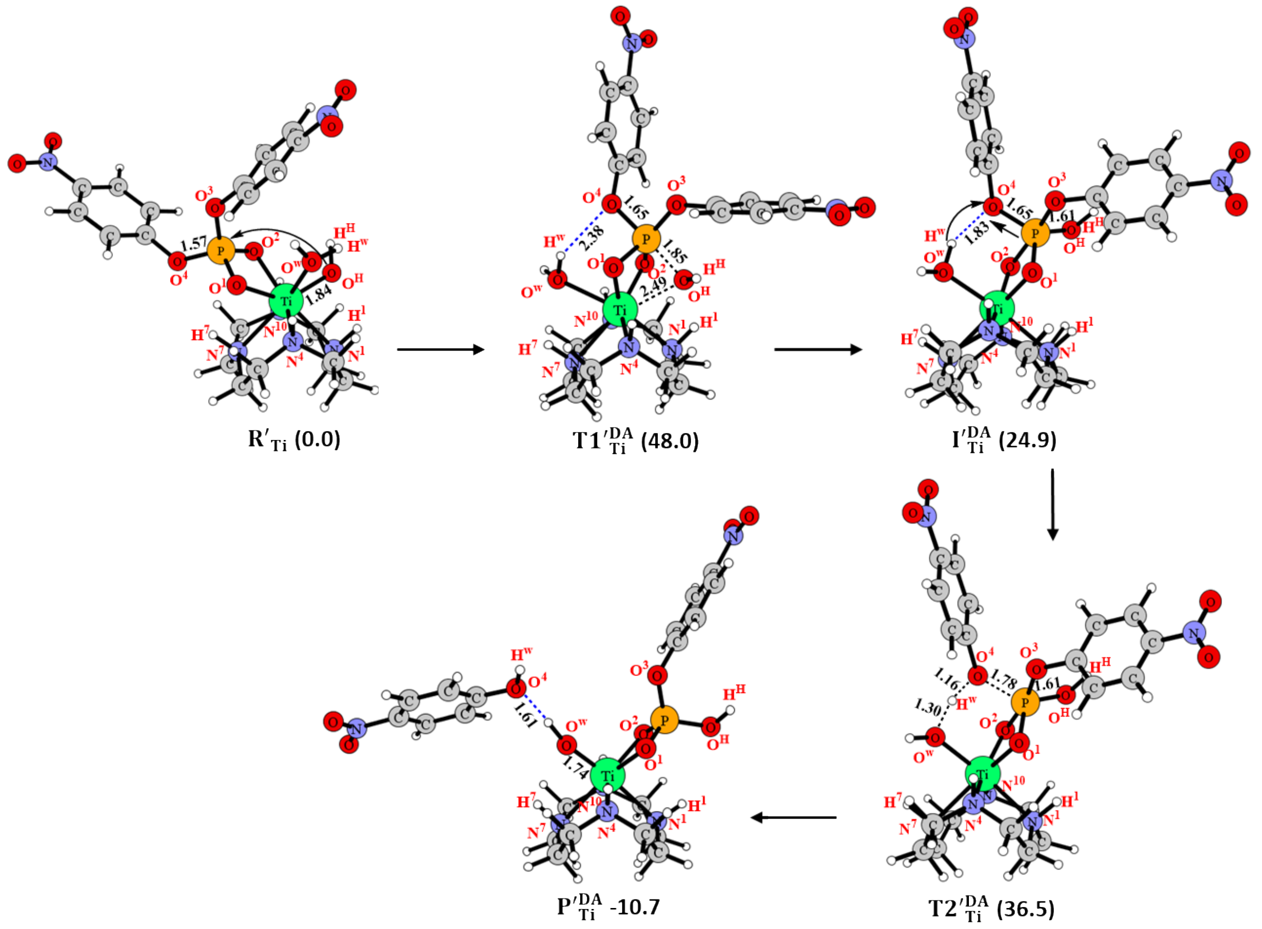

Figure S14. Structures (in Å) and energies (in kcal/mol) in the ***DA*** mechanism for **Ti-C** (coordination number = 8).

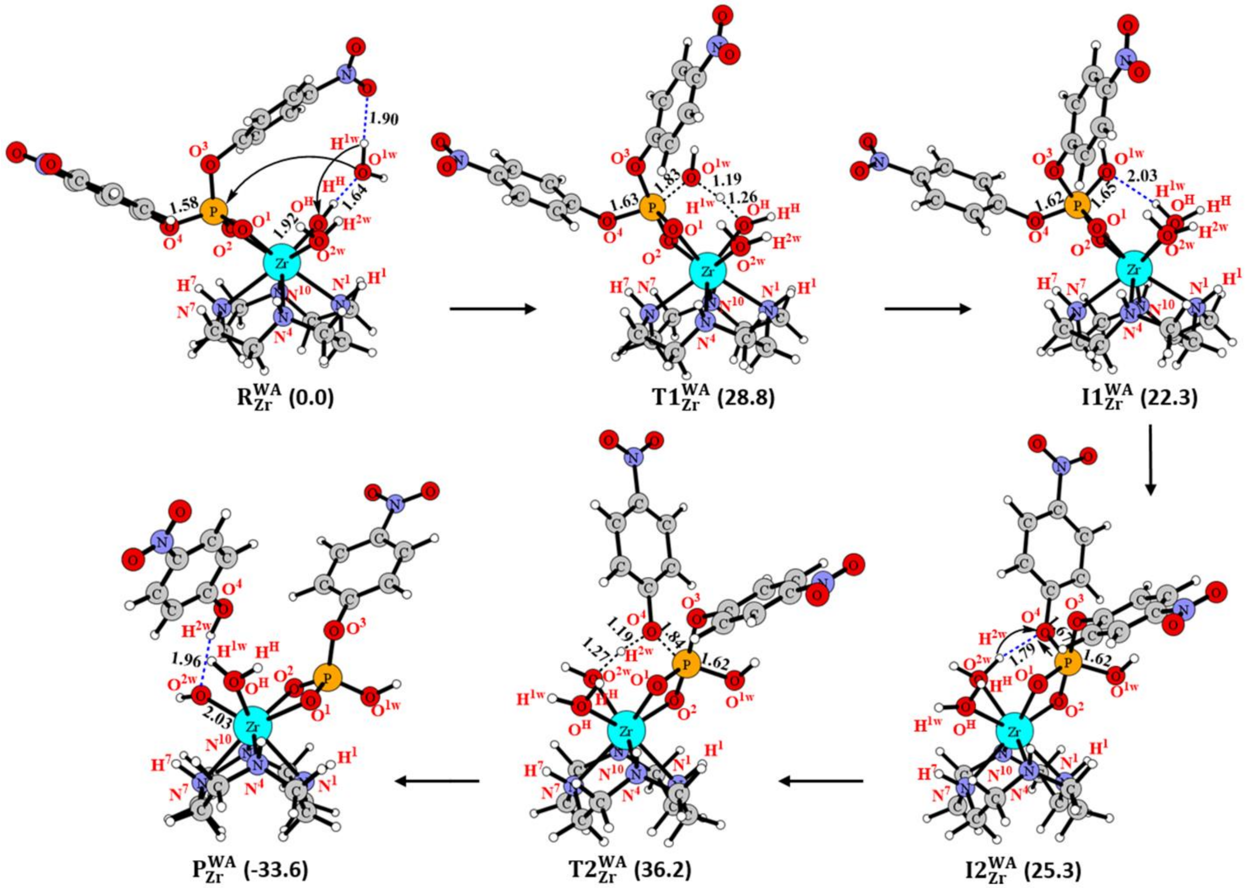

Figure S15. Structures (in Å) and energies (in kcal/mol) in the **WA** mechanism for **Zr-C** (coordination number = 8).

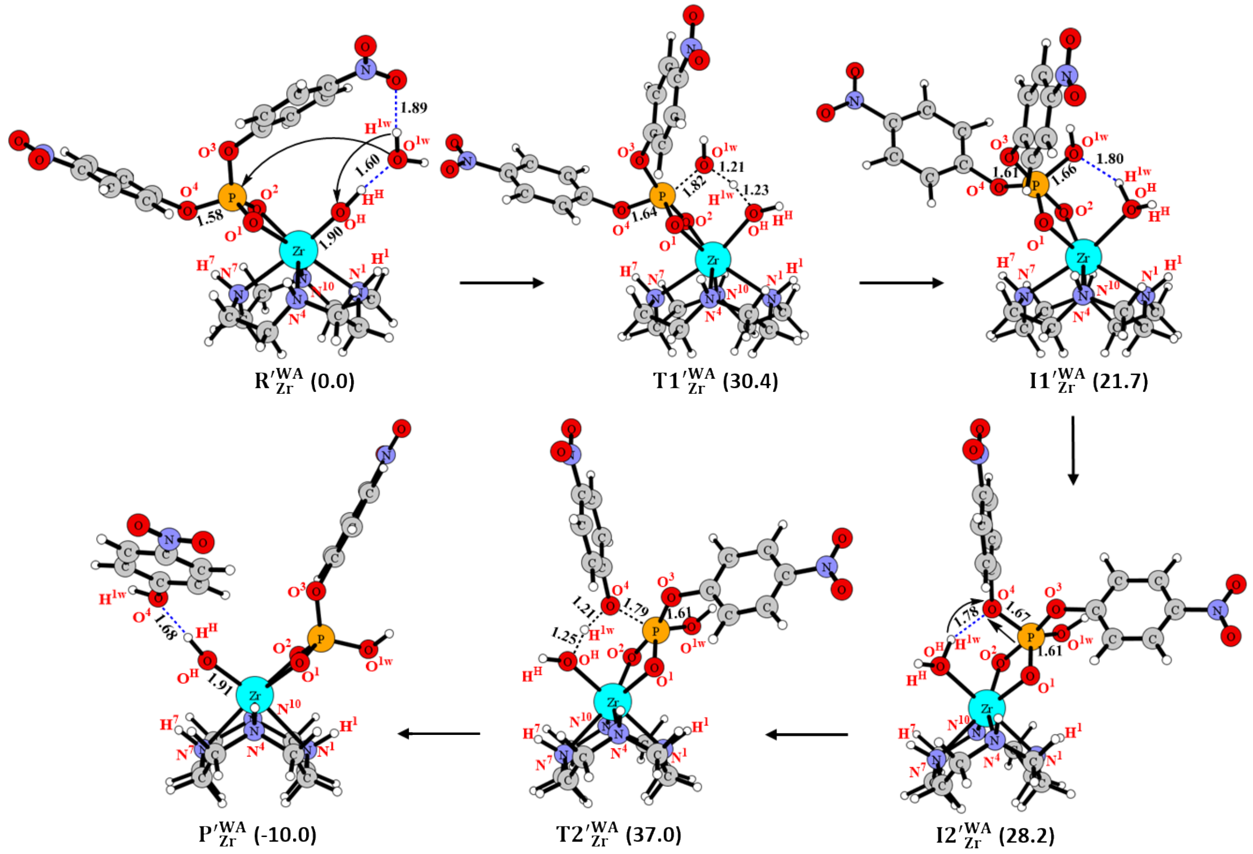

Figure S16. Structures (in Å) and energies (in kcal/mol) in the ***WA*** mechanism for **Zr-C** (coordination number = 7).

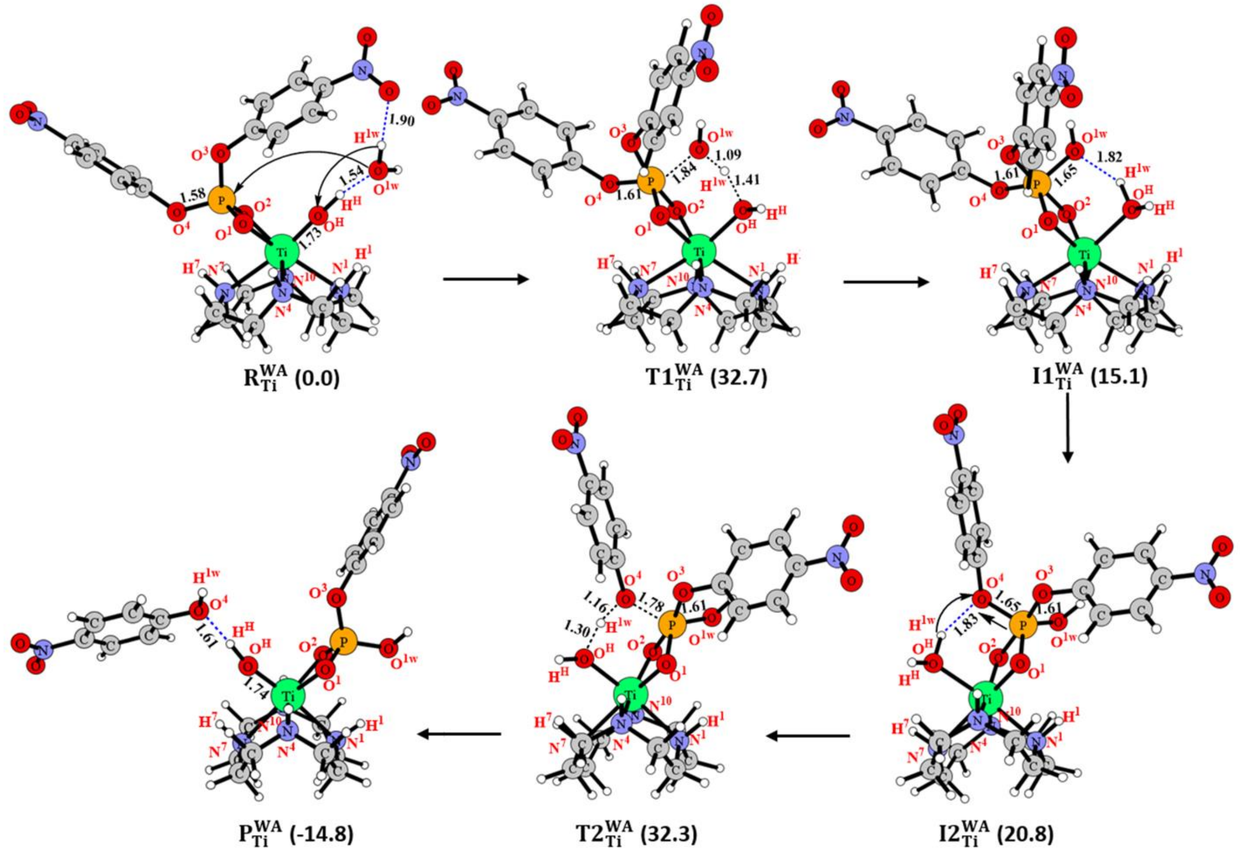

Figure S17. Structures (in Å) and energies (in kcal/mol) in the ***WA*** mechanism for **Ti-C** (coordination number = 7).

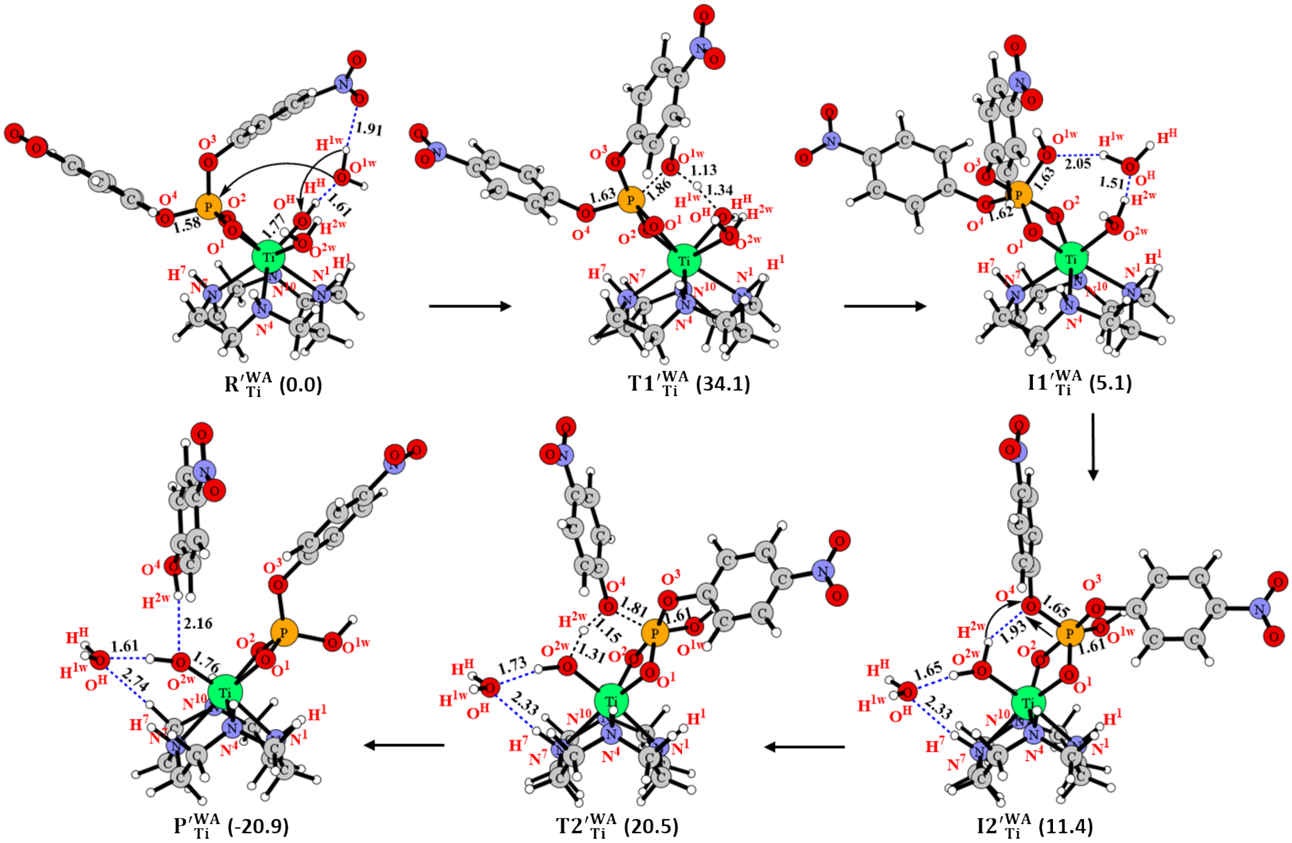

Figure S18. Structures (in Å) and energies (in kcal/mol) in the ***WA*** mechanism for **Ti-C** (coordination number = 8).

Table S1. Key bond distances (in Å) of all optimized structures in the ***DA***, ***CA*** and ***WA*** mechanisms for **Zn-C**, **Cu-C** and **Co-C**.

**DA(CA)**	M-O^1	M-O^H	M-N^1	M-N^4	M-N^7	M-N^{10}	O^H-H^1	N^1-H^1	P-O^H	P-O^4
R_{Zn}	2.15	1.92	2.28	2.28	2.32	2.24	2.02	1.03	3.98	1.64
$T1_{Zn}^{DA}$	1.93	2.47	2.12	2.23	2.22	2.17	1.75	1.05	2.12	1.73
$I1_{Zn}^{CA}$	1.99	3.24	1.97	2.22	2.23	2.23	0.99	1.85	4.57	1.63
$T2_{Zn}^{CA}$	1.93	2.78	2.07	2.18	2.17	2.14	1.52	1.10	2.29	1.72
R_{Cu}	2.16	1.88	2.27	2.30	2.12	2.29	2.02	1.02	3.84	1.64
$T1_{Cu}^{DA}$	2.06	2.54	2.04	2.11	2.19	2.05	1.81	1.04	2.01	1.77
$I1_{Cu}^{CA}$	2.02	3.20	1.94	2.12	2.32	2.12	0.99	1.93	4.7	1.64
$T2_{Cu}^{CA}$	1.97	2.96	2.05	2.04	2.12	2.03	1.46	1.13	2.24	1.73
R_{Co}	2.08	1.90	2.10	2.25	2.03	2.22	1.93	1.03	3.50	1.65
$T1_{Co}^{DA}$	1.94	2.49	1.99	2.04	2.16	2.03	2.67	1.03	2.15	1.70
$I1_{Co}^{CA}$	2.05	3.18	1.88	2.03	2.16	2.03	0.99	1.93	4.64	1.64
$T2_{Co}^{CA}$	1.97	3.12	2.03	1.99	2.05	2.00	1.38	1.17	2.27	1.72
**WA**	M-O^1	M-O^H	M-N^1	M-N^4	M-N^7	M-N^{10}	O^H-H^w	O^w-H^w	P-O^w	P-O^4
R_{Zn}^{WA}	2.15	1.97	2.28	2.23	2.28	2.23	1.60	1.02	4.86	1.66
T_{Zn}^{WA}	2.03	2.08	2.28	2.22	2.27	2.20	1.17	1.28	1.96	1.84
R_{Cu}^{WA}	2.31	1.91	2.33	2.20	2.08	2.20	1.62	1.01	4.63	1.66
T_{Cu}^{WA}	2.00	1.98	2.21	2.38	2.10	2.34	1.28	1.17	2.03	1.85
R_{Co}^{WA}	2.19	1.92	2.15	2.16	2.00	2.16	1.61	1.02	4.43	1.67
T_{Co}^{WA}	1.96	2.01	2.04	2.27	2.04	2.27	1.14	1.31	1.94	1.86

Table S2. Key charges of the optimized structures in the ***DA***, ***CA*** and ***WA*** mechanisms for **Zn-C**, **Cu-C** and **Co-C**.

DA(CA)	P	Metal	O^H	H^1	N^1
R_{Zn}	2.52	1.24	-1.21	0.45	-0.77
$I1_{Zn}^{CA}$	2.53	1.25	-0.99	0.50	-0.97
R_{Cu}	2.52	0.96	-1.07	0.45	-0.72
$I1_{Cu}^{CA}$	2.53	0.88	-0.99	0.50	-0.76
R_{Co}	2.52	0.69	-1.02	0.46	-0.69
$I1_{Co}^{CA}$	2.53	0.65	-0.99	0.50	-0.73
**WA**	**P**	**Metal**	O^H	O^w	**-**
R_{Zn}^{WA}	2.52	1.27	-1.20	-1.02	-
R_{Cu}^{WA}	2.52	0.99	-1.10	-1.02	-
R_{Co}^{WA}	2.52	0.70	-1.04	-1.03	-

Table S3. Key bond distances (in Å) of all optimized structures in the ***DA***, ***CA*** and ***WA*** mechanisms for **Ce-C**.

**DA(CA)**	Ce-O[1]	Ce-O[2]	Ce-O[H]	Ce-N[1]	Ce-N[4]	Ce-N[7]	Ce-N[10]	P-O[H]	P-O[4]	N[1]-H[1]
$\mathrm{R_{Ce}}$	2.33	2.33	2.04	2.64	2.58	2.63	2.58	4.20	1.57	1.02
$\mathrm{T1_{Ce}^{DA}}$	2.25	2.06	2.46	2.63	2.61	2.59	2.60	1.88	1.62	1.02
$\mathrm{I1_{Ce}^{CA}}$	2.38	2.37	2.50	2.22	2.56	2.60	2.55	4.00	1.57	3.61
$\mathrm{T2_{Ce}^{CA}}$	2.24	2.24	3.34	2.34	2.57	2.68	2.56	1.98	1.69	1.34
$\mathrm{R'_{Ce}}$	2.46	2.40	2.04	2.67	2.61	2.68	2.61	3.59	1.57	1.02
$\mathrm{T1'^{DA}_{Ce}}$	2.17	2.17	2.71	2.56	2.62	2.63	2.63	1.83	1.68	1.02
$\mathrm{I1'^{CA}_{Ce}}$	2.49	2.42	2.51	2.23	2.60	2.68	2.56	3.55	1.58	3.03
$\mathrm{T2'^{CA}_{Ce}}$	2.24	2.24	3.37	2.38	2.57	2.68	2.57	1.98	1.70	1.33
$\mathrm{R''_{Ce}}$	2.37	2.56	2.05	2.66	2.63	2.72	2.63	3.49	1.59	1.02
$\mathrm{T1''^{DA}_{Ce}}$	2.25	2.28	2.75	2.61	2.61	2.66	2.66	1.80	1.74	1.02
$\mathrm{I1''^{CA}_{Ce}}$	2.41	2.61	2.54	2.25	2.57	2.68	2.62	3.51	1.59	3.14
$\mathrm{T2''^{CA}_{Ce}}$	2.27	2.28	3.38	2.40	2.57	2.68	2.58	2.01	1.69	1.33
**WA**	Ce-O[1]	Ce-O[2]	Ce-O[H]	Ce-N[1]	Ce-N[4]	Ce-N[7]	Ce-N[10]	P-O[1w]	P-O[4]	O[1w]-H[1w]
$\mathrm{R_{Ce}^{WA}}$	2.39	2.36	1.98	2.65	2.64	2.65	2.63	5.90	1.58	0.98
$\mathrm{T1_{Ce}^{WA}}$	2.21	2.21	2.30	2.66	2.61	2.56	2.60	1.85	1.71	1.28
$\mathrm{T2_{Ce}^{WA}}$	2.25	2.24	2.50	2.67	2.65	2.56	2.61	1.68	2.03	1.76
$\mathrm{R'^{WA}_{Ce}}$	2.45	2.41	1.99	2.65	2.69	2.69	2.63	5.88	1.58	0.98
$\mathrm{T1'^{WA}_{Ce}}$	2.30	2.26	2.32	2.67	2.65	2.59	2.61	1.87	1.73	1.27
$\mathrm{T2'^{WA}_{Ce}}$	2.35	2.31	2.48	2.69	2.66	2.58	2.62	1.70	2.02	1.70
$\mathrm{R''^{WA}_{Ce}}$	2.52	2.53	2.05	2.69	2.66	2.69	2.68	5.74	1.58	0.99
$\mathrm{T1''^{WA}_{Ce}}$	2.29	2.31	2.35	2.69	2.64	2.60	2.62	1.90	1.71	1.27
$\mathrm{T2''^{WA}_{Ce}}$	2.34	2.38	2.61	2.74	2.67	2.62	2.63	1.71	1.99	1.70

Table S4. Key charges of the optimized structures in the ***DA***, ***CA*** and ***WA*** mechanisms for **Ce-C**.

**DA(CA)**	**P**	**Metal**	**O^H**	**H^1**	**N^1**
R_{Ce}	2.59	1.69	-0.92	0.44	-0.71
$I1_{Ce}^{CA}$	2.58	1.70	-0.93	0.55	-0.67
R'_{Ce}	2.60	1.59	-0.91	0.44	-0.69
$I1'^{CA}_{Ce}$	2.59	1.57	-0.91	0.54	-0.63
R''_{Ce}	2.60	1.43	-0.89	0.44	-0.69
$I1''^{CA}_{Ce}$	2.59	1.44	-0.90	0.54	-0.62
**WA**	**P**	**Metal**	**O^H**	**O^{1w}**	**-**
R_{Ce}^{WA}	2.58	1.78	-0.94	-0.98	-
R'^{WA}_{Ce}	2.58	1.60	-0.92	-0.99	-
R''^{WA}_{Ce}	2.58	1.51	-0.92	-1.00	-

Table S5. Key bond distances (Å) of all optimized structures in the ***DA*** and ***WA*** mechanisms for **Zr-C** and **Ti-C**.

**DA**	M-O^1	M-O^2	M-O^H	M-N^1	M-N^4	M-N^7	M-N^{10}	P-O^H	P-O^4	O^4-H^w	O^w-H^w
R_{Zr}	2.31	2.21	1.99	2.44	2.40	2.48	2.40	4.22	1.57	5.74	0.97
$T1_{Zr}^{DA}$	2.05	2.09	2.71	2.35	2.39	2.40	2.43	1.82	1.70	1.77	1.00
$T2_{Zr}^{DA}$	2.14	1.99	3.87	2.39	2.39	2.45	2.38	1.61	1.79	1.21	1.25
R'_{Zr}	2.22	2.22	1.94	2.43	2.37	2.41	2.37	3.68	1.58	2.07	0.97
$T1'^{DA}_{Zr}$	2.20	1.93	2.46	2.37	2.38	2.32	2.38	1.78	1.61	2.85	0.97
$T2'^{DA}_{Zr}$	2.14	1.99	3.87	2.39	2.39	2.45	2.38	1.61	1.79	1.21	1.25
R_{Ti}	2.09	2.08	1.78	2.30	2.23	2.25	2.23	3.36	1.58	2.13	0.97
$T1_{Ti}^{DA}$	2.07	1.78	2.29	2.20	2.23	2.16	2.23	1.84	1.63	2.18	0.97
$T2_{Ti}^{DA}$	2.03	1.82	3.75	2.23	2.26	2.32	2.26	1.61	1.78	1.16	1.30
R'_{Ti}	2.16	2.08	1.84	2.31	2.25	2.34	2.26	3.85	1.57	5.68	0.97
$T1'^{DA}_{Ti}$	1.96	1.89	2.49	2.20	2.24	2.28	2.28	1.85	1.65	2.38	0.98
$T2'^{DA}_{Ti}$	2.03	1.82	3.75	2.23	2.26	2.32	2.26	1.61	1.78	1.16	1.30
**WA**	M-O^1	M-O^2	M-O^H	M-N^1	M-N^4	M-N^7	M-N^{10}	P-O^{1w}	P-O^4	O^{1w}-H^{1w}	O^H-H^{1w}
R_{Zr}^{WA}	2.24	2.29	1.92	2.44	2.52	2.54	2.41	5.70	1.58	0.98	3.31
$T1_{Zr}^{WA}$	2.15	2.08	2.17	2.45	2.46	2.44	2.40	1.83	1.63	1.19	1.26
$T2_{Zr}^{WA}$	2.16	2.05	2.43	2.45	2.41	2.45	2.40	1.62	1.84	6.13	0.97
R'^{WA}_{Zr}	2.22	2.22	1.90	2.44	2.41	2.51	2.41	5.67	1.58	0.98	3.31
$T1'^{WA}_{Zr}$	2.14	2.00	2.15	2.43	2.41	2.40	2.39	1.82	1.64	1.21	1.23
$T2'^{WA}_{Zr}$	2.14	1.99	2.14	2.39	2.39	2.45	2.38	1.61	1.79	3.88	1.25
R_{Ti}^{WA}	2.07	2.09	1.73	2.33	2.27	2.38	2.26	5.39	1.58	0.98	3.26
$T1_{Ti}^{WA}$	2.09	1.80	1.98	2.31	2.27	2.27	2.27	1.84	1.61	1.09	1.41
$T2_{Ti}^{WA}$	2.03	1.82	1.97	2.23	2.26	2.32	2.26	1.61	1.78	3.76	1.30
R'^{WA}_{Ti}	2.05	2.22	1.77	2.29	2.42	2.41	2.28	5.30	1.58	0.98	3.27
$T1'^{WA}_{Ti}$	1.97	1.93	2.11	2.31	2.34	2.32	2.30	1.86	1.63	1.13	1.34
$T2'^{WA}_{Ti}$	2.03	1.85	4.12	2.24	2.26	2.33	2.26	1.61	1.81	7.90	0.97

Table S6. Key charges of the optimized structures in the ***DA*** and ***WA*** mechanisms for **Zr-C** and **Ti-C**.

**DA**	**P**	**Metal**	**O^H**	**O^w**
R_{Zr}	2.59	1.74	-1.01	-0.91
R'_{Zr}	2.60	1.83	-0.97	-0.99
R_{Ti}	2.60	1.27	-0.83	-0.99
R'_{Ti}	2.59	1.27	-0.87	-0.91
**WA**	**P**	**Metal**	**O^H**	**O^{1w}**
R_{Zr}^{WA}	2.59	1.74	-1.01	-1.00
R'^{WA}_{Zr}	2.59	1.86	-0.97	-0.99
R_{Ti}^{WA}	2.58	1.29	-0.79	-0.98
R'^{WA}_{Ti}	2.58	1.28	-0.87	-0.99

Cartesian coordinates of the optimized structures reported in this study:

R_{Zn}
Charge = 0, Multiplicity = 1
Energy = -2353.884166 hartree

O	-2.95561200	1.04678800	0.34328200
C	-1.16887100	-2.99250800	1.65921200
H	-1.21730900	-3.59637900	2.57860600
H	-0.10981300	-2.85932600	1.41735800
C	-4.20982000	-1.85945600	2.19332000
H	-4.27020700	-2.93496700	1.98954000
H	-4.99534000	-1.63676200	2.93441500
C	-2.84090500	-1.50468100	2.81127100
H	-2.85929100	-0.45361300	3.11597700
H	-2.68697800	-2.11454500	3.71524200
C	-5.13707600	-0.96184200	-1.39525100
H	-5.83957500	-1.39267200	-2.12564500
H	-5.36112900	0.10500000	-1.30314200
C	-1.85482900	-3.77239100	0.52137600
H	-1.29247600	-4.70245700	0.34078600
H	-2.86518600	-4.06994100	0.82056600
C	-3.33810300	-2.24808300	-2.62064700
H	-2.47706300	-1.96212500	-3.23469000
H	-4.12933000	-2.59853100	-3.30241700
C	-5.37512800	-1.61884500	-0.02006200
H	-6.41501300	-1.40319400	0.28000300
H	-5.29362500	-2.71013400	-0.08912600
C	-2.92464200	-3.40721500	-1.69579000
H	-3.80257900	-3.80768200	-1.17870700
H	-2.52302100	-4.22677600	-2.31234300
N	-3.74257200	-1.05001400	-1.87316700
H	-3.53103000	-0.21881800	-2.41669700
H	-2.34610900	1.76672200	0.14081300
N	-1.98142200	-2.94260000	-0.67884900
H	-1.06816300	-2.74802800	-1.08980100
N	-1.72468200	-1.64751000	1.86373100
H	-0.95583500	-1.01371400	2.09393500
N	-4.38448200	-1.14817300	0.93461900
H	-4.42892800	-0.13265600	1.06750100
C	-0.07068900	5.09476900	-0.01297500
C	0.62636800	4.81263700	-1.18980500
C	1.08845800	3.52145800	-1.40426400
C	0.85518000	2.52590100	-0.44495100
C	0.16489400	2.82096600	0.74052700
C	-0.30373000	4.11455300	0.95122200
H	0.79117000	5.59924600	-1.91582500
H	1.63097100	3.26071000	-2.30647100
H	0.01965400	2.04984400	1.48739800
H	-0.84127200	4.37224100	1.85559000
N	-0.56756500	6.45742000	0.21545800
O	-1.16937100	6.67899100	1.26011000
O	-0.35252600	7.29630900	-0.65266900
O	1.36869000	1.28897600	-0.72832500
P	0.80012500	-0.16528700	-0.18714300
O	-0.43873600	-0.54168800	-0.96870400
O	0.78379100	-0.23099100	1.30649100
O	1.98176700	-1.09398300	-0.84816400
C	3.30689400	-1.15535300	-0.49447600
C	3.78186800	-0.85049700	0.78890100
C	4.18122000	-1.59498100	-1.49775600
C	5.14081600	-0.98111900	1.05759000
H	3.08994700	-0.52710500	1.55755400
C	5.53589300	-1.73025300	-1.22698400
H	3.77884300	-1.82123700	-2.47925900
C	6.00192100	-1.41976700	0.05197800
H	5.53979600	-0.75313100	2.03852600
H	6.23317200	-2.06566200	-1.98478900
N	7.43370800	-1.56347300	0.34535900
O	8.16459500	-1.95633200	-0.55743700
O	7.81653100	-1.28444800	1.47584300
Zn	-2.37861300	-0.73511000	-0.07386100

$T1^{DA}_{Zn}$
Charge = 0, Multiplicity = 1
Energy = -2353.8361992 hartree

O	-1.02406500	1.16491000	0.09819200
C	-3.06328300	-2.25078900	2.30241900
H	-3.29176900	-2.32871200	3.37615600
H	-2.33717000	-3.03746200	2.06742600
C	-4.00518400	1.01652700	1.86248000
H	-4.96947600	0.49725200	1.94505500
H	-4.13874000	2.00339800	2.33165300
C	-2.90546800	0.25108400	2.63732600
H	-2.03043900	0.89764300	2.73512400
H	-3.27237300	0.03004600	3.64978800
C	-4.32544300	1.11485100	-1.92569700
H	-5.17282600	1.31816300	-2.59674800
H	-3.47620100	1.71536800	-2.26575400
C	-4.35988900	-2.49436400	1.50568600
H	-4.75539600	-3.49416800	1.73079200
H	-5.12168900	-1.77174700	1.81427100
C	-4.92121600	-1.34564600	-2.10472700
H	-4.50866300	-2.15187300	-2.72170100
H	-5.82688000	-0.98213200	-2.61216600
C	-4.69222600	1.54949700	-0.48945800
H	-4.84223200	2.63948400	-0.48434700
H	-5.64741600	1.10077300	-0.18639200
C	-5.32227800	-1.91898000	-0.73192500
H	-5.85740800	-1.15883800	-0.15478500
H	-6.01646900	-2.75874500	-0.88052100
N	-3.89362300	-0.29644400	-2.00631400
H	-3.20650100	-0.40504100	-2.74681100
H	-0.61874000	1.66187600	0.82206300
N	-4.13556000	-2.30411100	0.05479900
H	-3.70943100	-3.14190600	-0.34228000
N	-2.43915600	-0.97209200	1.95737200
H	-1.40528100	-1.01439500	1.98493700
N	-3.66036200	1.11081600	0.44307200
H	-2.71693400	1.54431400	0.29590800
C	2.34008600	4.40315000	-0.03596600
C	2.06143700	4.05563300	-1.36035100

C	1.66815800	2.75629300	-1.64824400
C	1.55414900	1.81020200	-0.61864200
C	1.86085500	2.16658900	0.70641600
C	2.24775400	3.46863400	0.99793500
H	2.15086700	4.80430500	-2.13802900
H	1.44018100	2.44899900	-2.66303200
H	1.79195300	1.41712000	1.48729700
H	2.48771200	3.77040700	2.01035700
N	2.73472900	5.77841400	0.27527700
O	2.95551200	6.06124000	1.44911800
O	2.81800400	6.57640200	-0.65375500
O	1.23738700	0.54171200	-0.97546100
P	0.20475000	-0.53487800	-0.21762300
O	-1.00250400	-1.01305800	-1.07279500
O	0.30008300	-0.70169600	1.28453700
O	1.19421500	-1.81913300	-0.80778700
C	2.47637100	-2.08492700	-0.50316800
C	3.09364500	-1.75265900	0.72079100
C	3.21462400	-2.78731000	-1.47882300
C	4.41757000	-2.10549300	0.94828900
H	2.51367300	-1.24820400	1.48311500
C	4.53330800	-3.14391300	-1.25041000
H	2.72144800	-3.03503500	-2.41299700
C	5.13072100	-2.79632200	-0.03416700
H	4.90707500	-1.86058300	1.88339900
H	5.11120400	-3.67946000	-1.99387800
N	6.51946400	-3.16906800	0.21411600
O	7.11859500	-3.78380700	-0.66609100
O	7.02055700	-2.85168200	1.29061400
Zn	-2.67054000	-0.65539200	-0.17643200

$T1_{Zn}^{CA}$
Charge = 0, Multiplicity = 1
Energy = -2353.8613274 hartree

O	-2.70939600	1.22864000	-0.13370200
C	-1.59883900	-2.88073000	2.03969100
H	-1.77386200	-3.26850200	3.05520300
H	-0.53935700	-3.03895200	1.80993600
C	-4.30879900	-0.94389900	2.09215200
H	-4.68708000	-1.98362600	2.13158500
H	-5.01459300	-0.35458300	2.70753500
C	-2.92868300	-0.87095900	2.78604300
H	-2.67751100	0.18152900	2.95720900
H	-2.98003700	-1.36050400	3.77045400
C	-4.88947800	-0.60710200	-1.62467700
H	-5.65683200	-1.01839700	-2.29747800
H	-4.83674400	0.47250600	-1.78927200
C	-2.45730700	-3.69225000	1.05207400
H	-2.16731500	-4.75271100	1.11478600
H	-3.51204300	-3.63023200	1.33551300
C	-3.45356100	-2.53299300	-2.42831200
H	-2.53700900	-2.61564800	-3.02339100
H	-4.29745000	-2.80599600	-3.08075800
C	-5.29192000	-0.85228300	-0.15332800
H	-6.22097100	-0.27674700	0.02112000
H	-5.57544600	-1.91310900	-0.01072800
C	-3.38500600	-3.53638200	-1.26336200
H	-4.33602200	-3.54461700	-0.72314600
H	-3.23917700	-4.54801300	-1.67239800
N	-3.54589300	-1.13690400	-1.97155500
H	-3.11985100	-0.52723700	-2.66322300
H	-2.22219300	1.90714400	0.35203000
N	-2.33792600	-3.15393000	-0.30913600
H	-1.41095600	-3.33599800	-0.69304400
N	-1.83486000	-1.43109500	1.96720200
H	-0.94999700	-0.94787800	2.13752100
N	-4.20802500	-0.48750500	0.72717900
H	-3.58049900	0.70661200	0.43958400
C	-0.03442200	4.84491600	-0.06565400
C	0.32379200	4.47097000	-1.36194400
C	0.75141600	3.17018200	-1.59292800
C	0.82001500	2.25721200	-0.53216000
C	0.47079700	2.64509100	0.77117500
C	0.03742100	3.94786100	1.00047600
H	0.25821300	5.19468900	-2.16502600
H	1.03506500	2.83967000	-2.58606600
H	0.55735400	1.93176100	1.58199700
H	-0.23328300	4.28074100	1.99534800
N	-0.49488100	6.21728300	0.18322700
O	-0.82196300	6.51065300	1.32777000
O	-0.52706400	6.98960000	-0.76780500
O	1.29333300	1.00888600	-0.83888600
P	0.82333000	-0.42925100	-0.17486500
O	-0.40050900	-0.93703300	-0.90840800
O	0.81314700	-0.36743800	1.31962500
O	2.04151100	-1.33976200	-0.77744700
C	3.37681800	-1.27631000	-0.45920600
C	3.85294100	-0.86106600	0.79177100
C	4.25969700	-1.70405500	-1.45925200
C	5.22366800	-0.86587000	1.03094800
H	3.15360300	-0.55047700	1.55900600
C	5.62673600	-1.71407000	-1.21769800
H	3.85476500	-2.02007100	-2.41448500
C	6.09422500	-1.29174500	0.02817800
H	5.62484400	-0.54997600	1.98625300
H	6.33200800	-2.03697500	-1.97352300
N	7.54001200	-1.29988000	0.28939700
O	8.27961100	-1.68591000	-0.60886300
O	7.92379400	-0.92155900	1.39012300
Zn	-2.31677800	-0.90263800	-0.13556400

$I1_{Zn}^{CA}$
Charge = 0, Multiplicity = 1
Energy = -2353.8653026 hartree

O	-3.20205300	1.78836400	-0.63757600
C	-1.49919100	-3.12032700	2.14654000
H	-1.73180400	-3.46029900	3.16785100
H	-0.44059300	-3.34261100	1.96591100
C	-4.10132100	-1.02665400	1.96631200
H	-4.54380900	-2.04441600	2.03429400
H	-4.80245100	-0.37418700	2.51929400
C	-2.76273400	-1.00810800	2.73655300

H	-2.45232500	0.03341200	2.87317600
H	-2.89645500	-1.44965600	3.73577700
C	-4.57533000	-0.87056500	-1.76794600
H	-5.34944100	-1.30114000	-2.42010800
H	-4.46944300	0.19065400	-2.00546700
C	-2.36094900	-3.93089700	1.15946400
H	-2.13049300	-5.00100600	1.27080600
H	-3.41973800	-3.79971400	1.39945600
C	-3.20599200	-2.90868800	-2.41508400
H	-2.27910300	-3.06894600	-2.97838700
H	-4.04332000	-3.18890500	-3.07292600
C	-4.99757500	-0.99644400	-0.28735800
H	-5.89390400	-0.36191000	-0.16004000
H	-5.34847200	-2.03231300	-0.08538300
C	-3.21655400	-3.83987200	-1.18931600
H	-4.17707500	-3.75864600	-0.67341400
H	-3.11596300	-4.88200700	-1.52695000
N	-3.25110000	-1.48575100	-2.04731700
H	-2.76531700	-0.93472300	-2.74848700
H	-2.51498500	2.30431600	-0.19529400
N	-2.16724900	-3.46064300	-0.22545000
H	-1.25156100	-3.74955400	-0.57021900
N	-1.66415900	-1.67003100	2.00211000
H	-0.77215700	-1.19729300	2.16361300
N	-3.91034100	-0.62059400	0.59295600
H	-3.49214900	1.07988900	-0.00506300
C	-0.41042700	4.92143300	-0.00043300
C	0.17179700	4.62096300	-1.23295800
C	0.72828300	3.36319300	-1.42539000
C	0.70110500	2.42175400	-0.38877800
C	0.12396200	2.73481200	0.85135400
C	-0.43947100	3.99421100	1.04127500
H	0.17576000	5.36633200	-2.01867600
H	1.18448200	3.08871300	-2.37007500
H	0.14331400	2.00579700	1.65323300
H	-0.88786100	4.27032100	1.98805400
N	-1.00210700	6.25112600	0.20836600
O	-1.51843800	6.47994300	1.29579900
O	-0.94259800	7.05204400	-0.71657700
O	1.30699100	1.21863600	-0.64708100
P	0.84807900	-0.25202200	-0.06448000
O	-0.39078300	-0.69904300	-0.82052900
O	0.84800700	-0.28975200	1.42883000
O	2.05192700	-1.12594000	-0.73754400
C	3.39590900	-1.06699500	-0.44783300
C	3.89695200	-0.71796800	0.81314800
C	4.25705800	-1.42823700	-1.49137200
C	5.27326700	-0.72132800	1.01910000
H	3.21414100	-0.45697600	1.61324500
C	5.62977400	-1.43698900	-1.28306900
H	3.83213100	-1.69335600	-2.45336500
C	6.12279600	-1.08054500	-0.02669700
H	5.69458000	-0.45498000	1.98079000
H	6.31972700	-1.70865200	-2.07243200
N	7.57520700	-1.08763700	0.19925300
O	8.29568800	-1.41375200	-0.73718500
O	7.98151100	-0.76848900	1.31030600
Zn	-2.17996000	-1.23785600	-0.12413400

$T2_{Zn}^{CA}$
Charge = 0, Multiplicity = 1
Energy = -2353.8317626 hartree

O	-1.00441800	1.49350600	0.07108000
C	-3.21317700	-2.06088300	2.36479400
H	-3.41509700	-2.06449300	3.44625300
H	-2.56960000	-2.92254800	2.15156100
C	-3.89199600	1.23895600	1.78222600
H	-4.89652700	0.80732400	1.90973600
H	-3.93440300	2.25691200	2.20089000
C	-2.83663500	0.43416700	2.57227500
H	-1.90927600	1.00988600	2.58655300
H	-3.17336100	0.28931200	3.60822500
C	-4.19798000	1.18595900	-1.99310300
H	-5.00397100	1.44112800	-2.69603600
H	-3.27914100	1.67250600	-2.33339300
C	-4.54741200	-2.22144500	1.60939300
H	-5.02778300	-3.16896700	1.88764800
H	-5.23130000	-1.41567100	1.89163000
C	-5.04743500	-1.21146200	-2.05576000
H	-4.72389900	-2.08270000	-2.63682900
H	-5.91350400	-0.77871300	-2.57682300
C	-4.53333200	1.71541800	-0.58456600
H	-4.58140900	2.81424000	-0.63144400
H	-5.53169800	1.37270900	-0.27387700
C	-5.49201300	-1.68010900	-0.65588200
H	-5.95619000	-0.85012800	-0.11551700
H	-6.25132000	-2.46808400	-0.75964300
N	-3.91435400	-0.27056200	-2.00224900
H	-3.25177600	-0.47469000	-2.74543500
H	-0.56001100	2.07467600	0.70200600
N	-4.33302500	-2.12356100	0.14561600
H	-3.98621600	-3.01547500	-0.20927900
N	-2.48849300	-0.85887800	1.93821300
H	-1.46350800	-0.97323800	1.98435000
N	-3.54105000	1.22947500	0.36425800
H	-2.51146300	1.59774000	0.21583000
C	2.71952400	4.29813100	-0.04043200
C	2.47106800	3.95487000	-1.37158000
C	1.94456700	2.70308800	-1.65807800
C	1.66906700	1.80215900	-0.61985900
C	1.94113600	2.15060600	0.71405000
C	2.46174100	3.40585700	1.00269600
H	2.68785500	4.66928300	-2.15627300
H	1.73595500	2.40032600	-2.67826500
H	1.73519500	1.43646800	1.50319900
H	2.67849800	3.70273900	2.02176600
N	3.26002400	5.62371900	0.26823800
O	3.45224300	5.90536500	1.44740500
O	3.48790300	6.38342300	-0.66842100
O	1.23458700	0.56775100	-0.98352100
P	0.16933300	-0.44614200	-0.21804300
O	-1.08408800	-0.80752300	-1.04626000

O	0.24531900	-0.56410800	1.28222300
O	1.05019100	-1.80250600	-0.79477000
C	2.31196500	-2.16573800	-0.49476500
C	3.00360200	-1.77528500	0.66925900
C	2.93955100	-3.03032300	-1.41480500
C	4.29504900	-2.23536900	0.89438200
H	2.50872500	-1.14351600	1.39559100
C	4.22511600	-3.49345500	-1.18835600
H	2.38929400	-3.31794600	-2.30458500
C	4.89856600	-3.08925000	-0.03107000
H	4.84142000	-1.94822600	1.78486800
H	4.71930800	-4.15557600	-1.88890000
N	6.25286700	-3.57620600	0.21632700
O	6.75302900	-4.33441900	-0.61159200
O	6.82242100	-3.20547500	1.23986100
Zn	-2.78999700	-0.62948400	-0.16729800

$I_{Zn}^{DA}/I2_{Zn}^{CA}$
Charge = 0, Multiplicity = 1
Energy = -2353.8340468 hartree

O	-0.59029000	1.61309800	0.12139100
C	-3.30628300	-1.86496400	2.33403400
H	-3.50138600	-1.87877100	3.41703000
H	-2.71406100	-2.75713600	2.10042600
C	-3.70520600	1.53224600	1.83174700
H	-4.74410200	1.19258600	1.93627100
H	-3.65423500	2.53593900	2.27920400
C	-2.74743800	0.59329400	2.59731800
H	-1.76511000	1.06615200	2.66460300
H	-3.12334900	0.44950700	3.62009100
C	-4.18955200	1.56098700	-1.93670700
H	-5.02344100	1.90112900	-2.56834700
H	-3.26471800	1.97455000	-2.34893200
C	-4.65881900	-1.93488500	1.59899000
H	-5.18387400	-2.86109600	1.86789300
H	-5.29284400	-1.10222600	1.91814500
C	-5.23310000	-0.76423200	-2.00937600
H	-5.00589300	-1.63280500	-2.63745300
H	-6.09656700	-0.25578400	-2.46321500
C	-4.38107100	2.10293400	-0.50625700
H	-4.35298700	3.20160300	-0.54043300
H	-5.37351300	1.82567600	-0.12886500
C	-5.63698700	-1.25006700	-0.60382700
H	-6.00654500	-0.40661700	-0.01365700
H	-6.46303400	-1.96970900	-0.69329300
N	-4.03947500	0.09119100	-1.98688600
H	-3.40166500	-0.15426200	-2.73838300
H	-0.08454700	2.03199900	0.83151500
N	-4.48311600	-1.81606800	0.12873300
H	-4.24430400	-2.72903200	-0.25797200
N	-2.52201400	-0.70119100	1.92639200
H	-1.49632200	-0.87096600	1.90123900
N	-3.37634900	1.53795600	0.39946100
H	-2.41228800	1.90677600	0.24485300
C	3.46736500	4.08463700	-0.02509800
C	3.15554400	3.78882000	-1.35509800
C	2.44693600	2.63076200	-1.64149700
C	2.05353800	1.76903500	-0.60495200
C	2.39585900	2.06841900	0.72697700
C	3.09797500	3.23179700	1.01709600
H	3.46501000	4.46992900	-2.13847300
H	2.18369300	2.36950700	-2.66069500
H	2.11927600	1.37601900	1.51563400
H	3.37290100	3.48421200	2.03414200
N	4.19467300	5.31744100	0.28285400
O	4.43875400	5.56172000	1.46089900
O	4.51602400	6.04388600	-0.65288400
O	1.41761100	0.62574100	-0.94801300
P	0.07265200	-0.06845900	-0.19421400
O	-1.17559100	-0.31204200	-1.12224300
O	0.14475000	-0.43873400	1.28459000
O	0.74176500	-1.63331700	-0.85128700
C	1.87546100	-2.25078400	-0.54738400
C	2.65109200	-2.01521400	0.61486400
C	2.31568500	-3.24624200	-1.45694000
C	3.81046100	-2.74059000	0.84431300
H	2.30376400	-1.29095600	1.33945500
C	3.46970500	-3.97186900	-1.22649400
H	1.71787600	-3.41652800	-2.34687100
C	4.22037200	-3.71527600	-0.07161100
H	4.40671000	-2.57126400	1.73338300
H	3.80898500	-4.72997600	-1.92234500
N	5.43041700	-4.47677300	0.18112100
O	5.75636000	-5.33957100	-0.63569400
O	6.07111300	-4.23168500	1.20394400
Zn	-2.82884400	-0.41883600	-0.19732300

$T2_{Zn}^{DA}/T3_{Zn}^{CA}$
Charge = 0, Multiplicity = 1
Energy = -2353.8305195 hartree

O	0.16562800	-2.31471000	0.19052800
C	3.24878000	2.25049600	1.23188700
H	3.38052000	2.80523000	2.17189800
H	2.70853600	2.90528500	0.53912800
C	3.48806600	-0.97591400	2.50904000
H	4.54063200	-0.66857300	2.47103500
H	3.37289500	-1.61531700	3.39521900
C	2.56463300	0.25103500	2.65174800
H	1.55280700	-0.09157800	2.87986400
H	2.92083400	0.87371800	3.48358900
C	4.15424700	-2.86302600	-0.72164800
H	5.00290400	-3.48155100	-1.04806900
H	3.23637300	-3.41674400	-0.94059700
C	4.63722400	1.91654400	0.65411600
H	5.19729800	2.83916900	0.45524300
H	5.21232700	1.34178200	1.38742900
C	5.30698500	-0.89832700	-1.86535900
H	5.14266100	-0.45290600	-2.85291200
H	6.15862200	-1.58680200	-1.96070300
C	4.24588400	-2.63673800	0.79937800
H	4.19118500	-3.60926200	1.30771300
H	5.21868700	-2.20124400	1.05552300

C	5.67885500	0.21539300	-0.86527700
H	5.99903400	-0.22948100	0.08204200
H	6.53060100	0.78811800	-1.25734800
N	4.07235300	-1.60213800	-1.48599400
H	3.46799700	-1.73399000	-2.29186500
H	0.19581700	-2.88434200	-0.59290700
N	4.51683100	1.07759900	-0.56521000
H	4.30946700	1.67282000	-1.36816400
N	2.43808800	1.04190400	1.40667300
H	1.42956600	1.19497200	1.26341900
N	3.20382300	-1.70388000	1.25746000
H	2.27933000	-2.15538700	1.28679800
C	-4.82263500	-3.00103900	0.01169300
C	-4.58100200	-2.71882600	-1.33525600
C	-3.42658200	-2.03250800	-1.68177800
C	-2.51662100	-1.63354700	-0.68882500
C	-2.77646000	-1.91018100	0.66446600
C	-3.93316500	-2.59779400	1.00981600
H	-5.29387900	-3.03924900	-2.08506700
H	-3.20591500	-1.78956400	-2.71572800
H	-2.07177000	-1.59611000	1.42437100
H	-4.15670800	-2.82753500	2.04453300
N	-6.02966400	-3.74046100	0.38239300
O	-6.21601100	-3.97898700	1.57221000
O	-6.79013800	-4.08748600	-0.51703800
O	-1.42805800	-0.94787000	-1.13011700
P	-0.01604400	-0.64227300	-0.28600900
O	1.22928600	-0.68312200	-1.25822500
O	-0.00663800	-0.11781900	1.13497100
O	-0.22177100	1.18248300	-1.00891000
C	-1.08621800	2.11388400	-0.68538400
C	-2.10313900	1.98455400	0.30201000
C	-0.99521300	3.35986000	-1.37231500
C	-2.95763100	3.03745400	0.58319900
H	-2.18762100	1.06196000	0.86059100
C	-1.84601300	4.41022400	-1.08979300
H	-0.23204500	3.45418500	-2.13979400
C	-2.83188600	4.25117000	-0.10487100
H	-3.72856100	2.94086100	1.33903400
H	-1.77169000	5.35526800	-1.61515900
N	-3.71627800	5.35282300	0.20829400
O	-3.56948500	6.41252900	-0.40662400
O	-4.57380500	5.18689400	1.07821300
Zn	2.80146500	-0.25173800	-0.26651400

P_{Zn}
Charge = 0, Multiplicity = 1
Energy = -2353.9050554 hartree

O	-0.63322700	-3.67463000	0.35736600
C	2.78153000	0.95897700	2.39873700
H	2.97576100	0.96230300	3.48178500
H	2.05267600	1.75051500	2.19209300
C	3.86314500	-2.17878400	1.73814300
H	4.77420000	-1.57687500	1.82583600
H	4.09562700	-3.16489900	2.17105900
C	2.71214800	-1.55065400	2.54844100
H	1.87830300	-2.25745600	2.59207200
H	3.05309500	-1.38513300	3.58116500
C	4.15052600	-2.00418500	-2.03953400
H	5.00266100	-2.08983300	-2.73092600
H	3.35520100	-2.66014400	-2.40830300
C	4.09395700	1.27153100	1.65154300
H	4.44749200	2.26876100	1.95541500
H	4.87498400	0.56027900	1.94037400
C	4.55835800	0.48551500	-2.05509400
H	4.03776700	1.32419000	-2.52663800
H	5.43439500	0.24335900	-2.67524600
C	4.58199100	-2.48432400	-0.64137200
H	4.87476800	-3.54371200	-0.70631600
H	5.46831000	-1.93605700	-0.30590200
C	5.03638000	0.93628100	-0.66024300
H	5.67260200	0.17418700	-0.19745700
H	5.66215800	1.83475200	-0.78524800
N	3.59873400	-0.63785200	-2.02038800
H	2.93766000	-0.52699800	-2.78474900
H	-1.16544500	-3.92018800	1.12770600
N	3.88503600	1.15247000	0.21009100
H	3.30195600	1.90858000	-0.14870300
N	2.17004000	-0.30851300	1.96155300
H	1.15880700	-0.31217000	2.12363600
N	3.50917400	-2.24438800	0.32153200
H	2.72888100	-2.88277100	0.16365000
C	-5.69234700	-1.48181000	-0.21738100
C	-5.15935200	-1.73475800	-1.48319900
C	-3.78037000	-1.77077800	-1.63595000
C	-2.94735000	-1.55231100	-0.52967700
C	-3.49137800	-1.28175800	0.73460100
C	-4.87431500	-1.25204500	0.88814500
H	-5.82521200	-1.90359000	-2.32049500
H	-3.32539500	-1.96721100	-2.60058800
H	-2.83264400	-1.08365700	1.57207400
H	-5.32540400	-1.04517600	1.85102000
N	-7.15099900	-1.45984700	-0.04684100
O	-7.59196500	-1.25809800	1.07891900
O	-7.84443800	-1.64949700	-1.04001000
O	-1.60004000	-1.58371400	-0.77825200
P	-0.40342000	-2.06530500	0.24016500
O	0.86115600	-1.88930600	-0.57852600
O	-0.50871700	-1.42245900	1.59169300
O	1.43639400	1.22374100	-1.12360600
C	0.54764100	2.12128000	-0.77710600
C	-0.38970300	1.92773400	0.27746500
C	0.51243600	3.37143400	-1.46054100
C	-1.27587900	2.92889400	0.64224800
H	-0.42798700	0.96716000	0.78303200
C	-0.37205400	4.37005900	-1.09765400
H	1.20741000	3.52170800	-2.28234100
C	-1.26272800	4.15249900	-0.03723800
H	-1.98785000	2.78019800	1.44587600
H	-0.39070700	5.32246100	-1.61457900
N	-2.17752100	5.20609200	0.36021100
O	-2.14671900	6.26801400	-0.26314300

O	-2.93749300	4.99093700	1.30496000
Zn	2.25535200	-0.39251400	-0.25620300

$\mathbf{R_{Zn}^{WA}}$

Charge = 0, Multiplicity = 1

Energy = -2430.3501042 hartree

O	-2.73020500	-0.50346300	-1.98773400
C	-2.05770600	3.31200700	0.55900600
H	-2.29800900	4.38211500	0.46609100
H	-0.97778900	3.23830200	0.72276700
C	-4.83733300	2.51528100	-1.01370100
H	-5.10269300	2.97434600	-0.05484500
H	-5.61526500	2.82002200	-1.73219600
C	-3.47558900	3.04897400	-1.50410700
H	-3.29971100	2.68218800	-2.52074100
H	-3.51866600	4.14823000	-1.55188200
C	-5.28956800	-0.98785500	0.42898000
H	-5.97849200	-1.39808000	1.18348400
H	-5.36140000	-1.62525900	-0.45830000
C	-2.79082400	2.74354600	1.79042700
H	-2.41891600	3.25773100	2.69079900
H	-3.86374100	2.95557200	1.72764500
C	-3.62109000	-0.92204900	2.31206300
H	-2.65721700	-1.40395000	2.50915500
H	-4.37790400	-1.43189700	2.92882300
C	-5.75197400	0.43533000	0.05477600
H	-6.75660700	0.36674000	-0.39484000
H	-5.85359600	1.05775800	0.95152000
C	-3.53954600	0.55492800	2.74248300
H	-4.52732000	1.02323900	2.67292500
H	-3.23922100	0.60709100	3.80023100
N	-3.88819900	-1.07466800	0.87710000
H	-3.50090900	-1.95382100	0.51184500
H	-1.95735300	-0.34472800	-2.54554100
N	-2.63290500	1.29012100	1.85813800
H	-1.66098500	1.03178900	2.03221300
N	-2.34645500	2.58311800	-0.68619100
H	-1.48485600	2.52428500	-1.23878300
N	-4.77023500	1.07067900	-0.81624800
H	-4.65942800	0.57139200	-1.70163300
C	2.14446100	-4.48210800	-0.08441700
C	0.80108300	-4.13415900	-0.23084300
C	0.47002500	-2.84864800	-0.64713900
C	1.49632100	-1.93260500	-0.90222200
C	2.84181000	-2.29071900	-0.76155900
C	3.17287600	-3.57631300	-0.34802800
H	0.02956900	-4.86588900	-0.02557200
H	-0.57130300	-2.57335400	-0.77361300
H	3.61096900	-1.55840500	-0.98264900
H	4.20418000	-3.88440900	-0.22824000
N	2.48790100	-5.84235700	0.35631100
O	3.67545100	-6.12142500	0.47648100
O	1.56728200	-6.62032700	0.57984400
O	1.21397200	-0.66589200	-1.37441200
P	0.51663400	0.53976400	-0.50882100
O	-0.62682800	0.01201200	0.33007700
O	0.30604200	1.68569200	-1.43858900
O	1.68309200	0.86156700	0.62470400
C	2.92243900	1.40620700	0.42152900
C	3.33333700	1.99238100	-0.78552600
C	3.78894000	1.36013300	1.52454700
C	4.61406700	2.52829000	-0.87923100
H	2.65016600	2.04441500	-1.62474600
C	5.06405300	1.89892000	1.43053800
H	3.44121800	0.89647400	2.44148100
C	5.46491300	2.47868600	0.22471900
H	4.95855400	2.98893100	-1.79715000
H	5.75036400	1.87567300	2.26807900
N	6.81156700	3.05252400	0.11984600
O	7.53279100	3.00147700	1.11049400
O	7.13920800	3.55149900	-0.95105100
O	-2.88394200	-2.89950100	-1.00540800
H	-3.19779000	-3.58592800	-1.60622800
H	-2.79805700	-2.03111100	-1.52551100
Zn	-2.66226900	0.41812300	-0.24613300

$\mathbf{T_{Zn}^{WA}}$

Charge = 0, Multiplicity = 1

Energy = -2430.3039523 hartree

O	-2.20831700	-2.14890500	-1.75005800
C	-2.66042100	2.30442900	-0.42467300
H	-3.21397000	3.18358600	-0.78452300
H	-1.59483100	2.54259000	-0.46724300
C	-5.24066200	0.41147400	-1.15348100
H	-5.47609000	1.07225900	-0.31413900
H	-6.16296000	0.30528500	-1.74276500
C	-4.14167900	1.05005400	-2.02684600
H	-3.98197400	0.42989800	-2.91709200
H	-4.49588200	2.03138600	-2.37707400
C	-4.54926700	-2.42443600	1.31642200
H	-4.99148600	-2.68164000	2.29017900
H	-4.58931000	-3.32559000	0.69456000
C	-3.04602800	2.02336600	1.04071400
H	-2.70801400	2.86884100	1.65917100
H	-4.13489300	1.96174400	1.15798800
C	-2.70002100	-1.37577400	2.69077300
H	-1.61889200	-1.52532700	2.76032600
H	-3.16701100	-1.83411900	3.57518300
C	-5.40567800	-1.32761100	0.65344600
H	-6.42425400	-1.71643600	0.50279300
H	-5.49259700	-0.46039200	1.31449400
C	-2.98698500	0.13684600	2.68701900
H	-4.06610800	0.32296000	2.73581200
H	-2.55333200	0.57495000	3.59890600
N	-3.13022100	-2.04808500	1.44524600
H	-2.54557000	-2.86613000	1.29667900
H	-2.64008400	-2.08059100	-2.61124000
N	-2.47489300	0.74393300	1.46061300
H	-1.45035200	0.79032200	1.46547400
N	-2.85889500	1.14671100	-1.32289000
H	-2.04780400	1.10484700	-1.95437700
N	-4.78069700	-0.87088700	-0.59477100

H	-4.84021000	-1.60575900	-1.29827600
C	4.26273000	-3.74299700	0.16138800
C	2.90235900	-3.77075200	0.47569700
C	2.09923900	-2.68422000	0.15554000
C	2.66801200	-1.57181300	-0.48467700
C	4.03844100	-1.55285100	-0.79115000
C	4.83998900	-2.63853600	-0.47016800
H	2.49027500	-4.64436600	0.96598800
H	1.04113800	-2.68687300	0.38065300
H	4.45061700	-0.67544500	-1.27783300
H	5.89847300	-2.64510600	-0.69964900
N	5.09939300	-4.89407100	0.50031300
O	6.29027900	-4.84611600	0.20649900
O	4.56614100	-5.84818700	1.06072300
O	1.97972400	-0.44774200	-0.81305800
P	0.34619600	-0.15186200	-0.83366100
O	-0.55115900	-0.90460900	0.17499300
O	-0.12673300	0.75707900	-1.94398900
O	0.61752900	1.14828800	0.43605400
C	1.32895000	2.27326600	0.33572200
C	1.95033300	2.73397200	-0.84839300
C	1.44578600	3.05601800	1.50955900
C	2.65160400	3.93099600	-0.84888600
H	1.85579400	2.15725300	-1.75858200
C	2.14406900	4.25124800	1.50885800
H	0.98047400	2.68694700	2.41880400
C	2.74577600	4.68736100	0.32344700
H	3.12740500	4.29577500	-1.75155200
H	2.23816200	4.85199000	2.40555500
N	3.47337200	5.94837400	0.31142000
O	3.52480600	6.59668900	1.35606400
O	3.99758900	6.30554800	-0.74206900
O	0.18253400	-1.73308400	-1.98388000
H	0.43556600	-1.46839900	-2.87965600
H	-1.05970800	-2.04307300	-1.91255000
Zn	-2.53367800	-0.74924100	-0.24809000

P_{Zn}^{WA}
Charge = 0, Multiplicity = 1
Energy = -2430.3602206 hartree

O	-2.99101200	-3.44943500	-1.61728400
C	-3.12987400	2.58891700	-0.83513500
H	-3.81680900	3.10202200	-1.52516800
H	-2.18502100	3.14346300	-0.83890100
C	-4.66234800	-0.40984100	-0.71936800
H	-5.21684400	0.23977000	-0.03413100
H	-5.40451400	-1.06014900	-1.20854300
C	-3.95302100	0.42097100	-1.80342200
H	-3.53529100	-0.25814500	-2.55241200
H	-4.69263300	1.05741200	-2.31233900
C	-2.97900300	-1.95791500	2.28752100
H	-3.35782700	-2.28872500	3.26663400
H	-2.35865200	-2.76230200	1.87919900
C	-3.72681500	2.62354600	0.58719100
H	-3.82235700	3.67358400	0.90534500
H	-4.73836500	2.20354000	0.58698200
C	-2.53259700	0.28311200	3.35940600
H	-1.61680700	0.78204800	3.68950300
H	-3.01739200	-0.14990000	4.24717100
C	-4.16214600	-1.71516600	1.33609300
H	-4.70998400	-2.65969500	1.20483400
H	-4.86387200	-1.00113200	1.78008600
C	-3.46439600	1.34787200	2.74343800
H	-4.45698800	0.93268600	2.53783700
H	-3.60722300	2.14729200	3.48914300
N	-2.11453100	-0.77075700	2.41020400
H	-1.16699500	-1.05781200	2.63961500
H	-3.48810300	-3.84591700	-2.34345600
N	-2.90096300	1.82404800	1.48634800
H	-1.98587500	2.25100800	1.64018400
N	-2.82418700	1.22225300	-1.29064900
H	-2.09508800	1.22346300	-2.00968000
N	-3.68245100	-1.17200900	0.06061500
H	-3.33515400	-1.94854300	-0.51902800
C	4.05964800	-2.73416400	0.54944600
C	2.97777900	-3.50149100	0.11686600
C	2.05072300	-2.93886300	-0.75321500
C	2.21756200	-1.61211900	-1.17128500
C	3.30763800	-0.85088400	-0.73347500
C	4.23699000	-1.41349000	0.13269700
H	2.87868100	-4.52445600	0.45860900
H	1.20808800	-3.51946100	-1.10950700
H	3.39835600	0.17577100	-1.06958500
H	5.08630700	-0.84587600	0.49249600
N	5.03433000	-3.33321800	1.47067400
O	5.98514800	-2.64584200	1.82368700
O	4.84025200	-4.48829800	1.83603400
O	1.36752600	-1.00554100	-2.06452600
P	-0.26823000	-1.12042900	-2.09315400
O	-0.78871100	-1.31617800	-0.68323300
O	-0.78611700	-0.03328200	-2.96964600
O	-0.27069700	1.17489100	1.12170600
C	0.73575700	1.89060000	0.69228900
C	0.93008200	2.22636500	-0.67888400
C	1.68627500	2.39783200	1.62650400
C	1.98791400	3.02653000	-1.08108600
H	0.24936000	1.82981500	-1.42621600
C	2.74152700	3.19593300	1.22729300
H	1.55576700	2.13921100	2.67369300
C	2.89207500	3.51557100	-0.13017700
H	2.13031200	3.28077200	-2.12512000
H	3.45845200	3.58210500	1.94255000
N	3.99181900	4.36188600	-0.55169500
O	4.76961200	4.77616500	0.30901400
O	4.09580800	4.62775000	-1.75003500
O	-0.52219900	-2.60164900	-2.78856500
H	-0.35720800	-2.59661100	-3.74337400
H	-2.08057200	-3.31200100	-1.94112700
Zn	-1.84799600	0.11130800	0.37900400

R_{Cu}^{WA}
Charge = 0, Multiplicity = 2

Energy = -2400.5278942 hartree

O	2.53887600	-0.06333700	-2.02420400
C	1.97437400	-3.12008900	1.10125000
H	2.14666800	-4.19974000	1.22995000
H	0.91261400	-2.93537900	1.29258400
C	4.73324600	-2.82277700	-0.71861600
H	5.03469400	-3.08232500	0.30343200
H	5.44298800	-3.33524100	-1.39023000
C	3.31601100	-3.36294100	-1.01899900
H	3.10375300	-3.20781000	-2.08187800
H	3.30430700	-4.44881600	-0.83626200
C	5.38605300	0.89074100	-0.12925800
H	6.14184800	1.43991700	0.45343800
H	5.39237400	1.30636400	-1.14233100
C	2.81266400	-2.35091900	2.13689800
H	2.46403900	-2.59444900	3.15114600
H	3.86431500	-2.64820800	2.07795600
C	3.87137200	1.28936000	1.84757200
H	2.95272500	1.86383100	2.00784500
H	4.70106600	1.85739900	2.29693500
C	5.80360300	-0.59613700	-0.20971900
H	6.77402300	-0.64527700	-0.73375600
H	5.97406800	-1.00721800	0.79318300
C	3.74750400	-0.06824500	2.55780800
H	4.70273400	-0.60150900	2.52659000
H	3.49517400	0.08179500	3.61716500
N	4.03005100	1.12929000	0.39637800
H	3.61904400	1.91670400	-0.11985800
H	1.70394600	-0.41997100	-2.36297300
N	2.74734800	-0.90281200	1.86768000
H	1.79959700	-0.55164500	2.03767600
N	2.24702700	-2.68041500	-0.27525100
H	1.37433100	-2.65271900	-0.81212900
N	4.75369100	-1.37553800	-0.83899700
H	4.53203900	-1.05856000	-1.78342300
C	-2.05310300	4.56410300	-0.03016300
C	-0.73581000	4.21822800	-0.33563700
C	-0.44585400	2.91686400	-0.73323700
C	-1.48618900	1.98389700	-0.81120900
C	-2.80601300	2.33959300	-0.51153100
C	-3.09590300	3.64085300	-0.11587800
H	0.04392300	4.96677700	-0.26606100
H	0.57191000	2.63498800	-0.98734000
H	-3.58950500	1.59438600	-0.59797700
H	-4.10647800	3.94808000	0.12337400
N	-2.35203700	5.94072700	0.39061800
O	-3.51752400	6.21965900	0.64858700
O	-1.41856400	6.73249400	0.46191400
O	-1.24221900	0.70314500	-1.26272000
P	-0.53823300	-0.48928500	-0.38026700
O	0.56348800	0.05803800	0.49157700
O	-0.27877300	-1.62624600	-1.31440400
O	-1.74288100	-0.85712500	0.70517200
C	-2.96925300	-1.40282100	0.44337700
C	-3.32166000	-1.98991600	-0.78199000
C	-3.88898200	-1.35835300	1.50316400
C	-4.59535200	-2.52763500	-0.93724700
H	-2.59912400	-2.03773100	-1.58776600
C	-5.15763200	-1.89829600	1.34775100
H	-3.58648600	-0.89462300	2.43603300
C	-5.49910900	-2.47888100	0.12411200
H	-4.89474500	-2.98814400	-1.87091400
H	-5.88397000	-1.87510400	2.15087300
N	-6.83854600	-3.05274000	-0.04632600
O	-7.60974300	-2.99642400	0.90588300
O	-7.11155700	-3.55758900	-1.12985300
O	2.74537800	2.51079300	-1.68207500
H	2.94284300	3.02546400	-2.47379100
H	2.59171800	1.54973200	-1.95834300
Cu	2.69250500	-0.52614900	-0.17302000

$\mathbf{T_{Cu}^{WA}}$
Charge = 0, Multiplicity = 2
Energy = -2400.4800777 hartree

O	-2.63944800	-1.71516400	-1.85176400
C	-2.52046600	2.53245200	-0.25076300
H	-3.03729400	3.49615500	-0.38717300
H	-1.45391000	2.70298800	-0.41694600
C	-5.28988800	0.89074900	-0.72435600
H	-5.39068200	1.52205100	0.16321300
H	-6.28951700	0.82341100	-1.17753200
C	-4.31451400	1.55549000	-1.71959800
H	-4.34062100	1.01183900	-2.67210600
H	-4.68546900	2.57419600	-1.92420500
C	-4.66899600	-2.14829400	1.53634400
H	-5.05934300	-2.36182900	2.54542100
H	-4.94393800	-2.99823700	0.90130800
C	-2.72995900	2.06962700	1.20556300
H	-2.22434300	2.77576500	1.87986800
H	-3.79247300	2.08007400	1.47191900
C	-2.51108700	-1.45522800	2.65306400
H	-1.45979200	-1.74020900	2.55202300
H	-2.87917700	-1.84678400	3.61638900
C	-5.38128500	-0.88780200	1.00488700
H	-6.45790900	-1.10024100	0.91809300
H	-5.27862300	-0.06331000	1.71604800
C	-2.60362000	0.08082300	2.69459600
H	-3.62495400	0.39293600	2.93662600
H	-1.96259400	0.44963000	3.50743000
N	-3.21204600	-2.04297500	1.50659900
H	-2.79201300	-2.93190000	1.25973500
H	-3.17199100	-1.39739600	-2.59295100
N	-2.23259400	0.69388100	1.40400000
H	-1.20922200	0.69231500	1.30048400
N	-2.94075000	1.53735000	-1.24254200
H	-2.23762400	1.48170400	-1.97971800
N	-4.80573400	-0.42967900	-0.27230300
H	-4.98886300	-1.13090400	-0.99028700
C	3.96363200	-3.82327900	0.12224300
C	2.57156700	-3.92332700	0.13370700
C	1.80075300	-2.86552600	-0.33381300
C	2.43944400	-1.70774700	-0.80387100

C	3.84025800	-1.61901800	-0.81479300
C	4.60780900	-2.67802800	-0.35217600
H	2.10830700	-4.83117100	0.50068000
H	0.72145700	-2.92804300	-0.35361900
H	4.30155400	-0.70915700	-1.18327600
H	5.68980900	-2.63082000	-0.34793400
N	4.76634500	-4.94355700	0.61822700
O	5.98790100	-4.83565900	0.58016700
O	4.17357500	-5.92999800	1.04589200
O	1.78057500	-0.62587900	-1.30667000
P	0.21327400	-0.15716300	-1.10257900
O	-0.65304900	-0.96433500	-0.11377200
O	-0.22781500	0.89612900	-2.07504500
O	0.70762000	0.94541400	0.29527300
C	1.51412400	2.01153700	0.26313000
C	2.07236600	2.56258700	-0.91318700
C	1.80813200	2.62370100	1.50443200
C	2.88562100	3.68469100	-0.84005500
H	1.84173400	2.11671200	-1.87150200
C	2.61675100	3.74526600	1.57708500
H	1.39389100	2.18109000	2.40564300
C	3.15334500	4.27448100	0.39866200
H	3.31487200	4.11906300	-1.73524900
H	2.84587700	4.21671900	2.52527300
N	3.99705500	5.46054000	0.46529300
O	4.19915800	5.96433900	1.56906800
O	4.46200100	5.90028700	-0.58417300
O	-0.26656300	-1.63320900	-2.40827700
H	-0.04689800	-1.35380300	-3.30758500
H	-1.41012000	-1.75682500	-2.22063900
Cu	-2.60002600	-0.55109700	-0.24509900

P_{Cu}^{WA}
Charge = 0, Multiplicity = 2
Energy = -2400.5337854 hartree

O	-2.81180200	-3.46647500	-1.72189000
C	-3.19513000	2.56385900	-0.71033300
H	-3.90232500	3.10402600	-1.36066200
H	-2.28259300	3.16708100	-0.64711800
C	-4.51003900	-0.50141800	-0.80278200
H	-5.12707600	0.07190500	-0.10471900
H	-5.17768600	-1.21228000	-1.31180400
C	-3.86714600	0.42583100	-1.84408800
H	-3.39887700	-0.17762200	-2.62656700
H	-4.65833800	1.02759900	-2.31985200
C	-2.89541900	-2.17116400	2.18957400
H	-3.33724400	-2.59031700	3.10837100
H	-2.21479200	-2.92118800	1.77406500
C	-3.81791000	2.45209800	0.69935400
H	-3.95998400	3.46878000	1.09918700
H	-4.81430900	1.99950600	0.64538900
C	-2.61861200	0.02345500	3.40611000
H	-1.74882900	0.55059400	3.80984900
H	-3.13476300	-0.45847400	4.25159400
C	-4.01333500	-1.89681200	1.17568600
H	-4.49735800	-2.84296900	0.89854000
H	-4.78245300	-1.26006100	1.62279700
C	-3.56747600	1.07199400	2.78154300
H	-4.53612300	0.62577900	2.52903300
H	-3.76882600	1.84203200	3.54474100
N	-2.10074400	-0.96584600	2.44973000
H	-1.14579800	-1.21009600	2.69101300
H	-3.31799500	-3.81137600	-2.46798700
N	-2.98602500	1.61619200	1.56044300
H	-2.08900900	2.06118800	1.75914900
N	-2.80659600	1.26778100	-1.27474100
H	-2.06403400	1.35852500	-1.96823500
N	-3.47987400	-1.21204200	-0.02108000
H	-3.06751600	-1.93521900	-0.62941300
C	4.17469300	-2.59167000	0.54062600
C	3.12113500	-3.38513400	0.08544600
C	2.18616800	-2.83879500	-0.78640000
C	2.31528400	-1.50121900	-1.18464300
C	3.37873700	-0.71466000	-0.72489700
C	4.31609900	-1.26069700	0.14306500
H	3.04985000	-4.41546400	0.41173500
H	1.36611600	-3.44024900	-1.16016100
H	3.44352900	0.31828000	-1.04783000
H	5.14474800	-0.67322500	0.51886000
N	5.15717700	-3.17318400	1.46401800
O	6.08221000	-2.46164000	1.83790100
O	4.99576800	-4.33902200	1.81085800
O	1.45585400	-0.90746600	-2.07487900
P	-0.17854800	-1.08160100	-2.12199600
O	-0.69925000	-1.32638700	-0.72210100
O	-0.72209400	-0.00361600	-2.99299600
O	-0.35466300	1.04035400	1.16798600
C	0.58563600	1.83942100	0.72310700
C	0.74347500	2.20358300	-0.64223900
C	1.49854200	2.40239700	1.66135100
C	1.73926000	3.08453700	-1.03796100
H	0.09844600	1.76299000	-1.39440000
C	2.49003100	3.28118100	1.26795500
H	1.39429800	2.11863900	2.70478300
C	2.60856300	3.62730200	-0.08562400
H	1.85839700	3.35704900	-2.08022000
H	3.18007600	3.70940000	1.98558900
N	3.64261500	4.55813200	-0.50165700
O	4.39319200	5.01451300	0.36098600
O	3.71807100	4.84704300	-1.69594800
O	-0.36326200	-2.56169900	-2.84916300
H	-0.18989800	-2.52859000	-3.80192000
H	-1.89919900	-3.32977400	-2.04252900
Cu	-1.83846900	0.01658400	0.40022500

R_{Co}^{WA}
Charge = 0, Multiplicity = 2
Energy = -2349.0600126 hartree

O	2.62158700	-0.36905700	-2.02882200
C	1.67010100	-3.11020000	1.20531500
H	1.74835900	-4.19118200	1.39725800
H	0.61918300	-2.83091600	1.33032000

C	4.54347400	-3.00248400	-0.44579100
H	4.79575300	-3.20936200	0.60067200
H	5.28689600	-3.53625800	-1.05954400
C	3.13908600	-3.54349700	-0.79450000
H	2.99958100	-3.47285700	-1.87774400
H	3.08154000	-4.60846000	-0.52207300
C	5.26352500	0.73106900	-0.12979800
H	6.00769000	1.31667900	0.43160500
H	5.29867800	1.06742100	-1.17063200
C	2.51710100	-2.34143800	2.23295500
H	2.09775700	-2.47393500	3.24076600
H	3.54321800	-2.72093500	2.25855000
C	3.70554700	1.24878000	1.77785800
H	2.78417200	1.83014500	1.88933800
H	4.52750600	1.83817000	2.21386700
C	5.65110300	-0.76353900	-0.08177900
H	6.60766700	-0.89469600	-0.61340800
H	5.81840500	-1.09631500	0.94950100
C	3.56001700	-0.07521200	2.54305500
H	4.50909300	-0.61953400	2.56286900
H	3.27080500	0.11460700	3.58656000
N	3.89061500	1.01108600	0.33704200
H	3.51719000	1.78272000	-0.22749300
H	1.79272800	-0.72285300	-2.38573300
N	2.56904500	-0.91769500	1.84221500
H	1.63630200	-0.50552200	1.95366500
N	2.04646400	-2.76471500	-0.17983900
H	1.21699600	-2.77539800	-0.77874800
N	4.56419300	-1.55681100	-0.64398400
H	4.38679900	-1.31661100	-1.62463400
C	-1.72145000	4.67022900	0.01789000
C	-0.46093300	4.26498400	-0.42571300
C	-0.28271100	2.96307800	-0.88488900
C	-1.37755100	2.09111600	-0.88343600
C	-2.64059000	2.50660200	-0.44866700
C	-2.81811800	3.80759900	0.00819300
H	0.36271100	4.96919200	-0.41126600
H	0.68937400	2.62916500	-1.24036900
H	-3.46763600	1.80594000	-0.47629300
H	-3.78160900	4.15932800	0.35429700
N	-1.89841900	6.04368700	0.51121700
O	-3.01562200	6.37635600	0.88975500
O	-0.91700900	6.77879900	0.52025000
O	-1.24905200	0.81062600	-1.38150500
P	-0.50708300	-0.42557200	-0.60025800
O	0.64512300	0.08239100	0.23211700
O	-0.32243800	-1.53725900	-1.57741600
O	-1.64626200	-0.81950500	0.55256800
C	-2.89881200	-1.32677200	0.34505900
C	-3.34847400	-1.83207400	-0.88467800
C	-3.74172300	-1.33081800	1.46735000
C	-4.64082300	-2.33766600	-0.98118200
H	-2.68583500	-1.84382000	-1.74131700
C	-5.02857500	-1.83990400	1.37135600
H	-3.36608600	-0.92840800	2.40184600
C	-5.46716900	-2.33947200	0.14284700
H	-5.01399000	-2.73497100	-1.91748200
H	-5.69614800	-1.85365000	2.22408600
N	-6.82608600	-2.88137500	0.03529800
O	-7.52411300	-2.87727600	1.04381500
O	-7.18792100	-3.30812200	-1.05554100
Co	2.65575300	-0.69543100	-0.14234600
O	2.78053300	2.22790300	-1.92804200
H	3.02326300	2.64648200	-2.76280900
H	2.65312300	1.23566800	-2.09822500

T_{Co}^{WA}
Charge = 0, Multiplicity = 2
Energy = -2349.0138412 hartree

O	2.89097900	0.93859300	-2.05810400
C	2.07446400	-2.77635500	0.35318500
H	2.41014400	-3.82507200	0.36193300
H	0.98481600	-2.77450800	0.27886300
C	5.01679300	-1.69904700	-0.50216000
H	5.07985400	-2.14266400	0.49537800
H	5.99275700	-1.85253000	-0.98336200
C	3.90653500	-2.38900800	-1.31911400
H	3.96585900	-2.05897000	-2.36383600
H	4.09828700	-3.47452800	-1.31879200
C	4.90799200	1.83767400	0.97726500
H	5.38156600	2.26338600	1.87629000
H	5.19817500	2.46776800	0.12939900
C	2.47601900	-2.10846300	1.68173300
H	1.90089400	-2.55854400	2.50333400
H	3.53577900	-2.26889600	1.90812400
C	2.80327000	1.63128400	2.35717800
H	1.77910100	2.00520200	2.27078700
H	3.30198700	2.18678600	3.16750500
C	5.46350500	0.41862200	0.75101900
H	6.53862200	0.47842300	0.52785900
H	5.35672900	-0.19108000	1.65209100
C	2.75616900	0.13991000	2.72901400
H	3.75536700	-0.22857000	2.98403200
H	2.13406500	0.01035500	3.62557500
N	3.44079500	1.86181200	1.05010900
H	3.08186800	2.71923900	0.64263400
H	3.05724100	0.25302600	-2.72152900
N	2.24815000	-0.65409600	1.59642300
H	1.24127000	-0.47988700	1.47762400
N	2.57496700	-2.04788200	-0.82264500
H	1.85470300	-2.03763300	-1.55204100
N	4.71702900	-0.25798300	-0.32994000
H	4.90626300	0.23351400	-1.20632900
C	-3.28784100	4.22449100	0.10295400
C	-1.90201500	4.17352400	-0.05769700
C	-1.30680700	3.01470600	-0.54097500
C	-2.11169100	1.90756300	-0.85522100
C	-3.50530200	1.97229400	-0.69372800
C	-4.09828200	3.13168800	-0.21512900
H	-1.30834800	5.04538000	0.18936000
H	-0.23857000	2.95694500	-0.69684100
H	-4.09783000	1.09851800	-0.94244800

H	-5.17044300	3.20158200	-0.07830500
N	-3.90360100	5.44941600	0.61659600
O	-5.12485300	5.47397800	0.73240300
O	-3.16439800	6.38673000	0.90558700
O	-1.62964800	0.73899100	-1.35709600
P	-0.12435200	0.06219100	-1.17392100
O	0.84973500	0.71943500	-0.15729800
O	0.16486700	-1.08407700	-2.10606300
O	-0.80045700	-0.93391100	0.24032500
C	-1.78398200	-1.83434900	0.22053900
C	-2.42447600	-2.30563600	-0.95019600
C	-2.19415400	-2.35694900	1.47143800
C	-3.42782000	-3.25979400	-0.86198200
H	-2.10933100	-1.93344600	-1.91589300
C	-3.19362800	-3.31066300	1.55917500
H	-1.70945800	-1.97924700	2.36704000
C	-3.80952600	-3.76117300	0.38647600
H	-3.92127000	-3.63080900	-1.75259100
H	-3.51150000	-3.71076300	2.51454900
N	-4.85624400	-4.76957100	0.46957000
O	-5.15946600	-5.19843200	1.58243200
O	-5.38407600	-5.14529200	-0.57559600
Co	2.71553500	0.13238500	-0.22375900
O	0.52271000	1.39303300	-2.43178200
H	0.21160700	1.17045000	-3.32108800
H	1.81946400	1.26314100	-2.29091500

$\mathbf{P_{Co}^{WA}}$

Charge = 0, Multiplicity = 2

Energy = -2349.0660169 hartree

O	-3.05400900	-3.21363600	-1.89045200
C	-3.03031600	2.72488700	-0.34909300
H	-3.74574600	3.35603700	-0.89965600
H	-2.09446100	3.28732500	-0.26170600
C	-4.51602000	-0.20694300	-0.74340900
H	-5.11045800	0.32279200	0.00655500
H	-5.20485300	-0.85966000	-1.29836500
C	-3.83787000	0.77357800	-1.70852700
H	-3.41209600	0.21935400	-2.54936800
H	-4.58993300	1.46940100	-2.11297600
C	-2.83455800	-2.28821600	1.94929200
H	-3.22279700	-2.79055100	2.84987300
H	-2.25419300	-3.01967800	1.37893000
C	-3.58841900	2.45820600	1.06535500
H	-3.65491700	3.41208500	1.60989100
H	-4.60443300	2.05309300	1.01593300
C	-2.29830200	-0.29455000	3.39339300
H	-1.36843400	0.14654300	3.76437400
H	-2.75661300	-0.85438300	4.22340500
C	-4.00578100	-1.80726500	1.08463400
H	-4.57353400	-2.67043600	0.71367500
H	-4.69417900	-1.18869900	1.66757100
C	-3.24486500	0.85300000	2.97764800
H	-4.25740200	0.48512400	2.78217000
H	-3.32301300	1.56421800	3.81444000
N	-1.92664700	-1.17694300	2.27414100
H	-0.97964500	-1.51887900	2.40628100
H	-3.58736300	-3.47246100	-2.65243600
N	-2.73758500	1.48143500	1.75413400
H	-1.81657000	1.88547100	1.94293600
N	-2.71357200	1.48497100	-1.07406000
H	-1.99830600	1.63690500	-1.78383000
N	-3.48641200	-1.00119300	-0.04305600
H	-3.11375000	-1.67781300	-0.72544600
C	3.98242500	-2.74849200	0.45316200
C	2.88938100	-3.47904400	-0.01432300
C	1.97634300	-2.86727000	-0.86576400
C	2.16657000	-1.52742700	-1.23157600
C	3.26958900	-0.80463500	-0.76006500
C	4.18492800	-1.41616800	0.08727600
H	2.77149300	-4.51299900	0.28612900
H	1.12662200	-3.41957500	-1.24894700
H	3.38097200	0.23191200	-1.05770300
H	5.04296100	-0.87830400	0.47127200
N	4.94066300	-3.39886200	1.35537600
O	5.90238200	-2.74291400	1.73840900
O	4.72378800	-4.56303600	1.67717700
O	1.33293700	-0.87031900	-2.09992800
P	-0.30757300	-0.98153800	-2.16033800
O	-0.84439400	-1.26599300	-0.77393300
O	-0.80635800	0.14727100	-2.99212400
O	-0.25021700	0.93507600	1.12501100
C	0.66707600	1.76657000	0.69930500
C	0.78607600	2.21422900	-0.64660200
C	1.60896700	2.27709400	1.64165400
C	1.76683400	3.12306100	-1.01558200
H	0.13057700	1.80842500	-1.40844100
C	2.58498000	3.18316900	1.27452400
H	1.53857100	1.92785900	2.66814900
C	2.66173800	3.61367100	-0.05836100
H	1.85679100	3.45662100	-2.04298600
H	3.29458100	3.57012600	1.99656500
N	3.67777400	4.57343500	-0.44679200
O	4.45264000	4.98131000	0.41942500
O	3.71622200	4.93628700	-1.62305700
Co	-1.85823200	0.07009300	0.43381100
O	-0.53838700	-2.42724100	-2.94297000
H	-0.34911300	-2.36618600	-3.89131400
H	-2.13392500	-3.13216700	-2.20911300

$\mathbf{R_{Ce}}$

Charge = 2, Multiplicity = 1

Energy = -2601.4327516 hartree

O	-2.82390200	1.73465600	0.25136100
C	-1.91934900	-2.68774700	2.31467200

H	-2.26261100	-3.26950800	3.18002300
H	-0.84582800	-2.51686400	2.44155800
C	-4.94593900	-1.58510100	1.50229600
H	-4.89079100	-2.64405600	1.24319500
H	-5.98107300	-1.39408300	1.81313800
C	-4.01144200	-1.25886100	2.66813400
H	-4.19334900	-0.23509500	3.01397500
H	-4.23738400	-1.92583000	3.51030000
C	-4.52533100	-0.79173500	-2.21526200
H	-4.93738300	-1.28969200	-3.10260500
H	-4.74368800	0.27705700	-2.31685200
C	-2.16867800	-3.49993200	1.04231500
H	-1.60051200	-4.43667200	1.10502500
H	-3.22004200	-3.77969800	0.95872600
C	-2.44631600	-2.24092800	-2.56459000
H	-1.41970200	-2.03627300	-2.88185600
H	-2.98017500	-2.66200400	-3.42642300
C	-5.21085500	-1.34354700	-0.96466300
H	-6.28190300	-1.10919200	-1.01229800
H	-5.13281900	-2.43161000	-0.92626400
C	-2.44296200	-3.27099000	-1.43357700
H	-3.45933500	-3.57416300	-1.17713700
H	-1.92546700	-4.17421300	-1.78067500
N	-3.03609100	-0.92040100	-2.17855300
H	-2.66765400	-0.24742400	-2.85472500
H	-2.88409400	2.70072800	0.31682500
N	-1.80413600	-2.74305800	-0.19132100
H	-0.78867300	-2.80035300	-0.31165900
N	-2.56126900	-1.33478200	2.30682100
H	-2.05936200	-0.79017800	3.01229700
N	-4.59910700	-0.80256100	0.28254400
H	-4.94626500	0.15179200	0.41247000
C	1.02287000	5.10259100	0.05184400
C	1.05138000	4.55488000	-1.22907000
C	1.34050700	3.19806700	-1.37324400
C	1.58817200	2.44917000	-0.22530000
C	1.58360700	2.99603400	1.05582900
C	1.29338900	4.35315900	1.19490800
H	0.85877600	5.18711800	-2.08777800
H	1.39661800	2.73655600	-2.35333400
H	1.82424200	2.38291300	1.91759900
H	1.28416000	4.83357000	2.16614400
N	0.68520900	6.54291400	0.20578900
O	0.65806800	6.98503800	1.34183200
O	0.44675000	7.16600700	-0.81462900
O	1.92376700	1.07993600	-0.37924300
P	0.89970300	-0.11415900	-0.31281100
O	-0.22631200	-0.06862200	-1.37723700
O	0.08461700	-0.17616500	1.01055800
O	1.76562200	-1.40763300	-0.53869100
C	3.16097500	-1.60803500	-0.31920800
C	3.63970300	-1.70075500	0.98359500
C	3.96653200	-1.76296800	-1.44109500
C	4.99840800	-1.95056800	1.17119400
H	2.97328500	-1.58909700	1.83292800
C	5.32405000	-2.01686900	-1.24886100
H	3.54468200	-1.69205200	-2.43772900
C	5.81281800	-2.10397300	0.05217200
H	5.42865900	-2.03154000	2.16219800
H	5.99951900	-2.14624000	-2.08609200
N	7.26111600	-2.37410500	0.25608500
O	7.94373900	-2.50720700	-0.74463000
O	7.65235900	-2.44118700	1.40910700
Ce	-2.05231600	-0.14254300	0.07227100

T1$^{DA}_{Ce}$
Charge = 2, Multiplicity = 1
Energy = -2601.3452152 hartree

O	0.38794500	0.94085300	-1.24132700
C	3.61695000	-2.71141700	1.90681000
H	4.33025300	-3.50764600	2.15469300
H	2.65102300	-3.00143100	2.33556100
C	4.50564200	-2.16309200	-1.79042400
H	5.44014500	-2.28562600	-2.35250500
H	3.74094300	-2.75726400	-2.30293400
C	4.70207800	-2.66964800	-0.36073500
H	5.08508400	-3.69696900	-0.38781100
H	5.45328600	-2.06851800	0.15897100
C	4.56475400	1.63746600	-1.22911700
H	5.38752600	2.35542000	-1.11969200
H	3.92106300	2.00625400	-2.03566000
C	4.09301300	-1.39263200	2.51811100
H	4.24637600	-1.52482000	3.59614900
H	5.05995400	-1.10218300	2.09789300
C	4.52711000	1.54504300	1.28238300
H	4.95585400	2.53027200	1.50257800
H	5.37040100	0.86555400	1.13211600
C	5.13513200	0.26584400	-1.59470300
H	5.76375500	0.36157700	-2.48846800
H	5.78315200	-0.10584100	-0.79624500
C	3.67446100	1.08569300	2.46661600
H	4.27250200	1.13109400	3.38581400
H	2.82388300	1.76265900	2.60667000
N	3.73261500	1.58807300	0.01605700
H	3.17484800	2.44573100	0.03656200
H	0.00335700	1.82351200	-1.09880500
N	3.12217700	-0.28774200	2.25660700
H	2.33239100	-0.40718700	2.89730900
N	3.43215200	-2.60313300	0.42514400
H	2.84744900	-3.39057400	0.13068300
N	4.05211000	-0.73662800	-1.81908200
H	3.65085000	-0.56287200	-2.74516200
C	-2.52288200	4.47912400	0.04810400
C	-2.32426800	3.62957500	1.13572500
C	-2.12291300	2.27121500	0.90118500
C	-2.12732000	1.80656600	-0.41808700
C	-2.34702900	2.65826300	-1.50465000
C	-2.54789800	4.01835000	-1.26573000
H	-2.33984700	4.03586400	2.14018400
H	-1.99112200	1.57496300	1.72244000
H	-2.38474700	2.25438300	-2.51121600
H	-2.72935400	4.71475400	-2.07597700

N	-2.70572100	5.93233000	0.30250600
O	-2.89419600	6.64439100	-0.66835500
O	-2.63843900	6.30144400	1.46354100
O	-1.98481400	0.44289300	-0.66347000
P	-0.65109100	-0.48568000	-0.60927100
O	0.05170700	-0.40968800	0.82312800
O	0.64970500	-1.29163500	-1.34595700
O	-1.50860300	-1.84734300	-0.75974000
C	-2.84447200	-2.09053600	-0.42297600
C	-3.72095500	-2.41096100	-1.45945400
C	-3.23772100	-2.10304400	0.91680400
C	-5.03711900	-2.74198600	-1.14862600
H	-3.37095600	-2.40646500	-2.48602900
C	-4.55383200	-2.43181400	1.22667800
H	-2.52444100	-1.87033800	1.70104500
C	-5.43084100	-2.74302100	0.18830400
H	-5.75449800	-2.99758700	-1.91919200
H	-4.90831500	-2.45669300	2.25002700
N	-6.83482700	-3.08997400	0.52206400
O	-7.14278000	-3.07653200	1.70322900
O	-7.57568800	-3.36032100	-0.40804500
Ce	2.08820600	-0.45589200	-0.13645400

$T1^{CA}_{Ce}$

Charge = 2, Multiplicity = 1

Energy = -2601.3789927 hartree

O	-3.32280000	1.50906800	0.00253300
C	-1.95074400	-2.49184200	2.58810700
H	-2.33798200	-2.87564800	3.54074400
H	-0.86041700	-2.44251700	2.67526300
C	-4.81385400	-1.15326400	1.56166500
H	-4.98760800	-2.23684600	1.54871800
H	-5.79187300	-0.69859500	1.78580800
C	-3.84574300	-0.77024000	2.68539400
H	-3.88540400	0.30990200	2.85917200
H	-4.14916800	-1.26069900	3.61872200
C	-4.38158500	-1.11463200	-2.21400700
H	-4.86962000	-1.72491900	-2.98399600
H	-4.47819400	-0.06836100	-2.52156200
C	-2.34321900	-3.45078700	1.46614900
H	-1.91393200	-4.43922100	1.67100900
H	-3.42584600	-3.57858500	1.42712800
C	-2.47736400	-2.82709500	-2.30472600
H	-1.43253300	-2.80183000	-2.63269200
H	-3.05333300	-3.32851500	-3.09310800
C	-5.07677400	-1.32816100	-0.86600500
H	-6.08939300	-0.89971500	-0.93380000
H	-5.22156000	-2.39815200	-0.66683100
C	-2.60338600	-3.62447800	-1.00805100
H	-3.65088300	-3.73728000	-0.72493900
H	-2.20581500	-4.63454300	-1.16580300
N	-2.91162700	-1.40350800	-2.16426600
H	-2.48180600	-0.90139700	-2.94483000
H	-3.55679000	2.44716100	-0.03379200
N	-1.90372600	-2.94944200	0.12791800
H	-0.90186600	-3.14856100	0.03801900
N	-2.41869200	-1.09068300	2.35928200
H	-1.83631500	-0.50014000	2.95753300
N	-4.34250600	-0.70603600	0.24408300
H	-4.12466600	0.69612900	0.13236900
C	0.63559700	5.05551400	0.01196600
C	0.46913800	4.45640700	-1.23476800
C	0.88713500	3.13661100	-1.40729500
C	1.45016700	2.47340000	-0.31815400
C	1.64100600	3.07535000	0.92285800
C	1.22467400	4.39613800	1.08889300
H	0.03407300	5.02382100	-2.04902500
H	0.80972800	2.64539100	-2.37160700
H	2.12165400	2.53244600	1.72954200
H	1.35536000	4.91640200	2.03047300
N	0.15950300	6.45199800	0.20148800
O	0.34529800	6.95523800	1.29561900
O	-0.39499300	6.97857000	-0.74882000
O	1.90851400	1.14306700	-0.49609800
P	0.99103900	-0.13410400	-0.41971300
O	-0.14681700	-0.17975900	-1.47889600
O	0.17461200	-0.23523600	0.90332100
O	1.95356200	-1.35010500	-0.64474500
C	3.35292000	-1.45179200	-0.37883600
C	3.78790000	-1.55798500	0.93799500
C	4.20752100	-1.50218300	-1.47327000
C	5.15412800	-1.70993100	1.17050600
H	3.08317100	-1.53321700	1.76304100
C	5.57277700	-1.65817200	-1.23579000
H	3.81809400	-1.42741600	-2.48268600
C	6.01860400	-1.75658400	0.07981500
H	5.55261200	-1.79674500	2.17421400
H	6.28658400	-1.70386800	-2.04966000
N	7.47541000	-1.92044700	0.33196600
O	8.20297600	-1.96283600	-0.64455100
O	7.82751000	-1.99866600	1.49688900
Ce	-1.94683900	-0.36462900	-0.04520200

$I1^{CA}_{Ce}$

Charge = 2, Multiplicity = 1

Energy = -2601.4082507 hartree

O	-2.14030500	1.71861600	1.13819000
C	-1.36561000	-3.79945100	1.33926700
H	-1.58030400	-4.62295400	2.03254600
H	-0.28169500	-3.78742800	1.17862300
C	-4.21256800	-2.16575200	1.79932700
H	-4.56357200	-3.11797300	1.37368100
H	-5.04696100	-1.79159700	2.41262600
C	-3.00271400	-2.38758600	2.71605600
H	-2.89499000	-1.53446200	3.39575900
H	-3.16099500	-3.27626100	3.33942700
C	-4.64460000	-0.21992300	-1.43858800
H	-5.35542300	-0.38564200	-2.25771700
H	-4.71364100	0.83860200	-1.16442800
C	-2.08625900	-4.05157300	0.01655600
H	-1.72202800	-4.99253800	-0.41505700
H	-3.15798100	-4.17526200	0.18179900

C	-2.96704900	-1.60928200	-2.78935300
H	-2.00921200	-1.40332400	-3.27646200
H	-3.73232500	-1.67506000	-3.57369800
C	-5.02180300	-1.09196900	-0.23317700
H	-5.89903100	-0.64542400	0.26012000
H	-5.34580100	-2.09189800	-0.55947900
C	-2.89862500	-2.94326400	-2.04998900
H	-3.86916700	-3.19678600	-1.61957300
H	-2.65530600	-3.73437300	-2.77062500
N	-3.23782100	-0.44379200	-1.89282000
H	-2.95480900	0.38353800	-2.42153100
H	-1.38376100	2.33952500	1.20103000
N	-1.90755300	-2.91915100	-0.93463900
H	-0.97292100	-2.98376100	-1.34869100
N	-1.71403400	-2.48512400	1.96052300
H	-0.96329600	-2.27536100	2.62169500
N	-3.91729000	-1.22329400	0.71894700
H	-2.91603900	2.15355200	1.52614100
C	-0.09683800	4.95074700	0.11202500
C	0.12838200	4.58758200	-1.21262400
C	0.73394600	3.35802000	-1.48234300
C	1.10288300	2.54147900	-0.41575000
C	0.91791200	2.92452500	0.91845800
C	0.30050500	4.14976400	1.18476900
H	-0.16692100	5.25913900	-2.01073000
H	0.93294200	3.04224300	-2.50098200
H	1.29023500	2.29941500	1.72341700
H	0.15788000	4.50485600	2.19970300
N	-0.79068500	6.23419100	0.40107700
O	-1.09610100	6.43942300	1.56526800
O	-1.01829200	6.96514000	-0.54474500
O	1.72750900	1.31894500	-0.70493000
P	1.00961000	-0.09329700	-0.53504500
O	-0.21305100	-0.28145100	-1.45765900
O	0.41206100	-0.26933400	0.88655300
O	2.11660900	-1.13856300	-0.90933700
C	3.49525600	-1.13361600	-0.54804800
C	3.86079400	-1.16742400	0.79441500
C	4.41548300	-1.15366400	-1.58919000
C	5.21857400	-1.20725400	1.10792200
H	3.11056200	-1.17102700	1.57857800
C	5.77222300	-1.20003700	-1.27059700
H	4.07950600	-1.13725600	-2.62009100
C	6.14697400	-1.22263600	0.07047800
H	5.56306200	-1.23255500	2.13475900
H	6.53319000	-1.21722500	-2.04168100
N	7.59355500	-1.26768300	0.41049500
O	8.37979800	-1.28476900	-0.52036100
O	7.88129700	-1.28303600	1.59586300
Ce	-1.84182700	-0.59777900	0.24539700

T2$_{Ce}^{CA}$
Charge = 2, Multiplicity = 1
Energy = -2601.3026733 hartree

O	-0.16993500	1.69206900	0.17263700
C	-4.26256200	-1.29947600	2.61349000
H	-4.56860500	-1.01804900	3.62964600
H	-4.19544300	-2.39438500	2.59653100
C	-3.04553500	1.69898200	1.77571600
H	-4.13091000	1.83859800	1.84211800
H	-2.59825300	2.65480300	2.08500600
C	-2.57011400	0.62123100	2.75303200
H	-1.48159300	0.65771500	2.83381400
H	-2.99189000	0.81184600	3.74770400
C	-3.36255200	1.66897200	-2.02219500
H	-4.05786100	2.22513600	-2.66326300
H	-2.35590700	1.80257200	-2.42462600
C	-5.32380700	-0.82350500	1.62417800
H	-6.28453300	-1.29057400	1.87324400
H	-5.46636900	0.25464800	1.71165800
C	-5.06574600	-0.24082500	-2.14768100
H	-5.08215000	-1.23915600	-2.60204200
H	-5.65418800	0.41387100	-2.80380800
C	-3.42771300	2.22111900	-0.59451300
H	-3.02859000	3.24630600	-0.60963500
H	-4.46801500	2.31376100	-0.26060200
C	-5.71976900	-0.27996000	-0.76843700
H	-5.78696300	0.72694800	-0.35409100
H	-6.74729000	-0.65119200	-0.86743500
N	-3.63865100	0.19562400	-2.10711600
H	-3.20881000	-0.11211900	-2.98253700
H	0.19376300	2.48445900	-0.25130400
N	-4.94274800	-1.11056100	0.20485500
H	-5.17902300	-2.09119400	0.02126600
N	-2.89944300	-0.77504700	2.30640700
H	-2.23184300	-1.37926900	2.79085400
N	-2.67913500	1.38650800	0.37409700
H	-1.37251100	1.64271400	0.25069700
C	3.68088900	4.22877900	0.07616100
C	3.18006500	3.92108200	-1.18602800
C	2.56926600	2.68179000	-1.38195100
C	2.47894900	1.80476800	-0.30129100
C	2.99203900	2.10584700	0.95817400
C	3.60652200	3.34182700	1.14833300
H	3.28198500	4.63689600	-1.99310500
H	2.20258500	2.38800400	-2.36070100
H	2.92069400	1.38774400	1.76763300
H	4.02485900	3.62457000	2.10706900
N	4.31730200	5.55619900	0.28837200
O	4.75407100	5.78647700	1.40275100
O	4.34427400	6.31342500	-0.66681000
O	1.92574500	0.53122800	-0.52513400
P	0.45909800	-0.08374700	-0.44120100
O	-0.70088500	-0.04530500	-1.52392000
O	-0.32172500	-0.62577800	0.83504700
O	1.02041100	-1.58073600	-0.98104200
C	2.13184000	-2.28351300	-0.61179800
C	2.67280100	-2.23301300	0.68671900
C	2.68572000	-3.12388100	-1.59646500
C	3.77564800	-3.01688800	0.99278500
H	2.22547300	-1.60206900	1.44678400
C	3.78721400	-3.90906400	-1.28813900

H	2.24746100	-3.13429100	-2.58853600
C	4.31756200	-3.84225400	0.00213600
H	4.22337600	-3.00844700	1.97946200
H	4.24281000	-4.56360700	-2.02171100
N	5.49508000	-4.67817200	0.33904600
O	5.86801600	-5.47357400	-0.50610000
O	5.99790700	-4.50475800	1.43765800
Ce	-2.30125700	-0.85497200	-0.18406500

I^{DA}_{Ce}/$I2^{CA}_{Ce}$
Charge = 2, Multiplicity = 1
Energy = -2601.3357662 hartree

O	0.67098900	-2.09040300	-0.37527600
C	-3.41903200	1.03818900	2.31190500
H	-3.89326700	1.91593000	2.76824500
H	-2.53113400	0.79651200	2.90470200
C	-3.53696300	2.10337100	-1.37058400
H	-4.23963600	2.71892300	-1.94632000
H	-2.53445100	2.51202400	-1.53638900
C	-3.89353600	2.16698000	0.11490900
H	-3.88881800	3.21224800	0.44714700
H	-4.90552300	1.78869500	0.28668300
C	-4.83702800	-1.39450800	-2.31259100
H	-5.83903500	-1.75499900	-2.57853400
H	-4.17031700	-1.67505900	-3.13656300
C	-4.39729300	-0.13676000	2.32029800
H	-4.75298800	-0.31380300	3.34271400
H	-5.27976000	0.09216100	1.71605500
C	-5.35719300	-2.17055100	0.01328500
H	-6.08857000	-2.95643400	-0.21081500
H	-5.91172100	-1.22862300	0.03973100
C	-4.87652100	0.12749200	-2.16033100
H	-5.29581700	0.56918000	-3.07281200
H	-5.54135100	0.41509400	-1.34147200
C	-4.71627800	-2.44552600	1.37426200
H	-5.50385300	-2.56528400	2.12946600
H	-4.15804400	-3.38877900	1.34744900
N	-4.33515600	-2.07768100	-1.07692000
H	-4.09767400	-3.03619200	-1.34570200
H	1.31575700	-2.70065200	0.01774700
N	-3.75929600	-1.36748200	1.76446700
H	-3.13631500	-1.74087700	2.48595900
N	-2.94862200	1.34854500	0.92670500
H	-2.06074700	1.85433900	1.00826100
N	-3.52472900	0.69353800	-1.87625600
H	-2.99669400	0.69441700	-2.75359000
C	5.33119300	-2.99987200	-0.04788300
C	4.68063500	-2.96933600	1.18265300
C	3.60610700	-2.09590300	1.35117300
C	3.22233600	-1.29005700	0.27867300
C	3.88010300	-1.31520800	-0.95022100
C	4.95482400	-2.18640500	-1.11483100
H	5.02163500	-3.61091700	1.98642100
H	3.09623400	-2.01683100	2.30646600
H	3.56646600	-0.66109000	-1.75655500
H	5.50055300	-2.24212700	-2.04917800
N	6.47042300	-3.93781400	-0.23049600
O	7.01920800	-3.93457100	-1.31909400
O	6.76381900	-4.64152700	0.72107200
O	2.18594100	-0.36242700	0.48432400
P	0.60112500	-0.51482900	0.22884800
O	-0.58521600	-0.88143200	1.27406100
O	-0.33606100	-0.13723000	-1.07541000
O	0.46256100	1.15630100	0.77898100
C	1.27320600	2.20560500	0.48854700
C	1.88027700	2.38217400	-0.77253900
C	1.42987700	3.17845200	1.49751200
C	2.63196200	3.52188600	-1.01799800
H	1.74636900	1.64099700	-1.55266600
C	2.18162800	4.31822000	1.25155000
H	0.97385100	3.00816600	2.46724200
C	2.77170200	4.47473800	-0.00457800
H	3.10832000	3.69322900	-1.97614100
H	2.32661500	5.07959200	2.00883400
N	3.56794200	5.69483300	-0.27649200
O	3.60930500	6.53604100	0.60590600
O	4.11675300	5.76580900	-1.36441300
Ce	-2.15964300	-0.77961000	-0.21545900

$T2^{DA}_{Ce}$/$T3^{CA}_{Ce}$
Charge = 2, Multiplicity = 1
Energy = -2601.3267561 hartree

C	-3.09952000	1.15269800	2.27657900
H	-3.34189100	2.13339800	2.70542600
H	-2.29045400	0.72380400	2.87644400
C	-2.94289000	2.10356100	-1.42735500
H	-3.45350100	2.86121100	-2.03479600
H	-1.86616300	2.22456800	-1.57953300
C	-3.28581300	2.30061400	0.04929900
H	-3.01056900	3.31847400	0.35354000
H	-4.36305500	2.20464100	0.21631700
C	-5.09652100	-0.96171800	-2.27909300
H	-6.15983500	-1.06809800	-2.52953700
H	-4.52982300	-1.43257200	-3.09103700
C	-4.33377900	0.25317900	2.32484600
H	-4.71968400	0.19613900	3.34984800
H	-5.13462500	0.67113900	1.70784600
C	-5.77645200	-1.51406100	0.07103200
H	-6.68263100	-2.09425600	-0.14208000
H	-6.07497900	-0.46252200	0.08276500
C	-4.74361800	0.52288000	-2.18106700
H	-5.02613800	1.02604300	-3.11402700
H	-5.31782600	1.00041200	-1.38245700
C	-5.21852300	-1.92262200	1.43506400
H	-6.00560300	-1.82667800	2.19396200
H	-4.91765400	-2.97704200	1.42151700
N	-4.76738900	-1.69632400	-1.01743900
H	-4.75407400	-2.69354900	-1.24711000
N	-4.01755000	-1.11434400	1.80703400
H	-3.52488400	-1.61319400	2.55323900
N	-2.58802700	1.29112000	0.88572800
H	-1.58135300	1.50797600	0.92621700

N	-3.29429000	0.72385600	-1.89057100
H	-2.77402100	0.55893200	-2.75688400
C	5.35265900	-2.95869000	-0.05993300
C	4.67344100	-2.97298600	1.15509800
C	3.55306300	-2.15483700	1.30710300
C	3.15606300	-1.35961800	0.23200100
C	3.84188700	-1.33728800	-0.98050700
C	4.96231600	-2.15248400	-1.12737700
H	5.02724000	-3.60538500	1.96065700
H	3.01948200	-2.10936600	2.25166800
H	3.51310700	-0.69049500	-1.78667100
H	5.53181200	-2.17126600	-2.04895900
N	6.54009700	-3.83884600	-0.22507700
O	7.11401000	-3.79512400	-1.29961500
O	6.84370400	-4.53955100	0.72535500
O	2.06644200	-0.48550800	0.41617100
P	0.50431100	-0.73176600	0.15075100
O	-0.61993500	-1.05551900	1.23219400
O	-0.39696600	-0.39581400	-1.12432700
O	0.34340800	1.23975200	0.76739800
C	1.22037200	2.21515900	0.50660200
C	1.68102500	2.47037000	-0.81086500
C	1.63923800	3.04625000	1.57517200
C	2.53006500	3.53766000	-1.05098500
H	1.35536000	1.82960300	-1.62429900
C	2.48947000	4.11315400	1.33363000
H	1.29286700	2.82240500	2.57871100
C	2.92129800	4.34571000	0.02361200
H	2.89580400	3.76552900	-2.04531900
H	2.83108500	4.76346300	2.13036500
N	3.82621500	5.49071600	-0.23960300
O	4.12265900	6.19116900	0.71362200
O	4.20096500	5.64404600	-1.39114000
Ce	-2.32430400	-0.98878300	-0.19286300
O	0.68770000	-2.30880200	-0.35741900
H	1.37498800	-2.84943700	0.06343600

P_{Ce}

Charge = 2, Multiplicity = 1

Energy = -2601.450694 hartree

O	-1.00207800	-3.29969200	-0.79735900
C	4.57536100	0.16945200	-2.01243500
H	5.57860100	0.55829500	-2.23016300
H	3.93421300	0.44771300	-2.85709900
C	4.26196700	1.26028500	1.65231300
H	5.01004300	1.40990500	2.44156600
H	3.70201600	2.19543400	1.55257800
C	4.97087200	0.92839900	0.34008600
H	5.71913100	1.70178500	0.12475900
H	5.51447800	-0.01623400	0.42685600
C	2.97566900	-2.20458800	2.61880000
H	3.44741200	-3.06493500	3.11100500
H	2.05324800	-1.98640300	3.16899700
C	4.62846300	-1.35060900	-1.87276200
H	5.04192000	-1.79050300	-2.78888600
H	5.29914000	-1.63805900	-1.05829500

C	3.60432000	-3.31338300	0.45606700
H	3.64600900	-4.35045100	0.81216800
H	4.58776400	-2.87493500	0.64887600
C	3.91749600	-1.00361300	2.66505700
H	4.20434000	-0.79562900	3.70317000
H	4.84119900	-1.22368200	2.12323800
C	3.29201800	-3.29570800	-1.04003400
H	4.02011900	-3.91745800	-1.57660500
H	2.30256600	-3.72869700	-1.22346500
N	2.58887300	-2.53392700	1.21698000
H	1.72833400	-3.08576000	1.25717300
H	-1.60296700	-3.68222200	-1.45790000
N	3.27766700	-1.90313200	-1.57918500
H	2.74043200	-1.91929600	-2.44981400
N	4.00451400	0.80442700	-0.78886200
H	3.70679600	1.75006900	-1.03619200
N	3.28665900	0.19570300	2.03932600
H	2.65459100	0.60605600	2.73119300
C	-6.01843200	-1.78856000	0.15564200
C	-5.76721500	-1.53009400	-1.18965100
C	-4.45612500	-1.27995500	-1.59247300
C	-3.45289400	-1.29701700	-0.62870100
C	-3.69856800	-1.54166100	0.72054400
C	-5.01075900	-1.79839500	1.11609900
H	-6.58647200	-1.52721000	-1.89877500
H	-4.21626000	-1.06455900	-2.62817000
H	-2.89593000	-1.52515100	1.45079500
H	-5.26039700	-1.99826200	2.15121400
N	-7.41517600	-2.06105800	0.58547100
O	-7.59460900	-2.27871400	1.77224000
O	-8.27226300	-2.04728900	-0.28067300
O	-2.14109900	-1.00013100	-1.08915700
P	-0.79250800	-1.72810300	-0.72540700
O	0.30808400	-1.21688200	-1.68143800
O	-0.22466200	-1.41293100	0.67694300
O	1.08663700	1.58361200	0.08848300
C	0.32777200	2.69760700	0.05960400
C	-0.65008700	2.90063500	1.05648400
C	0.52025900	3.64850700	-0.96452000
C	-1.42417000	4.05208400	1.03200900
H	-0.78811800	2.15512200	1.83347400
C	-0.25713500	4.79782600	-0.98831100
H	1.26761500	3.47174100	-1.73190600
C	-1.21576400	4.98141600	0.01049000
H	-2.18327000	4.24500000	1.78102700
H	-0.13956700	5.55004700	-1.75953600
N	-2.04906700	6.21239000	-0.01673800
O	-1.82666600	7.00654900	-0.91421500
O	-2.88758300	6.32737900	0.86046500
Ce	1.76555100	-0.38744800	-0.04460800

R_{Ce}^{WA}

Charge = 2, Multiplicity = 1

Energy = -2677.9101752 hartree

O	2.68650000	1.06984700	-0.07802200
C	0.39054000	-3.83873400	-0.23500000

H	0.27614200	-4.85906800	-0.62371300
H	-0.61140300	-3.39923300	-0.18700500
C	3.34376400	-2.66645000	-2.36216300
H	4.20027000	-3.18707300	-2.80981700
H	2.84698500	-2.11287700	-3.16720100
C	2.37964600	-3.68408700	-1.75422400
H	2.07052300	-4.40247500	-2.52414200
H	2.87224700	-4.26094500	-0.96673300
C	5.00573100	-1.29430400	0.80487500
H	5.83249500	-1.66773300	1.42322900
H	5.25685400	-0.27324400	0.50297000
C	1.01527200	-3.88875500	1.15846100
H	0.41171700	-4.53398800	1.80962500
H	2.01262100	-4.33533300	1.11315400
C	3.50961000	-2.38977300	2.48915900
H	4.15937700	-2.34214500	3.37216700
H	3.78440400	-3.29842400	1.94617100
C	4.85214700	-2.18346200	-0.42713500
H	5.81333700	-2.25440500	-0.95186600
H	4.58177200	-3.20064300	-0.13112000
C	2.04649900	-2.44921500	2.92341900
H	1.89133700	-3.30167400	3.59768800
H	1.78166900	-1.54600400	3.48572400
N	3.74100200	-1.22437800	1.59300400
H	3.79569400	-0.38554200	2.17497800
H	3.05510600	2.03159200	-0.11797700
N	1.14357200	-2.52409600	1.74142900
H	0.21294400	-2.20924600	2.02495700
N	1.19972900	-3.00073600	-1.16374600
H	0.59120400	-2.69179800	-1.92558800
N	3.79190600	-1.67166600	-1.34362400
H	4.18160700	-0.86765700	-1.84074900
C	0.31569400	4.87399800	0.01221300
C	-0.70390200	4.50160200	0.88689600
C	-1.42956400	3.34492200	0.60643400
C	-1.12468700	2.63150200	-0.55068400
C	-0.17321500	3.06085000	-1.47535000
C	0.57427600	4.19650400	-1.17869000
H	-0.89703200	5.08703200	1.77888400
H	-2.20887900	2.99491500	1.27432500
H	-0.01931400	2.51363200	-2.39880100
H	1.34134500	4.55153800	-1.85678900
N	1.19624100	5.99134600	0.38931400
O	2.36989700	5.92333900	-0.00406500
O	0.74554500	6.88173900	1.07137400
O	-1.81863900	1.43284900	-0.79634800
P	-1.12795500	0.01049600	-0.62801000
O	-0.45968800	-0.17245300	0.75647000
O	0.02232500	-0.26648400	-1.61914000
O	-2.29537200	-1.00956900	-0.91092400
C	-3.65934200	-0.91543100	-0.52141900
C	-4.60750100	-0.95726600	-1.53712800
C	-3.99426200	-0.84952700	0.82841800
C	-5.95650500	-0.92604800	-1.18596500
H	-4.29734700	-1.01502100	-2.57464800
C	-5.34378300	-0.81243600	1.17510300
H	-3.22488700	-0.83261000	1.59381700
C	-6.29829500	-0.85281600	0.16214700
H	-6.73656500	-0.95610300	-1.93730600
H	-5.66289600	-0.75922600	2.20902800
N	-7.73590400	-0.81785300	0.53734400
O	-7.99422800	-0.74697500	1.72773700
O	-8.54630500	-0.86244900	-0.37174400
O	3.65870200	3.42790800	-0.18813800
H	3.17787200	4.28150100	-0.11895800
H	4.60010100	3.62225400	-0.28229000
Ce	1.73571600	-0.66628000	-0.04071900

T1$^{WA}_{Ce}$
Charge = 2, Multiplicity = 1
Energy = -2677.8272911 hartree

O	-1.51592400	-2.96391000	-0.69871900
C	-3.05982300	1.35687100	2.27483400
H	-3.31473800	2.34508900	2.67890100
H	-2.25204800	0.94939500	2.89096900
C	-2.89472500	2.20520100	-1.45293800
H	-3.40588200	2.94400500	-2.08275800
H	-1.81810700	2.32344500	-1.60749600
C	-3.24016200	2.44628800	0.01647300
H	-2.96893900	3.47402000	0.28994500
H	-4.31714300	2.35123600	0.18557700
C	-5.04160100	-0.88143800	-2.22580800
H	-6.10412000	-1.00072400	-2.47398800
H	-4.47104700	-1.37053800	-3.02401200
C	-4.28718300	0.44860400	2.33480700
H	-4.67828300	0.41160000	3.35883700
H	-5.08754400	0.84634100	1.70414500
C	-5.72162500	-1.38231400	0.13564300
H	-6.62065300	-1.97834200	-0.06422200
H	-6.03301700	-0.33455000	0.12296100
C	-4.69425900	0.60547200	-2.16520300
H	-4.97619700	1.08590100	-3.11028300
H	-5.26951100	1.10131700	-1.37857900
C	-5.15631500	-1.75564400	1.50621700
H	-5.93977900	-1.64730000	2.26722600
H	-4.84595100	-2.80703000	1.51571900
N	-4.70889300	-1.57833200	-0.94514200
H	-4.67503700	-2.58069100	-1.14663600
H	-1.68718200	-3.82022200	-1.11448200
N	-3.96037200	-0.92928100	1.85120400
H	-3.45797300	-1.40466800	2.60643400
N	-2.53902000	1.46701700	0.88528900
H	-1.53718800	1.69987500	0.92958500
N	-3.24573600	0.81260300	-1.87834400
H	-2.72349400	0.62422400	-2.73876100
C	5.32326600	-2.95290400	-0.02614500
C	4.64854300	-2.94177600	1.19156600
C	3.56127700	-2.08105900	1.34822400
C	3.19086200	-1.26977500	0.27463300
C	3.87351900	-1.27276800	-0.94034500
C	4.96109000	-2.13020500	-1.09129300
H	4.98117000	-3.58744100	1.99564100

H	3.03456000	-2.01417100	2.29545600
H	3.56766600	-0.61154600	-1.74374800
H	5.52706100	-2.16933600	-2.01444700
N	6.47382100	-3.87969600	-0.19659100
O	7.04461400	-3.85823400	-1.27339800
O	6.75211000	-4.59343300	0.75195400
O	2.14031200	-0.35700600	0.46261000
P	0.55310400	-0.44197300	0.24127900
O	-0.58785600	-0.88372500	1.28155500
O	-0.36581500	-0.23951600	-1.06674300
O	0.43526000	1.19065400	0.73192800
C	1.33546800	2.20760700	0.45610800
C	1.71762700	2.50113300	-0.86008200
C	1.78472300	2.98119000	1.53150000
C	2.55933500	3.58259600	-1.10091600
H	1.35567900	1.89251800	-1.68334000
C	2.62262700	4.06638700	1.29156500
H	1.48378600	2.72120600	2.54097700
C	2.99552400	4.34986900	-0.02126200
H	2.87740700	3.84412000	-2.10320400
H	2.99357300	4.68661900	2.09894400
N	3.88475600	5.50810200	-0.28163500
O	4.22767200	6.17181100	0.68231600
O	4.20360400	5.71118800	-1.44242100
Ce	-2.27251800	-0.86148300	-0.14328700
O	0.74494200	-2.20030800	-0.30477800
H	-0.37539500	-2.76148200	-0.55063500
H	1.41553900	-2.74031100	0.14335400

I_{Ce}^{WA}
Charge = 2, Multiplicity = 1
Energy = -2677.8320478 hartree

O	-1.14559200	-3.14178200	-1.20182400
C	-3.39234000	-0.75497500	2.81335200
H	-3.85040300	-0.24146000	3.66858600
H	-2.57765200	-1.37356100	3.20558800
C	-3.07131100	2.18614900	0.37503300
H	-3.68169500	3.08024600	0.19644500
H	-2.04856600	2.52205800	0.57172600
C	-3.61610900	1.42279800	1.58182200
H	-3.64723800	2.08742200	2.45452000
H	-4.64419700	1.09588100	1.39890700
C	-4.37363000	0.00953900	-2.49625400
H	-5.34254500	-0.02285200	-3.01130100
H	-3.60407600	0.11782300	-3.26950300
C	-4.43844200	-1.62910400	2.12138400
H	-4.88628000	-2.31768500	2.84812400
H	-5.25348900	-1.01449100	1.72921900
C	-5.26464000	-1.79591300	-0.99565100
H	-6.03935500	-2.18977300	-1.66548700
H	-5.71450800	-0.95695800	-0.45810900
C	-4.34106700	1.20803100	-1.54823900
H	-4.55668900	2.12234700	-2.11454900
H	-5.12487300	1.12025900	-0.79074900
C	-4.83837500	-2.88814400	-0.01304600
H	-5.72665800	-3.28826400	0.49248600
H	-4.37728700	-3.72275200	-0.55301000
N	-4.10483300	-1.27636000	-1.78044100
H	-3.88134600	-1.97525700	-2.49349800
H	-1.34478000	-3.96986700	-1.66033100
N	-3.84000600	-2.38736600	0.97959200
H	-3.35743700	-3.20040400	1.37123100
N	-2.78815600	0.21884300	1.86160400
H	-1.88446500	0.52249300	2.23601400
N	-3.03036200	1.31927500	-0.84471900
H	-2.35815200	1.73891400	-1.49257900
C	6.00189100	-2.04359900	0.03914200
C	5.21231700	-2.36973200	1.13824800
C	3.96002600	-1.76765400	1.26709600
C	3.54542500	-0.86718700	0.28400700
C	4.34110500	-0.53463600	-0.81130000
C	5.59294900	-1.13416400	-0.93429400
H	5.58289700	-3.07049400	1.87692600
H	3.33233800	-1.96699400	2.13044700
H	3.99309900	0.18643700	-1.54300700
H	6.25039200	-0.90761300	-1.76525300
N	7.33344200	-2.69146300	-0.10107900
O	8.00128200	-2.37543000	-1.07042700
O	7.65062800	-3.49210500	0.76206900
O	2.31443300	-0.20655900	0.44998600
P	0.82751700	-0.69630400	0.06654700
O	-0.25630000	-1.37475200	1.04710100
O	-0.12677300	-0.39371800	-1.21510500
O	0.27405300	0.87220700	0.76899800
C	0.74007900	2.11871500	0.47857300
C	1.22817500	2.48432100	-0.79192500
C	0.66585700	3.07660900	1.50881000
C	1.63206100	3.79404800	-1.02379800
H	1.28469200	1.75249000	-1.59018000
C	1.06707900	4.38656100	1.27596700
H	0.32684900	2.76741300	2.49265500
C	1.54120600	4.72790300	0.00939000
H	2.01278200	4.10637900	-1.98925400
H	1.03116800	5.13968100	2.05440500
N	1.95593600	6.12680400	-0.25033900
O	1.82116100	6.92291300	0.66424200
O	2.39330100	6.37704000	-1.36197300
Ce	-1.96569800	-0.98739600	-0.24487100
O	1.30582300	-2.16112200	-0.63941400
H	-0.15564300	-3.04003700	-1.12591900
H	2.14903000	-2.54691700	-0.35166800

$T2_{Ce}^{WA}$
Charge = 2, Multiplicity = 1
Energy = -2677.8302368 hartree

O	-1.32286200	-3.21284100	-1.09693200
C	-3.14227600	0.33052400	2.70049900
H	-3.47263700	1.13400800	3.37155700
H	-2.32502400	-0.19752100	3.20338900
C	-2.93613800	2.28373000	-0.59027800
H	-3.49822000	3.13857300	-0.98711500
H	-1.87305300	2.53797500	-0.63988200

C	-3.34677200	2.02575100	0.86068400
H	-3.18070900	2.93649800	1.45013600
H	-4.41648100	1.80377000	0.92486200
C	-4.78337700	-0.55785500	-2.38724300
H	-5.82134400	-0.65745600	-2.73045600
H	-4.14111800	-0.75347500	-3.25414500
C	-4.30411700	-0.62475400	2.43419300
H	-4.71357100	-0.99352500	3.38255100
H	-5.11719700	-0.10177600	1.92258100
C	-5.52235700	-1.75342900	-0.31270700
H	-6.38361600	-2.29343300	-0.72523900
H	-5.88330700	-0.76032300	-0.03276100
C	-4.55343700	0.86334200	-1.87298800
H	-4.83284900	1.58121100	-2.65400900
H	-5.19962500	1.06971500	-1.01563500
C	-4.99912200	-2.49490100	0.91802900
H	-5.82460400	-2.65360400	1.62397100
H	-4.63030400	-3.48713900	0.63291200
N	-4.45944200	-1.58608200	-1.34979900
H	-4.37469800	-2.48209400	-1.83535400
H	-1.56203500	-4.00993900	-1.59054700
N	-3.87079800	-1.76456200	1.56954900
H	-3.38053300	-2.43103900	2.17217000
N	-2.59567400	0.87824600	1.43067100
H	-1.61593700	1.15220800	1.57849000
N	-3.13952200	1.07129800	-1.44597400
H	-2.57583700	1.20273000	-2.29032900
C	5.87427100	-2.26893400	0.05635800
C	5.10937500	-2.48469400	1.19926400
C	3.86952600	-1.85267700	1.30657600
C	3.44401100	-1.03522300	0.25892600
C	4.21323900	-0.81299700	-0.88137100
C	5.45280200	-1.44169800	-0.98268700
H	5.48902400	-3.12488900	1.98672900
H	3.26173300	-1.96463900	2.19934700
H	3.85584500	-0.15532600	-1.66624300
H	6.09068600	-1.29996900	-1.84700600
N	7.19255900	-2.94822800	-0.06023600
O	7.83822000	-2.72903200	-1.07052000
O	7.52169900	-3.67491700	0.86165900
O	2.22211800	-0.34243000	0.40030700
P	0.74889300	-0.85163500	0.03411000
O	-0.32409200	-1.32501900	1.09283500
O	-0.14500800	-0.47380100	-1.21905300
O	0.26168300	0.98696000	0.73667600
C	0.84434200	2.14342600	0.47036000
C	1.48684900	2.42024100	-0.77085100
C	0.78663200	3.16625900	1.46096300
C	2.04339400	3.66548900	-1.00420300
H	1.51687400	1.65601700	-1.53903300
C	1.34881500	4.40873200	1.22675100
H	0.33285600	2.93484000	2.41986300
C	1.96882900	4.64456400	-0.00540200
H	2.53195400	3.90473400	-1.94163000
H	1.33056100	5.19507000	1.97235200
N	2.55998700	5.97727400	-0.26607800
O	2.41754000	6.82373800	0.60115600
O	3.13842300	6.12639000	-1.33051600
Ce	-2.09649200	-0.99778300	-0.24551500
O	1.16850000	-2.35704400	-0.57457600
H	-0.33089100	-3.16862600	-1.01325400
H	1.96076200	-2.78768200	-0.21446700

P_{Ce}^{WA}
Charge = 2, Multiplicity = 1
Energy = -2677.928235 hartree

C	2.82121400	-1.71650000	-2.80643700
H	3.62510000	-1.79393000	-3.54991300
H	1.94171500	-1.30633700	-3.31271000
C	4.80040100	-0.24218200	0.11260700
H	5.84939700	-0.34985700	0.41817700
H	4.58943700	0.82853800	0.03464000
C	4.59326100	-0.91713700	-1.24029800
H	5.29833500	-0.49965600	-1.97010700
H	4.81465800	-1.98535300	-1.16961600
C	3.18391800	-2.77238300	2.47860500
H	3.53997500	-3.70369800	2.93805800
H	2.82713200	-2.12862600	3.28966000
C	2.50430000	-3.09957200	-2.24259000
H	2.27077800	-3.78773400	-3.06527300
H	3.37455900	-3.51432000	-1.72620800
C	2.19485700	-4.24610100	0.72929800
H	2.05880800	-5.15411700	1.33028900
H	3.22308600	-4.26909600	0.35754800
C	4.33064200	-2.09342300	1.73310800
H	5.18024300	-1.94409700	2.41145900
H	4.68739700	-2.73117100	0.91980300
C	1.20762300	-4.24367000	-0.43259900
H	1.33367700	-5.15904200	-1.02577700
H	0.17955000	-4.23649100	-0.05688800
N	2.03193600	-3.02650100	1.56888900
H	1.20182400	-3.16142400	2.14982400
N	1.37574200	-3.02881600	-1.27637700
H	0.51863600	-2.91396700	-1.82028300
N	3.18910200	-0.76616200	-1.71678600
H	3.08944600	0.18165500	-2.08661000
N	3.88192900	-0.80097900	1.14735000
H	3.84035500	-0.11892500	1.90735800
C	-6.59049400	-0.24213700	0.25364500
C	-6.18057800	0.70922200	-0.67760100
C	-4.82975900	0.78498900	-1.01097500
C	-3.94207300	-0.09269100	-0.39559900
C	-4.34478500	-1.04038400	0.54338600
C	-5.69906600	-1.11698200	0.86752800
H	-6.91119400	1.37088900	-1.12753300
H	-4.46677900	1.51258500	-1.72863900
H	-3.63031300	-1.70380200	1.01991300
H	-6.06744600	-1.83740300	1.58796100
N	-8.03101000	-0.32654000	0.60818700
O	-8.34935400	-1.17301100	1.42713400
O	-8.78309700	0.45614000	0.05428300
O	-2.58003400	0.06147900	-0.75973000

P	-1.42905700	-1.01848000	-0.82081400
O	-0.20043400	-0.38025300	-1.49095000
O	-0.92087300	-1.52367200	0.54111300
O	1.74750700	1.25497200	0.29741600
C	1.59717500	2.58779200	0.21719700
C	2.26242800	3.42566600	1.13819400
C	0.77946600	3.13762400	-0.79529600
C	2.11648200	4.80262100	1.04429600
H	2.88563400	2.98745700	1.91185100
C	0.63702400	4.51487200	-0.88676900
H	0.27100600	2.47392600	-1.48773200
C	1.30682400	5.32498500	0.03303500
H	2.61304800	5.47755500	1.73159400
H	0.02069400	4.97457400	-1.65044600
N	1.15070900	6.79959900	-0.06680500
O	0.42906600	7.21702300	-0.95642700
O	1.75603100	7.47340900	0.74906900
O	-1.95692700	-2.29315200	-1.62364900
H	-2.57402300	-2.17195500	-2.36396400
Ce	1.39562900	-0.81351700	0.21123100
O	0.49225600	-0.47785400	2.58171900
H	0.59301300	0.20709400	3.25951200
H	-0.44351900	-0.74462000	2.54745200

R_{Zr}
Charge = 2, Multiplicity = 1
Energy = -2249.9020883 hartree

O	2.77854600	0.23896500	-1.91024800
C	0.69413700	-3.48952700	-1.30281900
H	0.65862400	-4.35279700	-1.97777600
H	-0.31582100	-3.07369500	-1.24493400
C	3.98206300	-3.00930800	-1.53123100
H	3.83641600	-3.85405100	-0.85512900
H	4.91662300	-3.19330600	-2.07500100
C	2.82999000	-2.87591400	-2.52419900
H	3.08159500	-2.12523500	-3.27724500
H	2.66800100	-3.82805400	-3.04261900
C	4.72207200	-0.76782500	1.44080900
H	5.28464200	-0.91835000	2.36960800
H	5.03508500	0.19576800	1.02342100
C	1.16193800	-3.91718400	0.08440100
H	0.41771000	-4.57718800	0.54484900
H	2.09423400	-4.48288700	0.03054500
C	2.70830500	-1.63042200	2.72618600
H	1.84141900	-1.14659100	3.18258700
H	3.44618800	-1.81385800	3.51600400
C	5.01689800	-1.88808200	0.44783900
H	6.06105100	-1.83261500	0.11819900
H	4.88162300	-2.86952400	0.90656500
C	2.28336400	-2.95248700	2.09338600
H	3.14396800	-3.52592400	1.74437200
H	1.77641700	-3.56828800	2.84640500
N	3.25480500	-0.66238200	1.71887000
H	3.10168000	0.26958300	2.10520500
H	2.54452600	0.79761000	-2.66127500
N	1.39665600	-2.70536200	0.92033200
H	0.49211600	-2.41736100	1.30188900
N	1.56871600	-2.41393700	-1.86180600
H	1.01512100	-1.94079700	-2.57759000
N	4.08230900	-1.77432200	-0.70467800
H	4.42494100	-1.03387300	-1.32481100
C	0.05793000	5.15254400	-0.11483700
C	0.14110300	4.54232700	1.13596600
C	-0.39940800	3.26383400	1.29772300
C	-0.99956400	2.64701500	0.19483100
C	-1.10470900	3.26689100	-1.04706500
C	-0.56745400	4.54651900	-1.20203800
H	0.59971200	5.07394300	1.96211500
H	-0.41085200	2.77900200	2.26941500
H	-1.61329100	2.76920400	-1.86585800
H	-0.63255700	5.07624300	-2.14563300
N	0.65913900	6.50313500	-0.29127800
O	0.53360000	7.02273900	-1.38536200
O	1.24354900	6.97268100	0.67042600
O	-1.57376800	1.37283900	0.36280000
P	-0.76810300	0.00932200	0.37060800
O	0.44035600	-0.03268000	1.32077900
O	-0.10927400	-0.33781500	-0.99426100
O	-1.83329700	-1.05998900	0.81804700
C	-3.22642600	-1.11460100	0.51814300
C	-3.65159200	-1.16042100	-0.80642300
C	-4.09683000	-1.17632100	1.59968100
C	-5.01927400	-1.25977400	-1.05813800
H	-2.94083500	-1.12250400	-1.62616700
C	-5.46345100	-1.28192600	1.34294500
H	-3.71653100	-1.14385500	2.61479600
C	-5.89766600	-1.31929600	0.02042700
H	-5.40859400	-1.29577900	-2.06856900
H	-6.18736000	-1.33266800	2.14757300
N	-7.35536700	-1.42847200	-0.25307900
O	-8.09567000	-1.48254600	0.71321400
O	-7.69577700	-1.45507500	-1.42392900
Zr	1.96967100	-0.63009900	-0.30815400
O	2.30773400	1.64574200	0.30334000
H	1.66177400	2.18381500	0.80042800
H	2.73672100	2.20240200	-0.36479700

$T1^{DA}_{Zr}$
Charge = 2, Multiplicity = 1
Energy = -2249.8123031 hartree

O	0.35514900	1.53470200	-0.07346900
C	4.30932200	-1.09178500	2.53158500
H	5.23919700	-1.50957000	2.93480800
H	3.49524700	-1.41785800	3.18832300
C	4.83272400	-2.03449000	-1.15282400
H	5.72390000	-2.09312900	-1.78831200
H	4.32109000	-2.99810100	-1.23836400
C	5.24015800	-1.77810100	0.29765200
H	5.87767600	-2.59186200	0.66181100
H	5.82242400	-0.85517100	0.37553500
C	3.66837700	1.42666000	-2.22161300
H	4.19122600	2.32402200	-2.57166600

H	2.84495300	1.22673700	-2.91341100
C	4.38439200	0.43047100	2.48881200
H	4.53847400	0.83283000	3.49613300
H	5.22992300	0.76165900	1.87903700
C	3.87508300	2.49562000	0.04797400
H	3.89889600	3.53561400	-0.29807400
H	4.90774700	2.13165900	0.03919600
C	4.62186100	0.24004400	-2.16544700
H	5.03667200	0.02752000	-3.15711400
H	5.46731000	0.45089100	-1.50304000
C	3.27934700	2.42740800	1.45269600
H	3.90071400	2.98223400	2.16431100
H	2.28187200	2.87308100	1.45973400
N	3.06480800	1.65082100	-0.87487500
H	2.14389500	2.09165900	-0.99482000
H	-0.14086000	2.03983500	0.59281500
N	3.13237200	0.99512500	1.88494500
H	2.40442300	0.96575700	2.60489700
N	4.02586600	-1.63433800	1.16263000
H	3.61621600	-2.56538300	1.27683600
N	3.90241800	-0.96361700	-1.62945000
H	3.33706800	-1.33700500	-2.39594900
C	-3.56219500	4.09065700	0.04466300
C	-3.37611300	3.25827100	1.14521800
C	-2.76986300	2.01663400	0.95179900
C	-2.36823800	1.66163000	-0.33638000
C	-2.57203400	2.48614200	-1.44058700
C	-3.18202200	3.72361900	-1.24501800
H	-3.71372000	3.57899200	2.12359500
H	-2.65058800	1.32001700	1.77642100
H	-2.27134600	2.15950200	-2.43034900
H	-3.36706000	4.40046500	-2.07068900
N	-4.18797600	5.42260700	0.25515200
O	-4.34764000	6.12029300	-0.73162300
O	-4.48327200	5.71865500	1.40085200
O	-1.81325100	0.38810800	-0.53790700
P	-0.30466600	-0.12548900	-0.41005200
O	0.46604000	-0.59777000	0.97071300
O	0.90800200	-0.21362000	-1.46629100
O	-0.78956100	-1.71311000	-0.77729400
C	-2.02267900	-2.30062800	-0.45379600
C	-2.88535600	-2.62167500	-1.50099000
C	-2.32516400	-2.61338700	0.87336900
C	-4.08474500	-3.27119700	-1.21653300
H	-2.61558500	-2.36657300	-2.52033000
C	-3.52709200	-3.25759400	1.15955900
H	-1.63015900	-2.36169100	1.66850400
C	-4.38522900	-3.57481000	0.10938100
H	-4.78330600	-3.54077000	-1.99960500
H	-3.80403900	-3.52044200	2.17345600
N	-5.66423000	-4.26229800	0.41586100
O	-5.89424100	-4.50843700	1.58928700
O	-6.39107100	-4.52941100	-0.52644000
Zr	2.29749700	-0.35406100	0.09195500
O	1.71503200	-2.62450400	-0.27987900
H	2.02902000	-3.27636900	-0.92421400
H	0.72134100	-2.56203500	-0.39745900

$\mathbf{I_{Zr}^{DA}}$

Charge = 2, Multiplicity = 1

Energy = -2249.8295062 hartree

O	-0.59052900	0.74166600	-2.00705500
C	4.99447700	-1.51222600	-1.80153700
H	5.73743500	-1.30952800	-2.58208600
H	4.67861800	-2.55427500	-1.92498400
C	4.30651900	1.73207300	-1.45161100
H	5.30226300	1.55586700	-1.03787300
H	4.31052100	2.74431200	-1.87088200
C	3.97320900	0.71719900	-2.54352100
H	3.03195800	0.98706700	-3.03256900
H	4.75680100	0.71785700	-3.31018400
C	2.96309800	1.74321500	2.14138200
H	3.40018400	2.10773500	3.07866000
H	1.95502400	2.15703300	2.05707200
C	5.62502800	-1.31509800	-0.42398800
H	6.43831300	-2.03535500	-0.27892300
H	6.06512700	-0.31924900	-0.33847100
C	3.86045200	-0.53645200	2.87955700
H	3.39336000	-1.46494100	3.22771300
H	4.21965100	-0.00956100	3.77217100
C	3.81410200	2.21362800	0.96167200
H	3.80088400	3.30864200	0.91649600
H	4.85831100	1.91555900	1.08054900
C	5.04507300	-0.85559800	1.96622100
H	5.60837400	0.05148900	1.73785400
H	5.73606500	-1.53200200	2.48219800
N	2.79496100	0.25061800	2.18364500
H	1.91710900	0.09170900	2.68101400
H	-1.40265400	1.28185100	-2.03352700
N	4.59315200	-1.44459300	0.66031200
H	4.45119000	-2.44751300	0.81211300
N	3.77660000	-0.66249600	-1.98715400
H	3.15681300	-1.14162800	-2.64462300
N	3.32135700	1.62442200	-0.32595400
H	2.48012000	2.14505700	-0.59542000
C	-3.43944400	4.17154500	0.08456200
C	-2.40440000	3.88797700	0.97480900
C	-1.84155800	2.61506700	0.97232900
C	-2.32716700	1.65228300	0.08048100
C	-3.38400300	1.94021300	-0.79192300
C	-3.94587300	3.21812900	-0.79301500
H	-2.06436600	4.66038000	1.65439200
H	-1.04931600	2.35122900	1.66385000
H	-3.78871600	1.15738100	-1.42754100
H	-4.76799500	3.47535400	-1.45053200
N	-4.02132000	5.53667900	0.07886600
O	-4.93233500	5.74868900	-0.70274300
O	-3.53734800	6.34756300	0.85311500
O	-1.80265900	0.37100900	0.07728600
P	-0.38054600	-0.10883700	-0.65305100
O	0.61661900	0.44070400	0.49747800
O	1.06310500	-0.77161000	-1.42323300

O	-0.80672400	-1.70366500	-0.42958200
C	-2.09913400	-2.23734600	-0.21062900
C	-2.82677000	-2.65720200	-1.32128100
C	-2.55507800	-2.41917800	1.09309800
C	-4.06143200	-3.27437000	-1.12126300
H	-2.42885000	-2.51611600	-2.32114200
C	-3.78490100	-3.04175700	1.29343500
H	-1.97401000	-2.05910900	1.93584300
C	-4.51567700	-3.45657100	0.18211000
H	-4.66425100	-3.61899300	-1.95300200
H	-4.18549200	-3.20297400	2.28703400
N	-5.82779600	-4.11898700	0.39825300
O	-6.18247800	-4.27930400	1.55455200
O	-6.45026200	-4.45202900	-0.59585800
O	1.53586000	-2.55194200	0.59823400
H	0.62596500	-2.57112000	0.18012600
H	1.60445000	-3.24383800	1.27243400
Zr	2.40903800	-0.54977800	-0.04251600

$T2^{DA}_{Zr}$
Charge = 2, Multiplicity = 1
Energy = -2249.8215667 hartree

O	-0.56058400	1.11497000	-1.98682500
C	5.01618700	-1.37829100	-1.75864700
H	5.76816600	-1.19885500	-2.53615200
H	4.67239200	-2.41184900	-1.87768200
C	4.41885000	1.88128300	-1.39412900
H	5.40234800	1.67208500	-0.96696300
H	4.46257000	2.89337900	-1.81201300
C	4.06746600	0.88212500	-2.49599400
H	3.14106800	1.18380300	-2.99427600
H	4.86021800	0.86338800	-3.25295800
C	3.00159900	1.92303500	2.16465400
H	3.42060800	2.26923900	3.11684900
H	2.00369400	2.35689100	2.05961500
C	5.64147600	-1.19031100	-0.37722900
H	6.42675000	-1.93813200	-0.21744500
H	6.11521200	-0.20989100	-0.29425100
C	3.84377400	-0.37705700	2.89895100
H	3.35398100	-1.30081600	3.22685600
H	4.19105200	0.13804800	3.80286000
C	3.88975200	2.38554300	1.00928000
H	3.89339200	3.48083800	0.96707100
H	4.92632000	2.07199700	1.15279800
C	5.03967800	-0.70745400	2.00514900
H	5.62410200	0.19018700	1.79327500
H	5.70823500	-1.40305000	2.52531500
N	2.80588800	0.43361100	2.18651200
H	1.91526200	0.28198300	2.66366500
H	-1.41093300	1.59386900	-1.98340600
N	4.59200000	-1.27435900	0.69069400
H	4.39767500	-2.26897100	0.83931900
N	3.82217500	-0.49726700	-1.95532500
H	3.20374800	-0.95345700	-2.63039400
N	3.41532100	1.80189200	-0.28476500
H	2.59029000	2.33742000	-0.57458600
C	-4.01995200	3.81990700	0.09876000
C	-2.95168300	3.67721700	0.98311600
C	-2.18818500	2.51426200	0.93572200
C	-2.51391800	1.52235900	0.00482700
C	-3.59854600	1.66559200	-0.86734000
C	-4.36218100	2.83353600	-0.82086300
H	-2.73920900	4.46898600	1.69161100
H	-1.35919000	2.35961000	1.61743900
H	-3.86056800	0.85769600	-1.54418300
H	-5.21342300	2.98104500	-1.47505300
N	-4.82094900	5.07008600	0.14316000
O	-5.75111200	5.16003900	-0.63917400
O	-4.48154600	5.91470000	0.95620300
O	-1.78728400	0.33719200	-0.03425600
P	-0.29110500	0.14521100	-0.72794600
O	0.61972400	0.65061800	0.49191100
O	1.10458400	-0.41437400	-1.51247800
O	-0.59266800	-1.60922000	-0.50228300
C	-1.81456800	-2.29085700	-0.24815800
C	-2.52513100	-2.76512800	-1.34609600
C	-2.21075900	-2.52311100	1.06609000
C	-3.68870900	-3.50025300	-1.11985000
H	-2.17015500	-2.57396700	-2.35360200
C	-3.36747600	-3.26681200	1.29100100
H	-1.64668800	-2.11128600	1.89657000
C	-4.08430800	-3.73864600	0.19363000
H	-4.27954300	-3.89351700	-1.93853500
H	-3.72329300	-3.47619400	2.29264200
N	-5.32128200	-4.52807900	0.43703800
O	-5.62169100	-4.73760500	1.60025500
O	-5.93823200	-4.90411100	-0.54444300
O	1.54617300	-2.25059400	0.44665600
H	0.41329600	-2.10952900	-0.06273000
H	1.62006200	-3.02454000	1.01980100
Zr	2.43369900	-0.36600800	-0.03238000

P^{DA}_{Zr}
Charge = 2, Multiplicity = 1
Energy = -2249.9051397 hartree

O	-0.74597300	-2.70543700	-2.01498000
C	3.67727500	-2.11612800	2.54295600
H	4.11334300	-3.00103000	3.02105300
H	3.21374300	-1.51369900	3.33133600
C	3.60748600	-3.51216800	-0.47947000
H	4.63667200	-3.22723400	-0.25254100
H	3.65644400	-4.43903000	-1.06313700
C	2.80751500	-3.76194600	0.79905600
H	1.81553800	-4.14635900	0.54774100
H	3.31274300	-4.51490500	1.41501700
C	3.37984100	-0.40118900	-2.67977100
H	4.09943500	0.08933300	-3.34543800
H	2.43737000	-0.50164800	-3.22415700
C	4.76433300	-1.30444600	1.84077900
H	5.45590800	-0.88913700	2.58345200
H	5.35744700	-1.92909100	1.16963600
C	4.24698200	1.25290200	-0.94390500

H	3.82894200	2.13224700	-0.44284900
H	4.89366300	1.61319300	-1.75250300
C	3.89847600	-1.78233500	-2.27819700
H	3.98912900	-2.40898100	-3.17333600
H	4.89555000	-1.71869500	-1.83750800
C	5.05633100	0.42252200	0.04998600
H	5.62058700	-0.36505500	-0.45405500
H	5.78764000	1.06152700	0.55905200
N	3.08784800	0.47968500	-1.49565400
H	2.40180400	1.16267400	-1.82580200
H	-1.44388100	-2.66896400	-2.68978800
N	4.13502300	-0.22453800	1.02777700
H	3.82182300	0.50312400	1.67662200
N	2.58123000	-2.50761400	1.59913800
H	1.75005100	-2.69253400	2.16554100
N	2.99097900	-2.40445800	-1.26871800
H	2.19646200	-2.80201500	-1.77747900
C	-5.63066800	-2.44173800	0.19690000
C	-5.53398200	-1.48041800	-0.80601100
C	-4.27998000	-0.95324600	-1.11318200
C	-3.17867200	-1.41245500	-0.39863700
C	-3.26838900	-2.36384400	0.61419400
C	-4.52408700	-2.89190400	0.91175300
H	-6.42641800	-1.15678600	-1.32844700
H	-4.16118200	-0.19335600	-1.87819700
H	-2.39001700	-2.67872600	1.16809700
H	-4.65534000	-3.63571000	1.68847900
N	-6.96724400	-3.00697800	0.52224200
O	-7.01022900	-3.84599200	1.40638800
O	-7.91433400	-2.58908700	-0.12036800
O	-1.92655000	-0.81199600	-0.71780500
P	-0.54769900	-1.51617300	-0.98609300
O	0.46549500	-0.45270200	-1.47076700
O	0.17841700	-2.10015900	0.24925400
O	0.03860000	2.66680700	2.51292300
C	-0.40282000	3.69653600	1.69459900
C	-0.48045100	5.01112700	2.15739000
C	-0.75412000	3.36184300	0.38376700
C	-0.90910500	6.01431700	1.29021600
H	-0.20912300	5.25781500	3.18100900
C	-1.17490400	4.36505200	-0.48337200
H	-0.72610000	2.32549800	0.06148200
C	-1.24153500	5.67727900	-0.01922900
H	-0.98326500	7.04545200	1.61481100
H	-1.46335600	4.14855800	-1.50492000
N	-1.67964000	6.74107300	-0.94982800
O	-1.93770000	6.40393600	-2.09648800
O	-1.74609400	7.87599300	-0.50902600
Zr	1.91668600	-0.72629000	0.17824100
O	1.39096500	0.65780800	1.38504500
H	0.07340700	2.94573700	3.43972400
H	0.91373400	1.37987800	1.87183000

R_{Zr}^{WA}
Charge = 2, Multiplicity = 1
Energy = -2326.3711664 hartree

O	-2.64341700	0.69464900	0.05219500
C	0.12652200	-3.83771100	-0.34624600
H	0.49688200	-4.78554800	0.06420300
H	0.97188300	-3.33617600	-0.82788800
C	-1.79135500	-2.58615200	2.69163500
H	-2.35681900	-3.06684500	3.49829500
H	-1.04496400	-1.92889500	3.14408400
C	-1.11161700	-3.63006100	1.81393900
H	-0.43604800	-4.24939000	2.41669000
H	-1.84682700	-4.30460500	1.36408700
C	-4.75251100	-1.69515200	0.47874100
H	-5.71060900	-2.19391400	0.29003600
H	-4.95836100	-0.64525400	0.70192200
C	-0.97946400	-4.09661200	-1.35524400
H	-0.60796400	-4.71033900	-2.18446200
H	-1.79815100	-4.65423800	-0.89138300
C	-3.96348700	-2.98708600	-1.51995300
H	-4.93837200	-3.08227000	-2.01284200
H	-3.86942500	-3.82933500	-0.82819100
C	-4.03377300	-2.35424000	1.64736100
H	-4.63728600	-2.27782200	2.55918800
H	-3.88465200	-3.42026400	1.45333400
C	-2.84145100	-2.99474200	-2.55113100
H	-2.85313500	-3.92976800	-3.12354200
H	-2.97076700	-2.17051800	-3.25698800
N	-3.88416300	-1.72596900	-0.73474400
H	-4.20251900	-0.97028000	-1.34371700
H	-3.11109200	1.52757100	0.34792600
N	-1.53000700	-2.80448300	-1.86231400
H	-0.86975000	-2.45124700	-2.55712000
N	-0.37240800	-2.93663900	0.72505900
H	0.44306800	-2.51161800	1.16782200
N	-2.68956400	-1.73346700	1.85377200
H	-2.82653300	-0.85011200	2.35000100
C	-0.98188900	4.77199900	-0.04911300
C	-0.06072100	4.48439300	-1.05497500
C	0.80619300	3.40672600	-0.87263000
C	0.72999100	2.68651100	0.31781200
C	-0.11708800	3.04306300	1.36509500
C	-1.00193500	4.09806400	1.17095000
H	-0.04638800	5.07449800	-1.96472600
H	1.52597600	3.12838300	-1.63511300
H	-0.09926000	2.49224700	2.29837200
H	-1.70160600	4.38730600	1.94586300
N	-2.02403900	5.77948400	-0.31029700
O	-3.11463600	5.59311100	0.24446800
O	-1.77216300	6.69272900	-1.06275200
O	1.55663200	1.55592200	0.46916900
P	0.98136800	0.07638500	0.44658900
O	0.37053000	-0.32589100	-0.92718700
O	-0.17210300	-0.20822800	1.41646700
O	2.22583000	-0.81178200	0.84087800
C	3.59565000	-0.61731600	0.50611300
C	4.48355500	-0.42871500	1.55887800
C	3.99702300	-0.67967400	-0.82536200
C	5.83933400	-0.28815000	1.26455100

H	4.12408300	-0.39439100	2.58151600
C	5.35260300	-0.53371100	-1.11586700
H	3.27460000	-0.84242500	-1.61902500
C	6.24669100	-0.34105100	-0.06601500
H	6.57463200	-0.13782300	2.04602300
H	5.72258900	-0.57179700	-2.13339900
N	7.69148000	-0.18702000	-0.38057300
O	8.00771300	-0.23410400	-1.55788700
O	8.44801000	-0.02469500	0.56087100
O	-3.97023600	2.87954200	0.69993700
H	-3.67605400	3.79904100	0.53748600
H	-4.89010000	2.92428400	0.99185600
O	-1.80216100	-0.05595200	-2.41605000
H	-2.30910100	0.77188100	-2.37273800
H	-0.89985900	0.17867400	-2.69717500
Zr	-1.64933300	-0.93557100	-0.17394200

T1$_{\mathbf{Zr}}^{\mathbf{WA}}$
Charge = 2, Multiplicity = 1
Energy = -2326.2957924 hartree

O	2.06972900	1.76783600	-1.55275600
C	2.39784900	-2.49030200	1.87539500
H	2.57825600	-3.55828200	2.04700300
H	1.41913600	-2.24928400	2.30318200
C	3.37797700	-2.33829000	-1.80492900
H	4.12562100	-2.85701400	-2.41547200
H	2.40456000	-2.44204000	-2.28751900
C	3.33564400	-2.92395000	-0.39916500
H	3.07289600	-3.98767500	-0.43984200
H	4.31477800	-2.85278700	0.08629600
C	5.35706200	0.87223400	-1.26036700
H	6.42768800	1.08585000	-1.15989200
H	4.94792100	1.56364700	-2.00287200
C	3.48467100	-1.65014700	2.52972700
H	3.49566200	-1.79628100	3.61571800
H	4.47172000	-1.93820100	2.15638600
C	5.38507900	0.63058900	1.23073300
H	6.27403000	1.24569700	1.41346700
H	5.73542200	-0.38291100	1.01677000
C	5.13709700	-0.56721600	-1.70853000
H	5.58774500	-0.72627600	-2.69514300
H	5.62115500	-1.26887000	-1.02278100
C	4.47317700	0.63311200	2.45105800
H	5.01861100	0.27214400	3.33075200
H	4.13220700	1.64712000	2.67522600
N	4.63858200	1.12533000	0.03104300
H	4.56557500	2.13939500	0.13302000
H	2.37965200	2.67528600	-1.66667500
N	3.26562200	-0.20988100	2.19228100
H	2.52191500	0.13787000	2.80199100
N	2.34613000	-2.17023300	0.42413400
H	1.41785600	-2.40866800	0.06414700
N	3.67952100	-0.87333500	-1.73391500
H	3.27131400	-0.44095100	-2.56659000
C	-3.47549500	4.14402300	0.14437100
C	-2.81493000	3.50417600	1.19247300
C	-2.29212700	2.22978300	0.98500400
C	-2.44463100	1.62516200	-0.26856600
C	-3.14254500	2.26050600	-1.30342200
C	-3.66207300	3.53967500	-1.09519100
H	-2.73260500	4.00397100	2.15066600
H	-1.80856700	1.68539500	1.78877900
H	-3.31651000	1.73699000	-2.23975400
H	-4.21359400	4.06017400	-1.86955400
N	-4.00210700	5.51525200	0.36391300
O	-4.59954900	6.03392000	-0.56274700
O	-3.78353600	6.02200000	1.45273200
O	-1.96498600	0.34535900	-0.48846200
P	-0.40670000	-0.15405900	-0.73473300
O	0.32006200	0.38824100	0.59685300
O	1.01351200	-0.62322500	-1.39405200
O	-0.89212800	-1.70934300	-0.68679800
C	-2.14918000	-2.25563700	-0.37517300
C	-2.92089500	-2.75398100	-1.42196500
C	-2.53734700	-2.38120400	0.95795500
C	-4.12605600	-3.38920100	-1.12791800
H	-2.57908800	-2.65199900	-2.44669300
C	-3.74019400	-3.01733500	1.25345600
H	-1.91218500	-1.98494800	1.75212400
C	-4.51395400	-3.50851000	0.20422700
H	-4.75963700	-3.79209400	-1.90898800
H	-4.08477500	-3.13833200	2.27335400
N	-5.79588700	-4.18606700	0.52052000
O	-6.09708100	-4.27967800	1.69977000
O	-6.45304600	-4.59805300	-0.42063600
O	-0.24113800	1.20118100	-1.95695400
H	0.88382800	1.58274200	-1.93849100
H	-0.95218100	1.86760600	-2.00077200
Zr	2.35765200	0.22494100	-0.05701000
O	1.95439600	2.14455900	1.38622100
H	2.19929300	3.08164200	1.39362100
H	0.97903300	2.07354800	1.43369900

I1$_{\mathbf{Zr}}^{\mathbf{WA}}$
Charge = 2, Multiplicity = 1
Energy = -2326.308682 hartree

O	2.31216800	1.20243600	-2.25165300
C	2.05090000	-2.01965500	2.36177400
H	2.12081600	-3.01716100	2.81140900
H	1.08609300	-1.59239100	2.65270300
C	3.14695700	-2.94694600	-1.17085600
H	3.84099700	-3.68578900	-1.58722700
H	2.17888900	-3.06466300	-1.66274500
C	2.99840900	-3.13721100	0.33324800
H	2.61078500	-4.13908300	0.55224300
H	3.96400000	-3.04773800	0.84211000
C	5.45372900	0.08764900	-1.34603300
H	6.53589500	0.21834700	-1.22830600
H	5.16038800	0.57895100	-2.27867200
C	3.19015500	-1.13214800	2.84311800
H	3.14648800	-0.99435300	3.92942300
H	4.15855900	-1.58808600	2.61643400

C	5.34691400	0.55872900	1.10976000
H	6.27995900	1.13002600	1.18056500
H	5.61405000	-0.49789600	1.19994300
C	5.09672200	-1.39347400	-1.40230000
H	5.57692500	-1.86107200	-2.26991400
H	5.46359300	-1.92032000	-0.51660200
C	4.39090700	0.96552700	2.22645400
H	4.87199400	0.82023800	3.20099600
H	4.13743800	2.02604300	2.14664500
N	4.70879100	0.76079100	-0.23205200
H	4.74205200	1.76472600	-0.41921400
H	2.55563600	2.08240100	-2.57524700
N	3.11995000	0.18385200	2.13745300
H	2.38768400	0.73408300	2.59174200
N	2.07959300	-2.09229900	0.87463000
H	1.13956600	-2.32408300	0.54018600
N	3.61669600	-1.55502800	-1.46022700
H	3.30596900	-1.32912900	-2.40841600
C	-3.00714000	4.41185000	0.15736500
C	-2.21078000	3.82828600	1.14231700
C	-1.85000100	2.48917200	1.01549100
C	-2.28918500	1.75605300	-0.09746200
C	-3.12643800	2.34524200	-1.05419000
C	-3.48687900	3.68753500	-0.92979000
H	-1.90355900	4.42270300	1.99504900
H	-1.27274800	1.99074400	1.78604000
H	-3.53369300	1.74055900	-1.86097500
H	-4.13568100	4.16971600	-1.65173100
N	-3.35697900	5.84705300	0.28016100
O	-4.09728100	6.31422800	-0.56800500
O	-2.86380000	6.45828000	1.21637500
O	-1.95819700	0.42704100	-0.24183800
P	-0.47440600	-0.17978800	-0.77443600
O	0.39661800	0.50633000	0.42496800
O	0.98577600	-0.82463600	-1.36576000
O	-1.00563600	-1.70056800	-0.56914300
C	-2.30381600	-2.17123200	-0.29309200
C	-3.07417800	-2.62347600	-1.36171600
C	-2.73321300	-2.27729200	1.02804200
C	-4.32180300	-3.18844000	-1.10226400
H	-2.69793500	-2.54581500	-2.37664700
C	-3.97750100	-2.84469200	1.28933300
H	-2.11227700	-1.91127600	1.83916900
C	-4.75056500	-3.28874000	0.21862400
H	-4.95571200	-3.55384200	-1.90125800
H	-4.35564900	-2.94596900	2.29941600
N	-6.07638400	-3.89351500	0.49844000
O	-6.41113800	-3.97879800	1.66917700
O	-6.73342800	-4.25990200	-0.46172900
O	-0.52451500	0.68951900	-2.18037700
H	1.43678300	0.95149500	-2.63196200
H	-1.33051400	1.22066300	-2.30460400
Zr	2.35047600	0.05491000	-0.19237100
O	2.09897600	2.36595200	0.49189500
H	2.45387300	3.25940000	0.37478100
H	1.12923300	2.41353100	0.61898200

$I2_{Zr}^{WA}$
Charge = 2, Multiplicity = 1
Energy = -2326.3026485 hartree

O	0.61091000	0.72003300	1.79287500
C	-4.70749100	-2.61587100	0.01861800
H	-5.32384100	-3.30268500	0.61177400
H	-4.31384900	-3.18966800	-0.82813900
C	-4.24259700	-0.39326600	2.45994700
H	-5.26451000	-0.24415800	2.10213700
H	-4.24578600	-0.16453400	3.53260400
C	-3.76656700	-1.82768600	2.24663700
H	-2.80652700	-1.97093900	2.74931400
H	-4.48469200	-2.53272500	2.68167500
C	-3.11397800	2.71235600	0.59140200
H	-3.56695600	3.69785100	0.43264200
H	-2.07249900	2.87197500	0.89009100
C	-5.56315200	-1.45683300	-0.48474700
H	-6.34234700	-1.82898700	-1.15963600
H	-6.07331500	-0.96377500	0.34409800
C	-4.30456300	2.00869800	-1.54224500
H	-3.97624300	1.90122400	-2.57986100
H	-4.77552200	2.99525800	-1.45540100
C	-3.85714800	1.94624700	1.68138900
H	-3.71950800	2.44535300	2.64779300
H	-4.93227900	1.92172300	1.48863600
C	-5.33118800	0.92601500	-1.20588100
H	-5.77735700	1.10471500	-0.22637200
H	-6.14884500	0.96168700	-1.93582800
N	-3.07508200	1.93443400	-0.68950100
H	-2.30886900	2.33098800	-1.23547400
H	1.44005100	1.22915300	1.86386400
N	-4.70472400	-0.43399500	-1.16649900
H	-4.59918800	-0.74038100	-2.13717100
N	-3.53103900	-2.13551300	0.80046300
H	-2.83578500	-2.88153800	0.77399700
N	-3.35442000	0.54750200	1.72135100
H	-2.45001600	0.54238400	2.20573700
C	3.66703000	4.11009700	-0.00211700
C	2.64041300	3.91366800	-0.92503600
C	2.03563400	2.66287900	-1.00591100
C	2.46957100	1.63215800	-0.16309800
C	3.51789700	1.83592800	0.74373000
C	4.12288800	3.09095400	0.82816900
H	2.33913400	4.73474700	-1.56468900
H	1.24718900	2.46985300	-1.72464500
H	3.87993600	1.00526800	1.34308400
H	4.93952400	3.28189300	1.51447300
N	4.29417300	5.45116600	0.09258100
O	5.19530600	5.58727600	0.90230300
O	3.85441900	6.32120500	-0.64311000
O	1.90615700	0.37284500	-0.24039000
P	0.42905000	-0.10036200	0.40870600
O	-0.47481900	0.52481200	-0.78310600
O	-1.04168000	-0.72366000	1.07651800
O	0.85361900	-1.69157800	0.15448500

C	2.14994200	-2.24159000	0.05154600
C	2.77509000	-2.66311100	1.22305700
C	2.71714800	-2.43888200	-1.20543500
C	4.01508500	-3.29485200	1.13589000
H	2.29345600	-2.51131200	2.18383000
C	3.95243400	-3.07624200	-1.29455700
H	2.21487500	-2.08169600	-2.09853300
C	4.57949500	-3.49107900	-0.12167200
H	4.53871100	-3.64101900	2.01899800
H	4.43533000	-3.24923200	-2.24879100
N	5.89748200	-4.16861200	-0.21797000
O	6.35420300	-4.33476400	-1.33708000
O	6.42391600	-4.50743900	0.82861900
O	-1.45971900	-2.35992900	-1.09006200
H	-0.58556600	-2.42916300	-0.61600700
H	-1.33833000	-2.65700100	-2.00365600
Zr	-2.37891300	-0.33103300	-0.32943700
O	-2.02779200	-0.02464300	-2.68748800
H	-2.35021400	-0.09886700	-3.59747900
H	-1.09191900	0.26469700	-2.67579500

$T2_{Zr}^{WA}$
Charge = 2, Multiplicity = 1
Energy = -2326.291358 hartree

O	0.43182200	1.37763100	1.71773300
C	-4.61630500	-2.40430000	0.63922100
H	-5.22455000	-2.92860600	1.38652800
H	-4.15778600	-3.16925700	0.00366800
C	-4.31177700	0.44690500	2.37518200
H	-5.31968500	0.45760500	1.95198200
H	-4.36591200	0.96500100	3.34028500
C	-3.80149800	-0.97609000	2.58178500
H	-2.86094700	-0.94987500	3.13752300
H	-4.52444900	-1.55686100	3.16649400
C	-3.20817800	2.94395300	-0.25308300
H	-3.67498300	3.84162200	-0.67547000
H	-2.17012100	3.19389200	-0.00936200
C	-5.50311000	-1.48415500	-0.19475200
H	-6.22943900	-2.07131600	-0.76750100
H	-6.07524700	-0.81448500	0.44943900
C	-4.37968600	1.64889000	-2.10214800
H	-4.04433200	1.29229800	-3.07959300
H	-4.89379700	2.60378300	-2.26450300
C	-3.94511100	2.49353300	1.00287100
H	-3.83193100	3.24313000	1.79472700
H	-5.01696500	2.39022500	0.81654500
C	-5.35583900	0.64747100	-1.48757400
H	-5.80722000	1.05112200	-0.57966400
H	-6.17511900	0.45470500	-2.19034300
N	-3.15146600	1.84503700	-1.26834100
H	-2.40000900	2.09239100	-1.91234700
H	1.27512100	1.86554200	1.75178000
N	-4.66809900	-0.63016300	-1.10763200
H	-4.53580000	-1.16251500	-1.97201400
N	-3.50276600	-1.65111000	1.28201100
H	-2.75107500	-2.31756400	1.45947600
N	-3.41369900	1.17188200	1.43229200
H	-2.52886000	1.32596300	1.92798100
C	4.30035300	3.69805700	0.00159100
C	3.32753300	3.55521300	-0.98660200
C	2.50034700	2.43587500	-0.96029200
C	2.66829000	1.48772500	0.05476400
C	3.65570800	1.63183100	1.03499400
C	4.48359100	2.75539100	1.00861000
H	3.23697200	4.31184400	-1.75687400
H	1.74323500	2.28323400	-1.72181600
H	3.79117300	0.85898900	1.78534700
H	5.26431900	2.90138700	1.74581600
N	5.17148400	4.90133400	-0.02348300
O	6.01532200	4.99207100	0.85122400
O	4.97057700	5.70970200	-0.91530600
O	1.88540600	0.33722400	0.06724300
P	0.31047700	0.23049300	0.57514600
O	-0.47201600	0.66121800	-0.75571500
O	-1.08756200	-0.30187500	1.28283200
O	0.62088800	-1.57779800	0.39760000
C	1.82681900	-2.28286000	0.18140100
C	2.50464800	-2.76316000	1.29870000
C	2.25828900	-2.53756000	-1.11883300
C	3.65793900	-3.52378000	1.11015600
H	2.12964300	-2.55374100	2.29525400
C	3.40522500	-3.30490000	-1.30911400
H	1.72456300	-2.12559400	-1.96950200
C	4.08374800	-3.78221600	-0.19014800
H	4.21913300	-3.92119000	1.94749300
H	3.78251900	-3.53036100	-2.29931500
N	5.30908700	-4.59860300	-0.39488600
O	5.64023600	-4.81744200	-1.54837600
O	5.88822600	-4.98666200	0.60495300
O	-1.50105900	-2.19268700	-0.59589100
H	-0.36698000	-2.04601300	-0.05186600
H	-1.47409000	-2.79571200	-1.34940900
Zr	-2.39139500	-0.22722500	-0.29645500
O	-1.92132600	-0.22458800	-2.68294500
H	-2.03500700	-0.81398800	-3.44329200
H	-0.98555100	0.05801900	-2.63337900

P_{Zr}^{WA}
Charge = 2, Multiplicity = 1
Energy = -2326.33555186 hartree

O	0.29144500	3.04867300	-1.57394900
C	-3.66435100	1.46851200	2.74595900
H	-4.06324000	2.31104000	3.32284900
H	-3.26960500	0.75235300	3.47356800
C	-3.47993200	3.34513800	0.08848500
H	-4.53422800	3.18167100	0.32078800
H	-3.40873100	4.33333300	-0.37995000
C	-2.62013500	3.29976700	1.34418200
H	-1.59901500	3.61293300	1.11401800
H	-3.01868600	3.98168000	2.10435900
C	-3.71129400	0.66034300	-2.61466600
H	-4.53396200	0.35281200	-3.27101400

H	-2.83229200	0.83435300	-3.23988400
C	-4.78360900	0.82218300	1.93149700
H	-5.51692500	0.36305100	2.60533700
H	-5.32014800	1.56693600	1.34082200
C	-4.47580700	-1.30448900	-1.19633800
H	-4.03458900	-2.25393300	-0.87345700
H	-5.17017800	-1.52939900	-2.01448000
C	-4.08214100	1.94252400	-1.87840500
H	-4.19630500	2.76119700	-2.59909500
H	-5.03702000	1.84491500	-1.35871900
C	-5.21576400	-0.64714500	-0.03723600
H	-5.78735400	0.21989300	-0.37327100
H	-5.93245500	-1.35030600	0.40239600
N	-3.34878400	-0.44598600	-1.67177600
H	-2.72063400	-1.06282200	-2.18608400
H	0.92434100	3.35094300	-2.24556700
N	-4.22582500	-0.18603700	0.98250300
H	-3.96536000	-1.00777900	1.53411200
N	-2.52537000	1.90533900	1.88202700
H	-1.68089800	1.89065000	2.45616800
N	-3.04230400	2.27473100	-0.85842000
H	-2.24493500	2.65244600	-1.37707000
C	5.37360900	2.49441200	0.11488100
C	5.08452500	2.55696500	-1.24571300
C	3.86583500	2.04845400	-1.69590400
C	2.99295300	1.50500800	-0.75891500
C	3.28167100	1.41816500	0.59955300
C	4.50120800	1.93024800	1.04245800
H	5.80528400	2.98945400	-1.92950300
H	3.61758900	2.04774600	-2.75273700
H	2.59038300	0.93662400	1.28236000
H	4.78450700	1.89107100	2.08752400
N	6.66979700	3.04377100	0.59668500
O	6.88741600	2.97134300	1.79420500
O	7.41026300	3.52623100	-0.24220600
O	1.76735100	0.95606700	-1.24558400
P	0.32700400	1.54984800	-1.04069900
O	-0.70444100	0.64648100	-1.73878500
O	-0.21649400	1.59455300	0.40928000
O	1.28589500	-1.31834200	1.63796100
C	1.44170400	-2.58438100	1.16524600
C	2.62870500	-2.88622500	0.48150200
C	0.44469200	-3.56834700	1.32346600
C	2.81180800	-4.15381900	-0.05723600
H	3.39436300	-2.12322200	0.38923500
C	0.63196000	-4.84292300	0.77834000
H	-0.42438200	-3.36961200	1.94767800
C	1.80430300	-5.11207700	0.08299800
H	3.72019600	-4.41362800	-0.58845700
H	-0.10820000	-5.62541400	0.89957000
N	1.98778700	-6.44948300	-0.52094200
O	1.06296700	-7.24058300	-0.40938700
O	3.04269800	-6.66036100	-1.09579900
O	-1.55071800	-0.81767100	1.79400400
H	0.37157400	-1.18205100	1.94909200
H	-1.76589900	-0.82029700	2.73423100
Zr	-1.97484700	0.30635300	0.16153100
O	-1.07177600	-1.70372400	-0.62599600
H	-0.71075300	-2.40159100	-0.02938500
H	-0.46720500	-1.60843400	-1.38160200

R_{Ti}
Charge = 2, Multiplicity = 1
Energy = -2261.1270692 hartree

O	2.40597000	1.13383700	-0.18235000
C	2.66726800	-2.78820500	-2.10961200
H	3.31540300	-3.21565000	-2.88272300
H	1.63657100	-2.82964400	-2.46882900
C	5.16311100	-1.19791800	-0.72596100
H	5.29966400	-2.26005800	-0.51386400
H	6.15962500	-0.74110600	-0.74933900
C	4.43713600	-0.97053200	-2.04409400
H	4.47262500	0.08956800	-2.31424000
H	4.90375100	-1.53694300	-2.85761800
C	3.67818300	-0.44113400	2.67807100
H	3.83955500	-0.80781600	3.69779300
H	3.69471200	0.65284500	2.71186800
C	2.78077600	-3.56698600	-0.80507800
H	2.39242400	-4.58175000	-0.94634900
H	3.81680900	-3.65877400	-0.47277400
C	1.84836800	-2.19920800	2.60345900
H	0.75746800	-2.15354400	2.61263200
H	2.18754300	-2.41639500	3.62235100
C	4.75580300	-0.94819200	1.73051100
H	5.72511000	-0.49431300	1.96927900
H	4.88045900	-2.03099400	1.79400400
C	2.31489500	-3.27824200	1.63205100
H	3.39043800	-3.45158100	1.71207600
H	1.81923600	-4.22762600	1.86580100
N	2.32734600	-0.84147200	2.17729300
H	1.64860700	-0.16998500	2.53928700
H	1.99410800	1.98274500	-0.41878300
N	2.00497800	-2.84539100	0.24134300
H	1.01725800	-3.10951800	0.06092000
N	2.99529800	-1.33736100	-1.90250900
H	2.47049200	-0.82005300	-2.60917300
N	4.32914000	-0.60554300	0.35089700
H	4.39085900	0.41159100	0.25694900
C	0.17260800	4.83684900	-0.05528700
C	-0.65871000	4.26758200	0.90557000
C	-1.16200900	2.98332500	0.68844700
C	-0.81507100	2.32439300	-0.48646200
C	-0.00778100	2.90275700	-1.46859700
C	0.49906600	4.18504600	-1.24396500
H	-0.90320800	4.82609900	1.80174100
H	-1.82112700	2.51068500	1.40866500
H	0.17882100	2.38118800	-2.40230300
H	1.11816800	4.68814800	-1.97796600
N	0.73661600	6.19191100	0.19284700
O	1.50573500	6.62754900	-0.64728500
O	0.39697800	6.74999000	1.22034800
O	-1.35151500	1.03772300	-0.71698600

P	-0.60461300	-0.33553500	-0.49068700
O	0.16989700	-0.41016400	0.84838800
O	0.55749700	-0.64295400	-1.46174300
O	-1.75580900	-1.39802600	-0.66953100
C	-3.15131800	-1.23013500	-0.38613100
C	-4.00231800	-0.96352700	-1.45179300
C	-3.59035100	-1.41025900	0.92104700
C	-5.36875900	-0.86110500	-1.19099800
H	-3.61328900	-0.84315300	-2.45709500
C	-4.95753000	-1.30431300	1.17642100
H	-2.89051000	-1.63884700	1.71835600
C	-5.81767000	-1.03088200	0.11628700
H	-6.08098000	-0.65449500	-1.98108100
H	-5.36044700	-1.43461100	2.17372800
N	-7.27596300	-0.91760800	0.39317000
O	-7.62921800	-1.06907300	1.55012800
O	-8.00012600	-0.68064200	-0.55729500
Ti	2.05753500	-0.60747100	-0.02039900
O	-0.45307700	-3.98321900	-0.49647000
H	-1.20739900	-3.44875000	-0.78488800
H	-0.73605400	-4.90767000	-0.47158100

$\mathbf{T1^{DA}_{Ti}}$

Charge = 2, Multiplicity = 1

Energy = -2261.0475896 hartree

O	0.91025500	0.86242900	-1.21011800
C	2.89569700	-0.85039600	2.75236200
H	3.41015300	-1.34105800	3.58592000
H	1.86284100	-0.65994500	3.05310600
C	3.96782100	-3.25384200	0.01173400
H	4.87311900	-3.81560100	-0.23980500
H	3.11287900	-3.93283900	-0.01641200
C	4.06541100	-2.60473100	1.38458100
H	4.11699200	-3.36085700	2.17611000
H	4.95943600	-1.97699500	1.47095100
C	4.55645500	-0.31453600	-2.33684800
H	5.43588000	0.21873400	-2.71604400
H	3.91706300	-0.55376600	-3.19235500
C	3.59783800	0.45300700	2.37765400
H	3.59033800	1.15906900	3.21547300
H	4.64585200	0.27367500	2.11484900
C	4.58921900	1.47221400	-0.56501200
H	5.07631700	2.23385100	-1.18472200
H	5.38042100	0.87595000	-0.09895300
C	4.95037500	-1.58234700	-1.59127600
H	5.43607500	-2.29915100	-2.26093800
H	5.65220700	-1.36504400	-0.78007900
C	3.69323300	2.11643000	0.49031600
H	4.28735300	2.70500000	1.19854800
H	2.97892800	2.79767200	0.01728000
N	3.76083900	0.55811000	-1.41312400
H	3.12478500	1.12809800	-1.97829500
H	0.60729500	1.76608800	-1.00648900
N	2.91577800	1.04827200	1.18357600
H	2.01345300	1.41514100	1.49197500
N	2.85349800	-1.75099200	1.55956200
H	2.03169700	-2.38757500	1.62138900
N	3.71506800	-2.17162500	-0.99346600
H	3.15129200	-2.56561600	-1.75201000
C	-1.79285500	4.59226000	0.07437000
C	-1.60736600	3.76093500	1.17824900
C	-1.47953300	2.38848900	0.97323300
C	-1.54208100	1.89360800	-0.33368000
C	-1.74851000	2.72753500	-1.43564000
C	-1.87526400	4.10116100	-1.22602300
H	-1.57952500	4.19147700	2.17255900
H	-1.36841700	1.70537400	1.80891200
H	-1.83324000	2.30360200	-2.43173000
H	-2.04433300	4.78546600	-2.04894000
N	-1.90084300	6.05936700	0.29619300
O	-2.08009800	6.75398400	-0.68829200
O	-1.78730600	6.45155300	1.44535900
O	-1.47275700	0.51488000	-0.54294500
P	-0.16986000	-0.44707900	-0.49986700
O	0.59673600	-0.38344100	0.87573000
O	1.10613000	-1.35399200	-1.23620300
O	-1.07308100	-1.79945700	-0.59090400
C	-2.46131400	-1.94565200	-0.38295000
C	-3.26494600	-2.16939500	-1.49744200
C	-2.96190500	-1.96191900	0.91789300
C	-4.62447900	-2.40871200	-1.30537500
H	-2.83288600	-2.16482800	-2.49247400
C	-4.32122100	-2.20163200	1.10887700
H	-2.30425300	-1.79644000	1.76578600
C	-5.12688800	-2.41831800	-0.00645700
H	-5.29160100	-2.58929600	-2.13977100
H	-4.76103900	-2.22650000	2.09933400
N	-6.57657400	-2.67164200	0.19981100
O	-6.97954300	-2.67183000	1.35153500
O	-7.25314400	-2.85902100	-0.79668600
Ti	2.34618700	-0.60780900	-0.19883000
O	0.81261400	-3.63311000	1.03997500
H	0.10871400	-3.30147500	0.45641700
H	0.50307800	-4.46557100	1.42380900

$\mathbf{I^{DA}_{Ti}}$

Charge = 2, Multiplicity = 1

Energy = -2261.0728301 hartree

O	-0.42348400	-0.67864700	1.84400600
C	4.98126900	1.31096500	2.01777900
H	5.67713400	0.99824000	2.80470700
H	4.69066400	2.34423500	2.23615600
C	4.25036500	-1.84350500	1.38717700
H	5.28347300	-1.66783800	1.07781100
H	4.17921300	-2.89247200	1.69457100
C	3.84989500	-0.91619800	2.52697500
H	2.86459700	-1.19470600	2.91356700
H	4.56427900	-0.98749700	3.35514500
C	3.15399900	-1.45715100	-2.25712500
H	3.66076400	-1.68072200	-3.20302400
H	2.16527500	-1.91936100	-2.29000000
C	5.64439900	1.23217500	0.65006000
H	6.45077700	1.96995900	0.56476700

H	6.08849800	0.24823800	0.48200600
C	3.94874500	0.90993900	-2.72906400
H	3.45595900	1.86195200	-2.95625400
H	4.30747700	0.50810300	-3.68435800
C	3.95289300	-2.02712800	-1.08937600
H	3.96922000	-3.12188600	-1.14446800
H	4.99195200	-1.69055500	-1.11286200
C	5.11657800	1.13155500	-1.77545700
H	5.72787200	0.22946200	-1.70070300
H	5.77202900	1.92857600	-2.14476300
N	2.92941900	0.01749100	-2.10987100
H	2.02623300	0.20583100	-2.54376700
H	-1.23963400	-1.21498300	1.87625300
N	4.60697400	1.44512000	-0.40435500
H	4.36202200	2.43928700	-0.38129600
N	3.73619100	0.49010400	2.03574600
H	3.05674000	0.95290500	2.63965100
N	3.36636600	-1.57351800	0.20959800
H	2.48692200	-2.08098500	0.34479900
C	-3.35559500	-4.10618400	-0.09418900
C	-2.28682600	-3.93945000	-0.97367100
C	-1.66449000	-2.69628200	-1.05081800
C	-2.12904100	-1.65053000	-0.24803900
C	-3.21745700	-1.81957100	0.61535200
C	-3.83793700	-3.06792800	0.69621100
H	-1.96582900	-4.77589800	-1.58315300
H	-0.84447000	-2.52271100	-1.73901200
H	-3.60209500	-0.97257000	1.17689900
H	-4.68741000	-3.23800300	1.34730700
N	-4.00416600	-5.43972500	-0.00368500
O	-4.93972000	-5.54860700	0.76955300
O	-3.54443600	-6.32562700	-0.70585300
O	-1.54011400	-0.39390700	-0.32943300
P	-0.18050700	0.10567800	0.46196700
O	0.90484300	-0.43273100	-0.58102800
O	1.31359300	0.77230800	1.29035900
O	-0.56843000	1.69326200	0.26496300
C	-1.86725100	2.25099400	0.13015900
C	-2.51189400	2.67473500	1.28927900
C	-2.40289800	2.44152400	-1.14061700
C	-3.74819600	3.31029300	1.17281900
H	-2.04977700	2.52659500	2.26010100
C	-3.63502800	3.08182300	-1.25609400
H	-1.88032100	2.08210900	-2.02053100
C	-4.28419300	3.50289000	-0.09752300
H	-4.28946800	3.66163900	2.04321900
H	-4.09737000	3.25346100	-2.22078500
N	-5.59926900	4.18506000	-0.22408100
O	-6.03005900	4.34828000	-1.35319600
O	-6.14530700	4.52790100	0.81049700
O	1.89740900	2.42865000	-0.58000100
H	0.98372700	2.53948200	-0.20811000
H	1.96985500	2.93357900	-1.40351200
Ti	2.55647000	0.48885400	0.09021400

$\mathbf{T2_{Ti}^{DA}}$

Charge = 2, Multiplicity = 1
Energy = -2261.0604496 hartree

O	-0.39521900	1.08210300	-1.92800500
C	5.04816700	-1.21192500	-1.85924900
H	5.76841400	-0.93654000	-2.63788900
H	4.75472500	-2.25143900	-2.04084000
C	4.36330600	1.96161200	-1.34534300
H	5.38264300	1.77358500	-1.00106200
H	4.32620000	3.00104900	-1.68947400
C	3.97016300	1.01048800	-2.46975500
H	3.00202000	1.29888900	-2.88905400
H	4.70884600	1.04173000	-3.27885600
C	3.10683600	1.70996100	2.25374000
H	3.56952300	1.93207800	3.22209600
H	2.13115700	2.20000900	2.22953300
C	5.66817900	-1.08034000	-0.47499800
H	6.45766100	-1.82745300	-0.33053700
H	6.12339400	-0.09789900	-0.33151000
C	3.81254900	-0.66504100	2.80468200
H	3.28968600	-1.60765100	2.99992000
H	4.11150400	-0.25960600	3.77858100
C	3.97812500	2.23687300	1.11743100
H	4.01031000	3.33238000	1.14374300
H	5.00830800	1.88391500	1.20237700
C	5.03330000	-0.90874500	1.92685000
H	5.66102300	-0.01650900	1.87420800
H	5.65509300	-1.71073700	2.34119500
N	2.84975200	0.23950200	2.11410600
H	1.91981000	0.07691400	2.49996200
H	-1.25726700	1.54182000	-1.93303600
N	4.58736700	-1.23373300	0.53972000
H	4.30880300	-2.21842500	0.52557500
N	3.81089000	-0.38322500	-1.94976200
H	3.15509300	-0.85005400	-2.57726700
N	3.44147000	1.75005900	-0.18685100
H	2.57552300	2.26505500	-0.37243700
C	-3.91286800	3.76901000	0.10425000
C	-2.83099000	3.71122100	0.98142700
C	-2.02179100	2.57813000	0.97925800
C	-2.31877500	1.53283400	0.10016500
C	-3.41516600	1.59029100	-0.76646500
C	-4.22409700	2.72861200	-0.76531500
H	-2.64301400	4.54349700	1.64934400
H	-1.18120700	2.48953400	1.65892500
H	-3.65379100	0.74090100	-1.39996700
H	-5.08724000	2.81171200	-1.41535200
N	-4.76575400	4.98665500	0.10173500
O	-5.70691500	5.00028300	-0.67209500
O	-4.45291300	5.88001800	0.87126300
O	-1.54252600	0.37418900	0.10997100
P	-0.08209100	0.18265200	-0.63456500
O	0.87435100	0.68021100	0.53457000
O	1.34500600	-0.35201900	-1.41971500
O	-0.35881100	-1.56893700	-0.46253700
C	-1.56833300	-2.29769300	-0.23798000
C	-2.19878000	-2.83314300	-1.35536500

C	-2.01803500	-2.49931100	1.06286600
C	-3.34136700	-3.61026500	-1.16130500
H	-1.79997000	-2.66013100	-2.34955500
C	-3.15458600	-3.28294100	1.25391800
H	-1.51313600	-2.03840000	1.90502000
C	-3.79284800	-3.82110000	0.13856500
H	-3.87296000	-4.05523600	-1.99407800
H	-3.55322000	-3.47415900	2.24305600
N	-5.00837900	-4.65562300	0.34737600
O	-5.36522700	-4.82864800	1.50000700
O	-5.54940700	-5.09917400	-0.64998600
O	1.83990800	-2.06635400	0.41485700
H	0.63275700	-2.01870700	-0.07592500
H	1.95506700	-2.67940500	1.15293000
Ti	2.55792200	-0.29184100	-0.06665000

P_{Ti}^{DA}
Charge = 2, Multiplicity = 1
Energy = -2261.1351204 hartree

O	-3.52749900	-1.88999700	-0.35713800
C	1.93532900	-2.00327400	2.61051400
H	1.99424600	-2.38547300	3.63567300
H	2.27906700	-0.96396000	2.62556400
C	-0.23826700	-4.32430300	1.62741500
H	0.74181500	-4.80287700	1.67713800
H	-0.97866400	-5.08420400	1.90321900
C	-0.33094100	-3.13825200	2.58314700
H	-1.35704200	-2.76738100	2.61974200
H	-0.04493700	-3.44055400	3.59688900
C	0.20698100	-4.01778100	-2.13693000
H	0.70234500	-4.64721600	-2.88471300
H	-0.77417900	-3.74220100	-2.52835700
C	2.80160500	-2.82414700	1.66605300
H	3.86503400	-2.68146800	1.89285400
H	2.59468600	-3.89295700	1.75095600
C	2.45063400	-2.83600100	-2.10452600
H	2.80955700	-1.84388300	-2.39691400
H	2.71733600	-3.53483500	-2.90530600
C	0.04991000	-4.77777100	-0.82235700
H	-0.63413100	-5.62327100	-0.96065900
H	1.00144800	-5.19260000	-0.48346700
C	3.07806200	-3.24819700	-0.78095700
H	2.86868800	-4.29259000	-0.54018000
H	4.16834700	-3.13666100	-0.81642500
N	0.96913700	-2.73638800	-1.95284200
H	0.62223200	-2.09491300	-2.66746500
H	-4.34987400	-1.70896600	-0.84177000
N	2.48453900	-2.39572500	0.28033900
H	2.86821800	-1.45552100	0.16029600
N	0.52354700	-1.99049600	2.12528800
H	0.07648600	-1.14469200	2.48199600
N	-0.44362900	-3.84598500	0.23087000
H	-1.45101800	-3.73337700	0.09246000
C	-6.08083400	2.63590200	0.18973000
C	-5.88000100	2.35028700	-1.15825900
C	-4.75451800	1.61401500	-1.52857500
C	-3.88036200	1.19845000	-0.52924700
C	-4.06748100	1.49149500	0.81909100
C	-5.19742900	2.22282600	1.18371300
H	-6.59291600	2.70274300	-1.89429900
H	-4.55039500	1.38395200	-2.56908900
H	-3.34722800	1.17313500	1.56607400
H	-5.39632200	2.47979000	2.21733200
N	-7.28432900	3.41695200	0.58474700
O	-7.42500000	3.64729900	1.77361900
O	-8.03480500	3.76256200	-0.31065000
O	-2.72153900	0.48606000	-0.95152900
P	-2.26010100	-0.94894600	-0.50167600
O	-1.20850500	-1.48890200	-1.49167100
O	-1.45802200	-1.04159700	0.81238900
O	1.69443800	2.35520400	-0.74137900
C	3.01301400	2.73554200	-0.50085200
C	3.76903500	3.38859800	-1.47464600
C	3.54540300	2.41541200	0.74979900
C	5.09378300	3.71961200	-1.19435900
H	3.33840600	3.63860900	-2.44111600
C	4.87208600	2.73567700	1.02509900
H	2.91079500	1.96538600	1.50744600
C	5.62702200	3.37667700	0.04533600
H	5.71348100	4.23039700	-1.92187500
H	5.32310000	2.51607300	1.98541400
N	7.04386000	3.70187200	0.33478200
O	7.48325100	3.34518400	1.41718700
O	7.66690200	4.29133500	-0.53153300
O	1.05933500	-0.14381400	-0.29295200
H	1.31441600	2.84869600	-1.48362400
H	1.26146100	0.82643200	-0.46929100
Ti	0.31129800	-1.69492300	-0.08325100

R_{Ti}^{WA}
Charge = 2, Multiplicity = 1
Energy = -2261.1271418 hartree

O	-2.48815500	0.53501700	0.10133400
C	-0.54793900	-3.66240200	-1.38581000
H	-0.25520900	-4.71933500	-1.37297700
H	0.19376100	-3.11728600	-1.97440400
C	-1.12438700	-3.09705200	2.32440900
H	-1.42095100	-3.69355500	3.19436400
H	-0.19880100	-2.57220900	2.56993000
C	-0.92648000	-3.97278200	1.09201600
H	-0.15555200	-4.73019600	1.27985700
H	-1.84339800	-4.50790900	0.82565200
C	-4.55825700	-1.73021300	1.53247300
H	-5.55753600	-2.17975500	1.55933000
H	-4.62041400	-0.75777500	2.03076300
C	-1.92744400	-3.50930200	-1.99904400
H	-1.92496700	-3.82562800	-3.04840400
H	-2.64778000	-4.14215500	-1.47416000
C	-4.63081200	-2.38769500	-0.91475800
H	-5.71101900	-2.25445200	-1.04876500
H	-4.46337600	-3.42827000	-0.62290100
C	-3.54315100	-2.61591900	2.22839000

H	-3.76390600	-2.71401800	3.29720500
H	-3.56167300	-3.62147700	1.79955700
C	-3.87631500	-2.02122600	-2.18849100
H	-4.14371500	-2.68211800	-3.02047800
H	-4.11529200	-0.99547700	-2.48837400
N	-4.10307800	-1.49192000	0.13832100
H	-4.38710700	-0.54790700	-0.11788300
H	-2.90680900	1.47058000	0.15840500
N	-2.41121800	-2.09148400	-1.90040100
H	-1.90148600	-1.53947400	-2.59437100
N	-0.54839500	-3.07302300	-0.02336600
H	0.40858900	-2.77549600	0.16812400
N	-2.16682400	-2.05956900	2.02932500
H	-2.02811500	-1.28957500	2.68918400
C	-1.00348300	4.64892200	-0.10765600
C	-1.03657500	3.86099400	-1.25769400
C	-0.20313700	2.75027200	-1.32678300
C	0.59350000	2.45418400	-0.22212800
C	0.68774600	3.28213700	0.89197400
C	-0.11882100	4.41928800	0.94295800
H	-1.70414800	4.11235700	-2.07442300
H	-0.18646000	2.11523500	-2.20519200
H	1.36120900	3.03307500	1.70465900
H	-0.09676500	5.08884700	1.79584000
N	-2.02149300	5.69990700	0.04176200
O	-1.73115100	6.72139900	0.61808200
O	-3.14276900	5.42618400	-0.41268100
O	1.35816500	1.26903100	-0.23052100
P	0.73100000	-0.18076800	-0.15310400
O	-0.28352800	-0.53575400	-1.26682000
O	-0.11704500	-0.48336200	1.10388300
O	1.98571200	-1.13339900	-0.24878300
C	3.37742900	-0.83609300	-0.14740700
C	3.95088700	-0.74015200	1.11594900
C	4.10482200	-0.72013100	-1.32622300
C	5.32233500	-0.50429000	1.20246300
H	3.34855400	-0.85433900	2.01142300
C	5.47648800	-0.48595200	-1.23537700
H	3.61673000	-0.81637100	-2.29018500
C	6.05689500	-0.38083100	0.02593700
H	5.82401200	-0.41988800	2.15903800
H	6.09345300	-0.38686000	-2.12045900
N	7.51972500	-0.12881200	0.12119600
O	8.13197900	-0.03027100	-0.92804000
O	7.99192100	-0.03891100	1.24173400
O	-3.78543100	2.72923000	0.23335700
H	-3.58787100	3.59557900	-0.18506400
H	-4.37321200	2.90749600	0.98014500
Ti	-1.81518900	-1.05498600	0.03180500

$\mathbf{T1}_{\mathbf{Ti}}^{\mathbf{WA}}$
Charge = 2, Multiplicity = 1
Energy = -2261.0571778 hartree

O	2.26812400	1.56461000	-1.36156300
C	2.40173200	-1.09804100	2.65276100
H	2.60439300	-1.86653900	3.40710900
H	1.42083600	-0.66610400	2.86179500
C	3.22446800	-3.15705900	-0.42817900
H	3.88223800	-3.99730300	-0.67640700
H	2.21863600	-3.39572300	-0.78483600
C	3.20692800	-2.88466800	1.06882600
H	2.82362900	-3.75020800	1.62151600
H	4.20983000	-2.67154100	1.45209300
C	5.47461200	-0.35909800	-1.63398000
H	6.55102800	-0.16020200	-1.58617800
H	5.15758800	-0.20569400	-2.67095500
C	3.47213500	-0.01897600	2.68334600
H	3.47654000	0.50308600	3.64707100
H	4.46514100	-0.45847600	2.54913200
C	5.42146100	1.07050600	0.43447700
H	6.26435200	1.71798300	0.16566600
H	5.83585500	0.20199500	0.95467400
C	5.14968900	-1.77251800	-1.18539600
H	5.58608700	-2.51571400	-1.86245600
H	5.55000300	-1.96603900	-0.18589500
C	4.42744300	1.81999300	1.31659800
H	4.89610700	2.12864300	2.25819900
H	4.08263100	2.73082000	0.81543200
N	4.70679000	0.59466700	-0.78195000
H	4.49484100	1.40928500	-1.35757900
H	2.45749600	2.48324900	-1.12474300
N	3.23959700	0.94913100	1.56552900
H	2.43696800	1.52918400	1.81869100
N	2.34844700	-1.68903700	1.28515100
H	1.38443500	-1.97891300	1.11183100
N	3.66646400	-1.91222600	-1.12913000
H	3.30879500	-1.95626700	-2.08770200
C	-2.83472400	4.38763300	0.07363800
C	-2.04209100	3.84970700	1.08649800
C	-1.62593800	2.52386000	0.98881800
C	-2.01854000	1.77281100	-0.12266700
C	-2.83472900	2.31004500	-1.12500300
C	-3.24744400	3.64097700	-1.02536800
H	-1.77094800	4.46925400	1.93332300
H	-1.02455700	2.06654500	1.76582400
H	-3.18387600	1.67941000	-1.93832300
H	-3.88707800	4.09369000	-1.77413500
N	-3.25442700	5.81067700	0.17538200
O	-3.96359500	6.24186500	-0.71612400
O	-2.84614300	6.43669700	1.13956100
O	-1.64906100	0.43439500	-0.23917100
P	-0.17333400	-0.18777500	-0.57883400
O	0.73224600	0.34842600	0.58713100
O	1.24507900	-0.80046300	-1.34128100
O	-0.72139100	-1.70196600	-0.52922300
C	-2.02538900	-2.19240700	-0.28015100
C	-2.73757600	-2.69028700	-1.36720200
C	-2.50639200	-2.25598700	1.02512600
C	-3.98762000	-3.26508900	-1.14151300
H	-2.31830200	-2.63950000	-2.36668300
C	-3.75434200	-2.83224900	1.25008300
H	-1.92666900	-1.85815900	1.85170500

C	-4.47228500	-3.32406800	0.16229300
H	-4.58134100	-3.66690900	-1.95388500
H	-4.17538300	-2.90472100	2.24567400
N	-5.80451300	-3.93620700	0.40495500
O	-6.19209700	-3.97527800	1.56092000
O	-6.40801900	-4.35110800	-0.56956300
O	-0.03553700	1.00869000	-1.97033900
H	0.97833700	1.39391500	-1.91264100
H	-0.72747900	1.70176100	-2.01096900
Ti	2.55491500	-0.09775800	-0.32746400

$I1_{Ti}^{WA}$
Charge = 2, Multiplicity = 1
Energy = -2261.0771915 hartree

O	2.33031300	1.61625200	-1.70321000
C	2.20551900	-0.84142900	2.65568700
H	2.34005500	-1.54842100	3.48183000
H	1.22110700	-0.38075300	2.75809100
C	3.23343700	-3.17552100	-0.15438100
H	3.91346800	-4.02599100	-0.27414100
H	2.25752000	-3.46035700	-0.55769400
C	3.10346500	-2.76150400	1.30320200
H	2.67683600	-3.56832300	1.90991700
H	4.07532300	-2.50484400	1.73635500
C	5.51259000	-0.46724400	-1.51234500
H	6.58335200	-0.24529100	-1.44321800
H	5.23476900	-0.42535800	-2.57078200
C	3.29433200	0.22228900	2.67659400
H	3.24889600	0.80862200	3.60149500
H	4.28465100	-0.24130600	2.63569000
C	5.36881000	1.11168500	0.42753200
H	6.23760900	1.72078300	0.15236200
H	5.74060500	0.27055900	1.01980800
C	5.19087600	-1.83551700	-0.93510800
H	5.66865100	-2.62994000	-1.52013300
H	5.55327400	-1.92582700	0.09337300
C	4.36018500	1.93685100	1.21930900
H	4.80623500	2.30465900	2.15094400
H	4.05109200	2.81627400	0.64355800
N	4.70183800	0.56367600	-0.79195900
H	4.56823500	1.33834500	-1.44299000
H	2.47162200	2.56916400	-1.59831100
N	3.14832200	1.10922100	1.48196000
H	2.34400800	1.71817400	1.64204400
N	2.23422500	-1.55266800	1.34167400
H	1.28174900	-1.85772800	1.12886600
N	3.71076900	-1.99248100	-0.92994200
H	3.39673300	-2.11044900	-1.89696400
C	-2.91233500	4.39287100	0.10546300
C	-1.95385400	3.93302300	1.00694700
C	-1.51607500	2.61410200	0.91646400
C	-2.04830600	1.78741100	-0.07765400
C	-3.02965100	2.24920600	-0.96122800
C	-3.46750900	3.57122900	-0.87075000
H	-1.57454600	4.60707000	1.76594000
H	-0.78798000	2.21839600	1.61437900
H	-3.47742700	1.56718800	-1.67927300
H	-4.22980300	3.96317400	-1.53388800
N	-3.35444500	5.80716600	0.19420800
O	-4.20510400	6.17279900	-0.59809100
O	-2.81403600	6.50227600	1.04028300
O	-1.65824200	0.45727100	-0.19369700
P	-0.20487400	-0.11262800	-0.72774900
O	0.76918600	0.48182500	0.38343300
O	1.35878400	-0.80678400	-1.41451100
O	-0.65437500	-1.65567500	-0.58374200
C	-1.93380700	-2.20417200	-0.31601400
C	-2.62900300	-2.75964300	-1.38527900
C	-2.39989800	-2.25803300	0.99428000
C	-3.84698200	-3.39055800	-1.13405800
H	-2.22138000	-2.71189300	-2.38979000
C	-3.61582900	-2.89019800	1.24439400
H	-1.83877800	-1.80305200	1.80391700
C	-4.31663100	-3.44415600	0.17550800
H	-4.42643800	-3.84050900	-1.93137600
H	-4.02603100	-2.95667100	2.24490000
N	-5.61268500	-4.11900600	0.44587800
O	-5.99461300	-4.13777200	1.60429600
O	-6.20854600	-4.57646000	-0.51411100
O	-0.20339300	0.75023600	-2.12976800
H	1.46628500	1.47808600	-2.16274100
H	-1.02009400	1.25504200	-2.30544400
Ti	2.55007000	-0.11803000	-0.35234800

$I2_{Ti}^{WA}$
Charge = 2, Multiplicity = 1
Energy = -2261.0728301 hartree

O	-0.42348400	-0.67864700	1.84400600
C	4.98126900	1.31096500	2.01777900
H	5.67713400	0.99824000	2.80470700
H	4.69066400	2.34423500	2.23615600
C	4.25036500	-1.84350500	1.38717700
H	5.28347300	-1.66783800	1.07781100
H	4.17921300	-2.89247200	1.69457100
C	3.84989500	-0.91619800	2.52697500
H	2.86459700	-1.19470600	2.91356700
H	4.56427900	-0.98749700	3.35514500
C	3.15399900	-1.45715100	-2.25712500
H	3.66076400	-1.68072200	-3.20302400
H	2.16527500	-1.91936100	-2.29000000
C	5.64439900	1.23217500	0.65006000
H	6.45077700	1.96995900	0.56476700
H	6.08849800	0.24823800	0.48200600
C	3.94874500	0.90993900	-2.72906400
H	3.45595900	1.86195200	-2.95625400
H	4.30747700	0.50810300	-3.68435800
C	3.95289300	-2.02712800	-1.08937600
H	3.96922000	-3.12188600	-1.14446800
H	4.99195200	-1.69055500	-1.11286200
C	5.11657800	1.13155500	-1.77545700
H	5.72787200	0.22946200	-1.70070300
H	5.77202900	1.92857600	-2.14476300

N	2.92941900	0.01749100	-2.10987100
H	2.02623300	0.20583100	-2.54376700
H	-1.23963400	-1.21498300	1.87625300
N	4.60697400	1.44512000	-0.40435500
H	4.36202200	2.43928700	-0.38129600
N	3.73619100	0.49010400	2.03574600
H	3.05674000	0.95290500	2.63965100
N	3.36636600	-1.57351800	0.20959800
H	2.48692200	-2.08098500	0.34479900
C	-3.35559500	-4.10618400	-0.09418900
C	-2.28682600	-3.93945000	-0.97367100
C	-1.66449000	-2.69628200	-1.05081800
C	-2.12904100	-1.65053000	-0.24803900
C	-3.21745700	-1.81957100	0.61535200
C	-3.83793700	-3.06792800	0.69621100
H	-1.96582900	-4.77589800	-1.58315300
H	-0.84447000	-2.52271100	-1.73901200
H	-3.60209500	-0.97257000	1.17689900
H	-4.68741000	-3.23800300	1.34730700
N	-4.00416600	-5.43972500	-0.00368500
O	-4.93972000	-5.54860700	0.76955300
O	-3.54443600	-6.32562700	-0.70585300
O	-1.54011400	-0.39390700	-0.32943300
P	-0.18050700	0.10567800	0.46196700
O	0.90484300	-0.43273100	-0.58102800
O	1.31359300	0.77230800	1.29035900
O	-0.56843000	1.69326200	0.26496300
C	-1.86725100	2.25099400	0.13015900
C	-2.51189400	2.67473500	1.28927900
C	-2.40289800	2.44152400	-1.14061700
C	-3.74819600	3.31029300	1.17281900
H	-2.04977700	2.52659500	2.26010100
C	-3.63502800	3.08182300	-1.25609400
H	-1.88032100	2.08210900	-2.02053100
C	-4.28419300	3.50289000	-0.09752300
H	-4.28946800	3.66163900	2.04321900
H	-4.09737000	3.25346100	-2.22078500
N	-5.59926900	4.18506000	-0.22408100
O	-6.03005900	4.34828000	-1.35319600
O	-6.14530700	4.52790100	0.81049700
O	1.89740900	2.42865000	-0.58000100
H	0.98372700	2.53948200	-0.20811000
H	1.96985500	2.93357900	-1.40351200
Ti	2.55647000	0.48885400	0.09021400

$\mathbf{T2_{Ti}^{WA}}$
Charge = 2, Multiplicity = 1
Energy = -2261.0604496 hartree

O	-0.39521900	1.08210300	-1.92800500
C	5.04816700	-1.21192500	-1.85924900
H	5.76841400	-0.93654000	-2.63788900
H	4.75472500	-2.25143900	-2.04084000
C	4.36330600	1.96161200	-1.34534300
H	5.38264300	1.77358500	-1.00106200
H	4.32620000	3.00104900	-1.68947400
C	3.97016300	1.01048800	-2.46975500
H	3.00202000	1.29888900	-2.88905400
H	4.70884600	1.04173000	-3.27885600
C	3.10683600	1.70996100	2.25374000
H	3.56952300	1.93207800	3.22209600
H	2.13115700	2.20000900	2.22953300
C	5.66817900	-1.08034000	-0.47499800
H	6.45766100	-1.82745300	-0.33053700
H	6.12339400	-0.09789900	-0.33151000
C	3.81254900	-0.66504100	2.80468200
H	3.28968600	-1.60765100	2.99992000
H	4.11150400	-0.25960600	3.77858100
C	3.97812500	2.23687300	1.11743100
H	4.01031000	3.33238000	1.14374300
H	5.00830800	1.88391500	1.20237700
C	5.03330000	-0.90874500	1.92685000
H	5.66102300	-0.01650900	1.87420800
H	5.65509300	-1.71073700	2.34119500
N	2.84975200	0.23950200	2.11410600
H	1.91981000	0.07691400	2.49996200
H	-1.25726700	1.54182000	-1.93303600
N	4.58736700	-1.23373300	0.53972000
H	4.30880300	-2.21842500	0.52557500
N	3.81089000	-0.38322500	-1.94976200
H	3.15509300	-0.85005400	-2.57726700
N	3.44147000	1.75005900	-0.18685100
H	2.57552300	2.26505500	-0.37243700
C	-3.91286800	3.76901000	0.10425000
C	-2.83099000	3.71122100	0.98142700
C	-2.02179100	2.57813000	0.97925800
C	-2.31877500	1.53283400	0.10016500
C	-3.41516600	1.59029100	-0.76646500
C	-4.22409700	2.72861200	-0.76531500
H	-2.64301400	4.54349700	1.64934400
H	-1.18120700	2.48953400	1.65892500
H	-3.65379100	0.74090100	-1.39996700
H	-5.08724000	2.81171200	-1.41535200
N	-4.76575400	4.98665500	0.10173500
O	-5.70691500	5.00028300	-0.67209500
O	-4.45291300	5.88001800	0.87126300
O	-1.54252600	0.37418900	0.10997100
P	-0.08209100	0.18265200	-0.63456500
O	0.87435100	0.68021100	0.53457000
O	1.34500600	-0.35201900	-1.41971500
O	-0.35881100	-1.56893700	-0.46253700
C	-1.56833300	-2.29769300	-0.23798000
C	-2.19878000	-2.83314300	-1.35536500
C	-2.01803500	-2.49931100	1.06286600
C	-3.34136700	-3.61026500	-1.16130500
H	-1.79997000	-2.66013100	-2.34955500
C	-3.15458600	-3.28294100	1.25391800
H	-1.51313600	-2.03840000	1.90502000
C	-3.79284800	-3.82110000	0.13856500
H	-3.87296000	-4.05523600	-1.99407800
H	-3.55322000	-3.47415900	2.24305600
N	-5.00837900	-4.65562300	0.34737600
O	-5.36522700	-4.82864800	1.50000700

O	-5.54940700	-5.09917400	-0.64998600
O	1.83990800	-2.06635400	0.41485700
H	0.63275700	-2.01870700	-0.07592500
H	1.95506700	-2.67940500	1.15293000
Ti	2.55792200	-0.29184100	-0.06665000

$\mathbf{P}_{\mathbf{Ti}}^{\mathbf{WA}}$

Charge = 2, Multiplicity = 1

Energy = -2261.1351204 hartree

O	-3.52749900	-1.88999700	-0.35713800
C	1.93532900	-2.00327400	2.61051400
H	1.99424600	-2.38547300	3.63567300
H	2.27906700	-0.96396000	2.62556400
C	-0.23826700	-4.32430300	1.62741500
H	0.74181500	-4.80287700	1.67713800
H	-0.97866400	-5.08420400	1.90321900
C	-0.33094100	-3.13825200	2.58314700
H	-1.35704200	-2.76738100	2.61974200
H	-0.04493700	-3.44055400	3.59688900
C	0.20698100	-4.01778100	-2.13693000
H	0.70234500	-4.64721600	-2.88471300
H	-0.77417900	-3.74220100	-2.52835700
C	2.80160500	-2.82414700	1.66605300
H	3.86503400	-2.68146800	1.89285400
H	2.59468600	-3.89295700	1.75095600
C	2.45063400	-2.83600100	-2.10452600
H	2.80955700	-1.84388300	-2.39691400
H	2.71733600	-3.53483500	-2.90530600
C	0.04991000	-4.77777100	-0.82235700
H	-0.63413100	-5.62327100	-0.96065900
H	1.00144800	-5.19260000	-0.48346700
C	3.07806200	-3.24819700	-0.78095700
H	2.86868800	-4.29259000	-0.54018000
H	4.16834700	-3.13666100	-0.81642500
N	0.96913700	-2.73638800	-1.95284200
H	0.62223200	-2.09491300	-2.66746500
H	-4.34987400	-1.70896600	-0.84177000
N	2.48453900	-2.39572500	0.28033900
H	2.86821800	-1.45552100	0.16029600
N	0.52354700	-1.99049600	2.12528800
H	0.07648600	-1.14469200	2.48199600
N	-0.44362900	-3.84598500	0.23087000
H	-1.45101800	-3.73337700	0.09246000
C	-6.08083400	2.63590200	0.18973000
C	-5.88000100	2.35028700	-1.15825900
C	-4.75451800	1.61401500	-1.52857500
C	-3.88036200	1.19845000	-0.52924700
C	-4.06748100	1.49149500	0.81909100
C	-5.19742900	2.22282600	1.18371300
H	-6.59291600	2.70274300	-1.89429900
H	-4.55039500	1.38395200	-2.56908900
H	-3.34722800	1.17313500	1.56607400
H	-5.39632200	2.47979000	2.21733200
N	-7.28432900	3.41695200	0.58474700
O	-7.42500000	3.64729900	1.77361900
O	-8.03480500	3.76256200	-0.31065000
O	-2.72153900	0.48606000	-0.95152900
P	-2.26010100	-0.94894600	-0.50167600
O	-1.20850500	-1.48890200	-1.49167100
O	-1.45802200	-1.04159700	0.81238900
O	1.69443800	2.35520400	-0.74137900
C	3.01301400	2.73554200	-0.50085200
C	3.76903500	3.38859800	-1.47464600
C	3.54540300	2.41541200	0.74979900
C	5.09378300	3.71961200	-1.19435900
H	3.33840600	3.63860900	-2.44111600
C	4.87208600	2.73567700	1.02509900
H	2.91079500	1.96538600	1.50744600
C	5.62702200	3.37667700	0.04533600
H	5.71348100	4.23039700	-1.92187500
H	5.32310000	2.51607300	1.98541400
N	7.04386000	3.70187200	0.33478200
O	7.48325100	3.34518400	1.41718700
O	7.66690200	4.29133500	-0.53153300
O	1.05933500	-0.14381400	-0.29295200
H	1.31441600	2.84869600	-1.48362400
H	1.26146100	0.82643200	-0.46929100
Ti	0.31129800	-1.69492300	-0.08325100
